# A click chemistry approach to pleuromutilin derivatives, evaluation of anti-MRSA activity and elucidation of binding mode by surface plasmon resonance and molecular docking

**DOI:** 10.1080/14756366.2021.1977931

**Published:** 2021-11-25

**Authors:** Zhe Zhang, Zhao-Sheng Zhang, Xiao Wang, Gao-Lei Xi, Zhen Jin, You-Zhi Tang

**Affiliations:** a Guangdong Provincial Key Laboratory of Veterinary Pharmaceutics Development and Safety Evaluation, College of Veterinary Medicine, South China Agricultural University, Guangzhou, China; b Technology Center for China Tobacco Henan Industrial Limited Company, Zhengzhou, China; c Guangdong Laboratory for Lingnan Modern Agriculture, Guangzhou, China

**Keywords:** Pleuromutilin, 1,2,3-triazole, MRSA, SPR, Molecular docking

## Abstract

Novel series of pleuromutilin analogs containing substituted 1,2,3-triazole moieties were designed, synthesised and assessed for their *in vitro* antibacterial activity against Methicillin-resistant *Staphylococcus aureus* (MRSA). Initially, the *in vitro* antibacterial activities of these derivatives against 4 strains of *S. aureus* (MRSA ATCC 43300, ATCC 29213, AD3, and 144) were tested by the broth dilution method. Most of the synthesised pleuromutilin analogs displayed potent activities. Among them, compounds **50**, **62**, and **64** (MIC = 0.5∼1 µg/mL) showed the most effective antibacterial activity and their anti-MRSA activity were further studied by the time-killing kinetics approach. Binding mode investigations by surface plasmon resonance (SPR) with *50S* ribosome revealed that the selected compounds all showed obvious affinity for *50S* ribosome (K_D_ = 2.32 × 10^−8^∼5.10 × 10^−5^ M). Subsequently, the binding of compounds **50** and **64** to the *50S* ribosome was further investigated by molecular modelling. Compound **50** had a superior docking mode with *50S* ribosome, and the binding free energy of compound **50** was calculated to be −12.0 kcal/mol.

## Graphical Abstract

**Figure:**
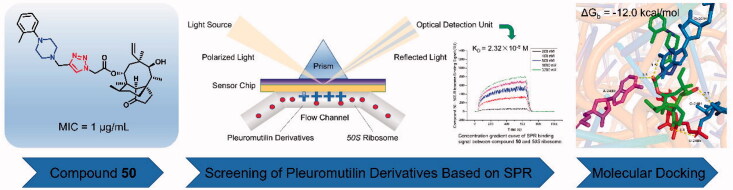

## 1. Introduction

Methicillin-resistant *Staphylococcus aureus* (MRSA) is a bacterium with a broad spectrum of drug resistance^1^^,^^2^. The prevalence of MRSA infection had become a serious medical problem in hospital and community settings^3^. Indeed, in the United States (US), pneumonia infected by MDR gram-positive bacteria caused at least 1.5 million hospitalisations annually, resulting in approximately 100,000 deaths^4^. Therefore, there is an urgent need to develop new antibiotics that exhibit minimal cross-resistance with existing drug treatments to treat infections caused by MRSA.

**Figure 1.**
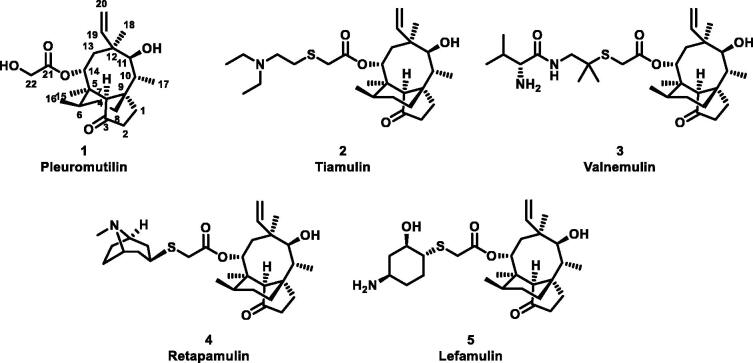
Structure of pleuromutilin (1), tiamulin (2), valnemulin (3), retapamulin (4) and lefamulin (5).

The naturally tricyclic diterpene product, (+)-pleuromutilin (**1**, Figure 1), was first isolated from the higher fungi Basidiomycetes Pleurotus species *Pleurotus mutiliz* and *Pleurotus Passeckeranius* in 1951^5^. The pleuromutilin class displays potent antibacterial activity especially against gram-positive bacteria and *mycoplasmas*^6^. Further studies have identified that pleuromutilin and its analogues could selectively inhibit bacterial protein synthesis through interaction with the peptidyl transferase centre (PTC) of the bacterial *50S* ribosomal subunit *23S* rRNA^7–11^. This distinct mechanism of action of pleuromutilin makes it possesses rarely cross-resistance with other classes of clinically used antibacterial drugs, which has inspired researchers to modify its structure to obtain new antibiotics^12^. The structural optimizations of its C-14 side chain prompted the discovery of tiamulin (**2**, Figure 1) and valnemulin (**3**, Figure 1), which were successively approved by the Food and Drug Administration (FDA)^13^. During the past ten years, retapamulin (**4**, Figure 1) was approved as a topical antibiotic for human use to treat skin infections in 2009^14^, while lefamulin (**5**, Figure 1) was approved by the FDA as the first intravenous and oral pleuromutilin antibiotic in 2019^15^^,^^16^.

Previous studies on the structure-activity relationship (SAR) have shown that the C-14 side chain of pleuromutilin derivatives containing thioether bonds, basic groups, or heterocyclic rings containing polar groups can improve their antibacterial activity^17–23^. In our previous work, some novel pleuromutilin analogues which having a piperazine as linker displayed potent *in vitro* and *in vivo* antibacterial activity against *S. aureus* (ATCC 29213)^20^^,^^21^. As an important pharmacophore, 1*H*-1,2,3-triazole have been used in many drugs with antibacterial, *β*-lactamase inhibitory, anti-inflammatory, antiviral and anticonvulsant activities^24^^,^^25^.

Given the above survey results, we now report the design, synthesis, and anti-MRSA activity of novel pleuromutilin derivatives with 1,2,3-triazole substituents incorporated into the C-14 side chain. These derivatives were synthesised by a click chemistry strategy using Cu(I)-catalyzed alkyne-azide [3 + 2] cycloaddition reaction (CuAAC reaction). Furthermore, the binding mode of the potent derivatives were investigated using surface plasmon resonance (SPR), and molecular docking studies.

## 2. Results and discussion

### 2.1. Chemistry

**Scheme 1:**
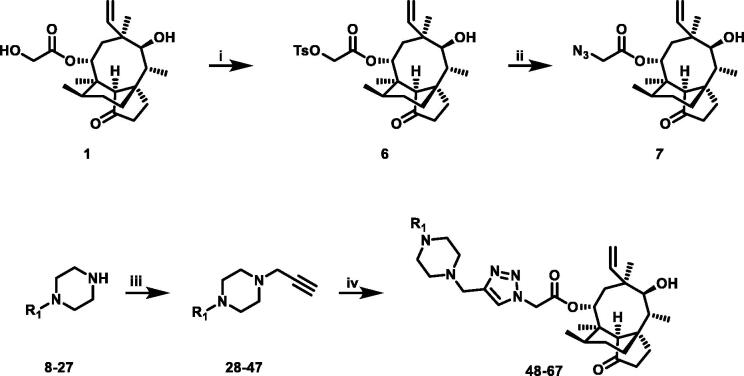
Reagent and conditions: (i) *p*-toluenesulfonyl chloride, ethyl acetate, NaOH, 0 °C for 0.5 h, rt for 3 h; (ii) sodium azide, acetone, H_2_O, reflux, 4 h; (iii) 3-Bromopropyne, DCM, K_2_CO_3_, rt, overnight; (iv) compound **7**, CuSO_4_·5H_2_O, sodium ascorbate, tert-Butanol: H_2_O = 1:1, rt, 3 h.

**Scheme 2:**
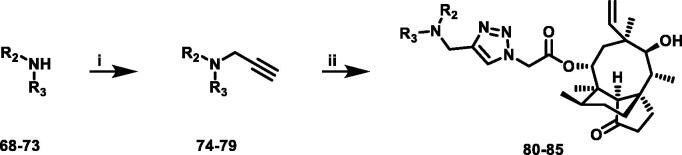
Reagent and conditions: (i) 3-bromopropyne, ethyl acetate, DIPEA, reflux, 6 h; (ii) compound **7**, CuSO_4_·5H_2_O, sodium ascorbate, tert-Butanol: H_2_O = 1:1, rt, 3 h.

The novel series of pleuromutilin derivatives was synthesised through a general click chemistry strategy (Schemes 1 and 2). As shown in Scheme 1, 22-*O*-tosylpleuromutilin (compound **6**) was prepared by the reaction of pleuromutilin **1** with *p*-toluenesulfonyl chloride in ethyl acetate. Then, compound **6** was converted into 22-*O*-azidoacetate-deoxypleuromutilin (compound **7**) through a nucleophilic substitution.

26 different terminal alkynes compounds (**28**–**47, 74**–**79**) were synthesised by various piperazine derivatives (compounds **8**–**27**) or secondary amines (compounds **68**–**73**) and propargyl bromide under alkaline conditions. A standard click reaction based on the catalysis Cu^+^, which was produced *in situ* by CuSO_4_·5H_2_O and sodium ascorbate, was applied for the cycloaddition^26^. The azide compound **7** was reacted with terminal alkynes compounds (**28**–**47, 74**–**79**) to give the target compounds **48**–**67**, **80**–**85** by a standard click reaction, respectively. All those pleuromutilin derivatives were purified by silica column chromatography and then confirmed by ^1^H NMR, ^13 ^C NMR and high-resolution mass spectral analysis (HR-MS).

### 2.2. In vitro antibacterial activity

All the synthesised 1,2,3-triazole linked pleuromutilin analogues were screened for *in vitro* antibacterial activities against methicillin-resistant *S. aureus* (ATCC 43300), *S. aureus* (ATCC 29213), and two clinical strains of *S. aureus* (AD3 and 144, isolated from Guangdong Province, China). The MICs and MBCs of the synthesised pleuromutilin derivatives along with pleuromutilin and tiamulin used as the reference antibacterial drugs, were determined by the broth micro dilution methods according to the Clinical and Laboratory Standards Institute (CLSI)^18^. The results of MIC and MBC were shown in Tables 1 and 2.

**Table 1.** *In vitro* antibacterial activity of the synthesised pleuromutilin derivatives **48–67.**

Compound No.	R_1_=	MIC (μg/mL) **/**MBC (μg/mL)			
		MRSA ATCC 43300	*S. aureus* ATCC 29213	*S. aureus* AD3	*S. aureus* 144
	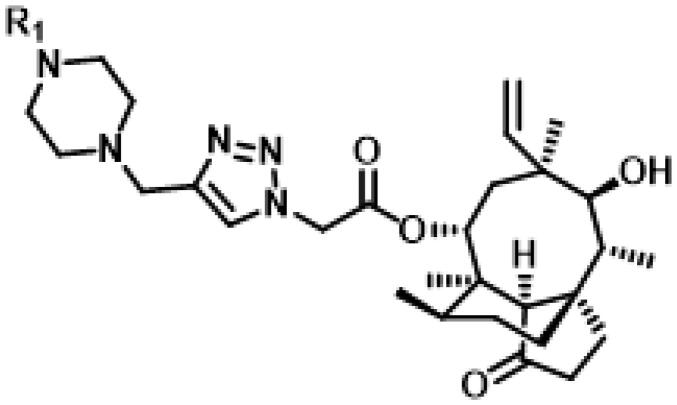				
				
48	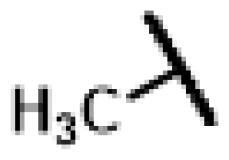	8/16	16/32	16/32	32/32		
49	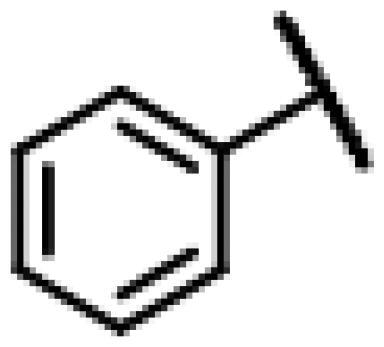	4/4	8/8	8/16	8/16		
50	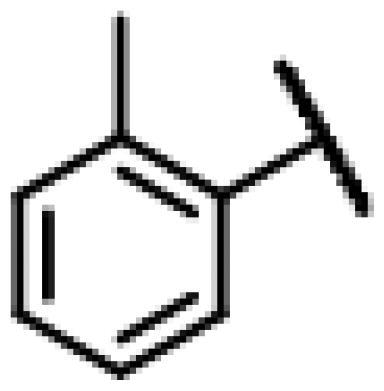	1/2	1/2	2/4	4/4		
51	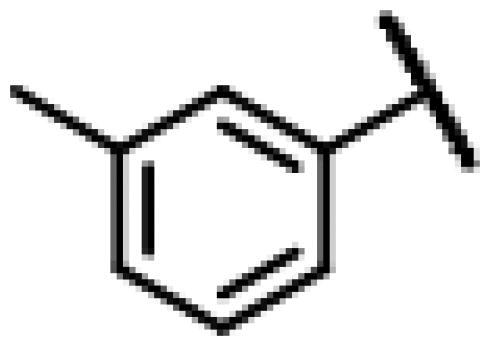	1/2	2/4	2/4	4/8		
52	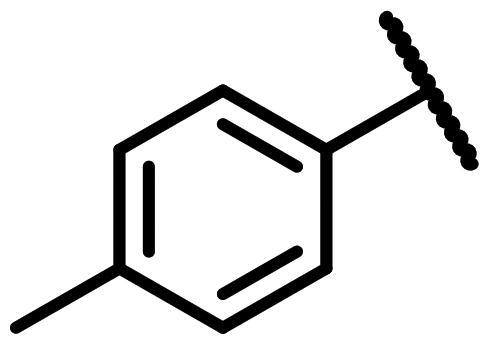	4/8	8/8	8/32	8/16		
53	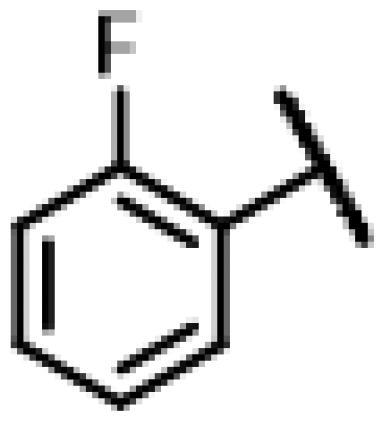	2/4	4/8	4/8	8/8		
54	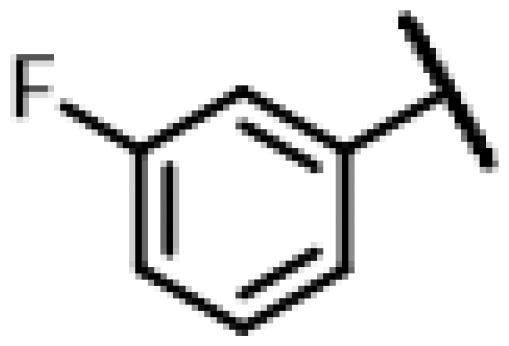	4/4	8/8	8/16	8/16		
55	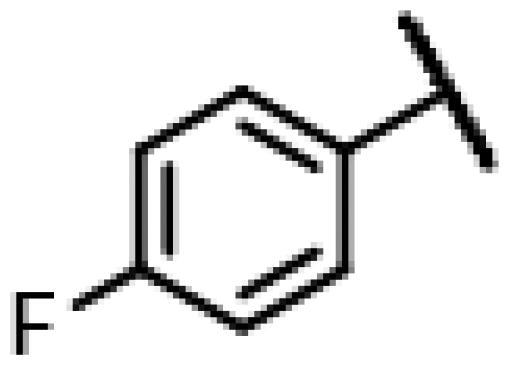	4/4	8/8	8/16	8/32		
56	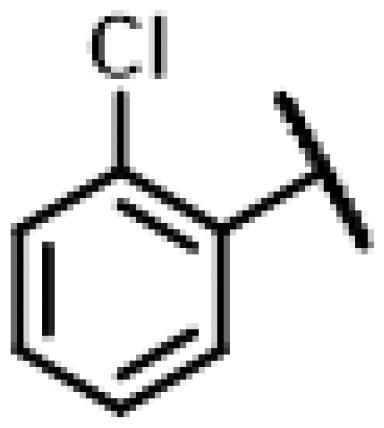	4/4	8/32	16/32	16/32		
57	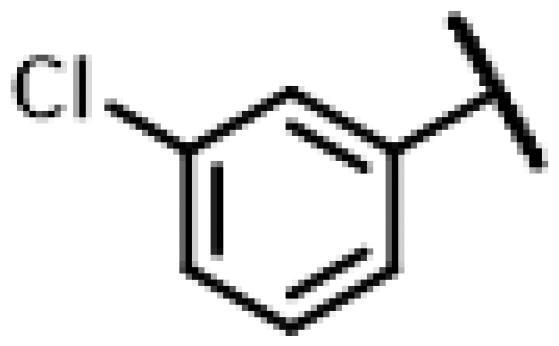	4/8	8/16	16/32	8/16		
58	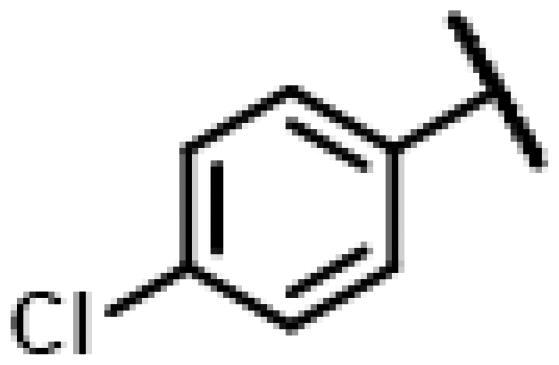	4/4	8/16	8/32	16/32		
59	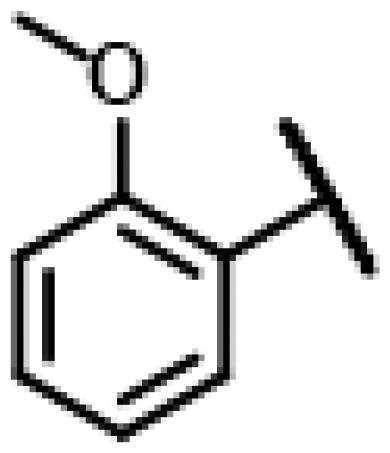	4/4	8/8	4/16	8/16		
60	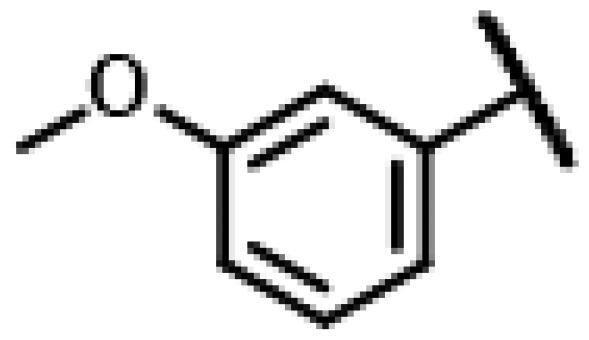	4/8	8/8	4/16	8/16		
61	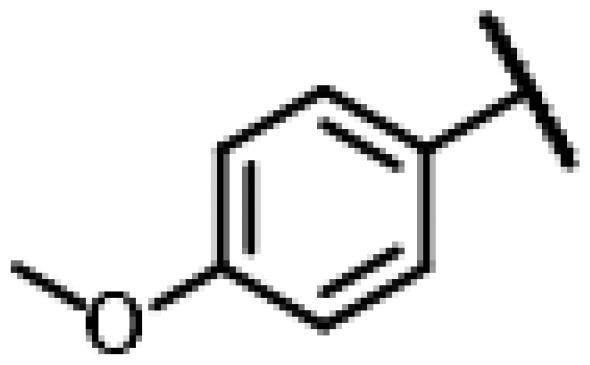	4/16	4/8	4/8	8/16		
62	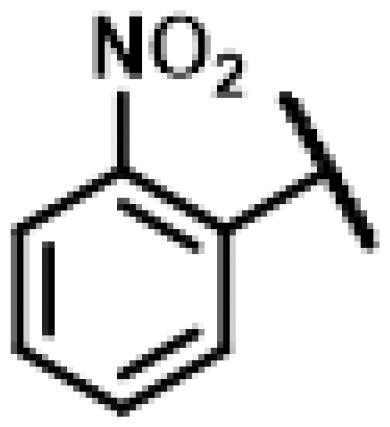	1/2	2/2	1/4	2/4		
63	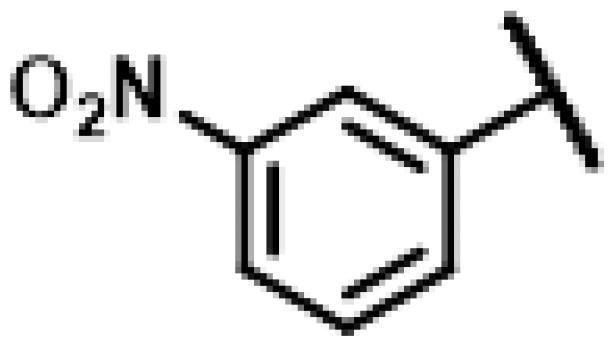	2/2	4/8	2/4	4/16		
64	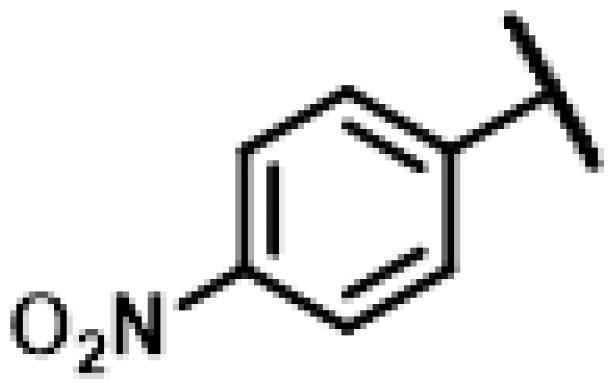	0.5/1	1/2	1/4	2/4		
65	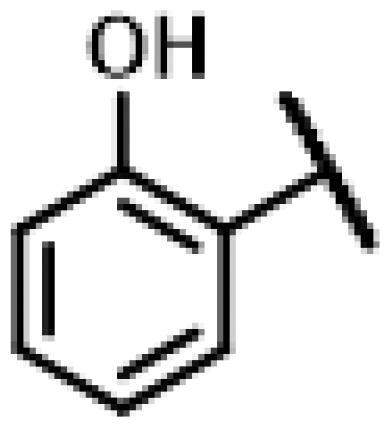	4/8	8/8	4/8	8/16		
66	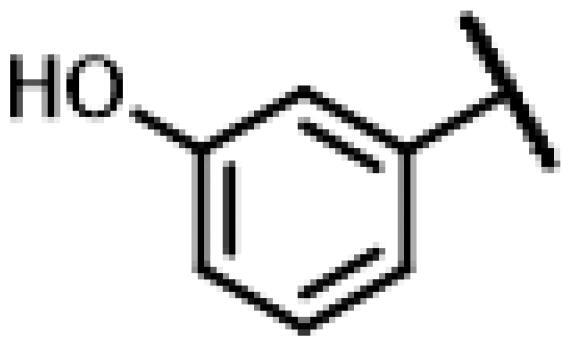	4/8	8/16	8/32	8/16		
67	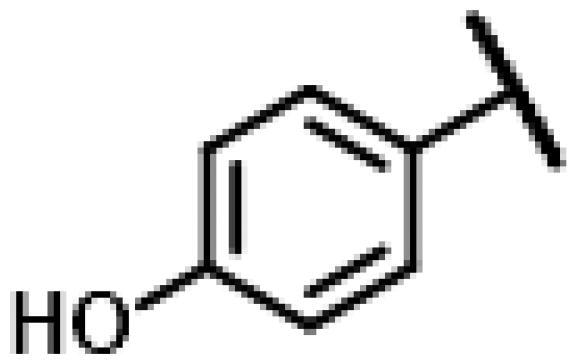	8/16	16/32	16/>32	32/32		
Pleuromutilin		2/4	4/8	2/4	2/4		
Tiamulin		0.5/1	0.5/1	1/2	1/2		

**Table 2:** *In vitro* antibacterial activity of the synthesised pleuromutilin derivatives **79–84.**

Compound No.	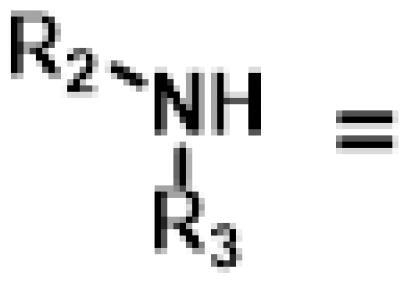	MIC (μg/mL) **/**MBC (μg/mL)			
		MRSA ATCC 43300	*S. aureus* ATCC 29213	*S. aureus* AD3	*S. aureus* 144
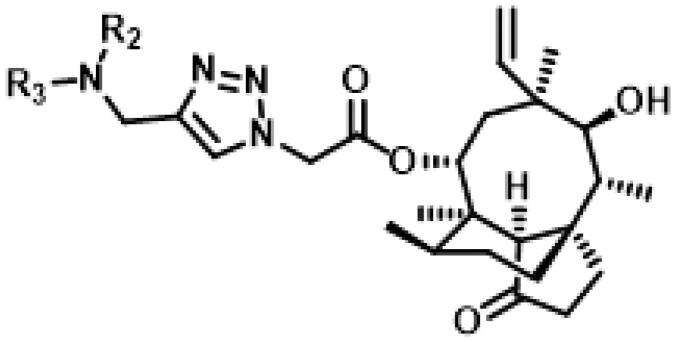					
80	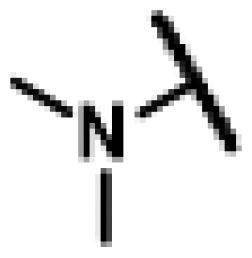	8/8	16/16	8/32	16/32
81	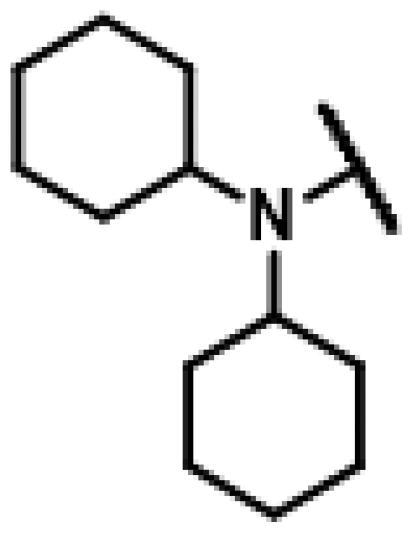	4/8	8/16	8/32	8/16
82	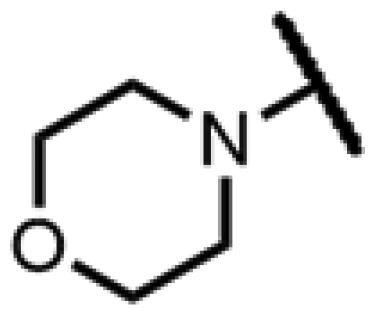	8/8	16/16	16/32	16/32
83	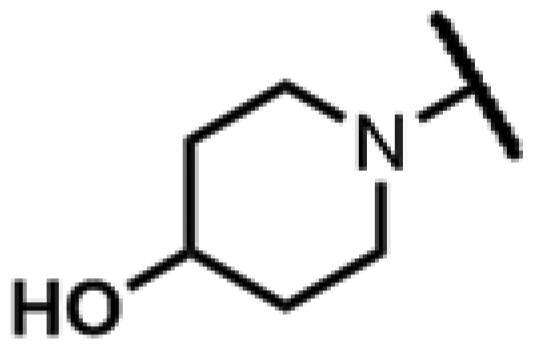	8/16	16/32	16/32	32/32
84	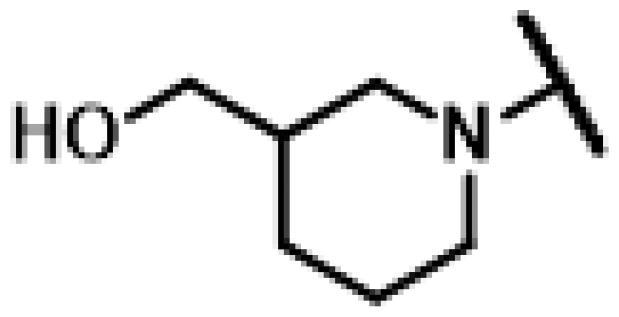	8/16	16/32	16/32	32/32
85	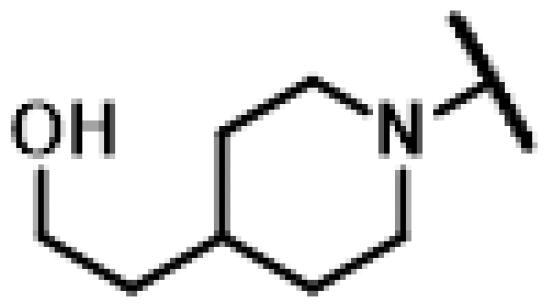	8/8	16/32	16/32	16/32
Pleuromutilin		2/4	4/8	2/4	2/4
Tiamulin		0.5/1	0.5/1	1/2	1/2

The MIC values of all these 26 compounds *in vitro* against MRSA (ATCC 43300), *S. aureus* (ATCC 29213), *S. aureus* (AD3) and *S. aureus* (144) ranged from 0.5 to 8 µg/mL, 1 to 16 µg/mL, 1 to 16 µg/mL and 2 to 32 µg/mL, respectively.

First of all, we borrowed from the previous work experience of the laboratory^18–20^^,^^22^ and introduced methylpiperazine and phenylpiperazine in the C-14 side chain. In order to explore SAR, different electron withdrawing groups (chlorine, fluorine, nitro and hydroxy) and donating groups (methyl and methoxy) were introduced on the benzene ring of compound **49**. Most of the derivatives of compound **49** reserved moderate to strong antibacterial activities, with compounds **50**, **56**, **62** and **64** displaying potent antibacterial activity against MRSA. The substituted piperazine derivatives **50**–**52**, **62**–**64** bearing optimum substituents, including methyl and nitro groups, exhibited relatively high inhibitory activities against all the tested strains. Among them, compound **64** which bearing 4-nitrophenyl piperazine group on the C-14 glycolic acid side chain possessed the highest antibacterial activities against MRSA (MIC = 0.5 µg/mL) in this series, being comparable to tiamulin. This may be explained by the strong electron-withdrawing ability of the nitro group, which can generate local electron-deficient sites in the molecule and interact with proteins and amino acids present in the living system^27^. However, other kinds of substituents or other substituent sites may weaken these effects.

Finally, compounds **79**–**84** were designed and synthesised by linking different azaheterocycles and dimethylamine with pleuromutilin via 1,2,3-triazole. Compounds **79**–**84** exhibited relatively moderate inhibitory activities against MRSA, which was lower than that of tiamulin.

The MBC referred to the minimum drug concentration required to kill 99.9% (reduced by 3 orders of magnitude) of the tested strains^19^^,^^28^. As shown in Tables 1 and 2, all synthesised pleuromutilin derivatives (MBC/MIC ≤ 4) had good bactericidal ability and all the tested strains had no drug resistance to the tested drugs. Among these derivatives, compounds **50**, **62** and **64** exhibited potent bactericidal effects, hence we carried out an in-depth study on the antibacterial activity of these three compounds.

**Figure 2.**
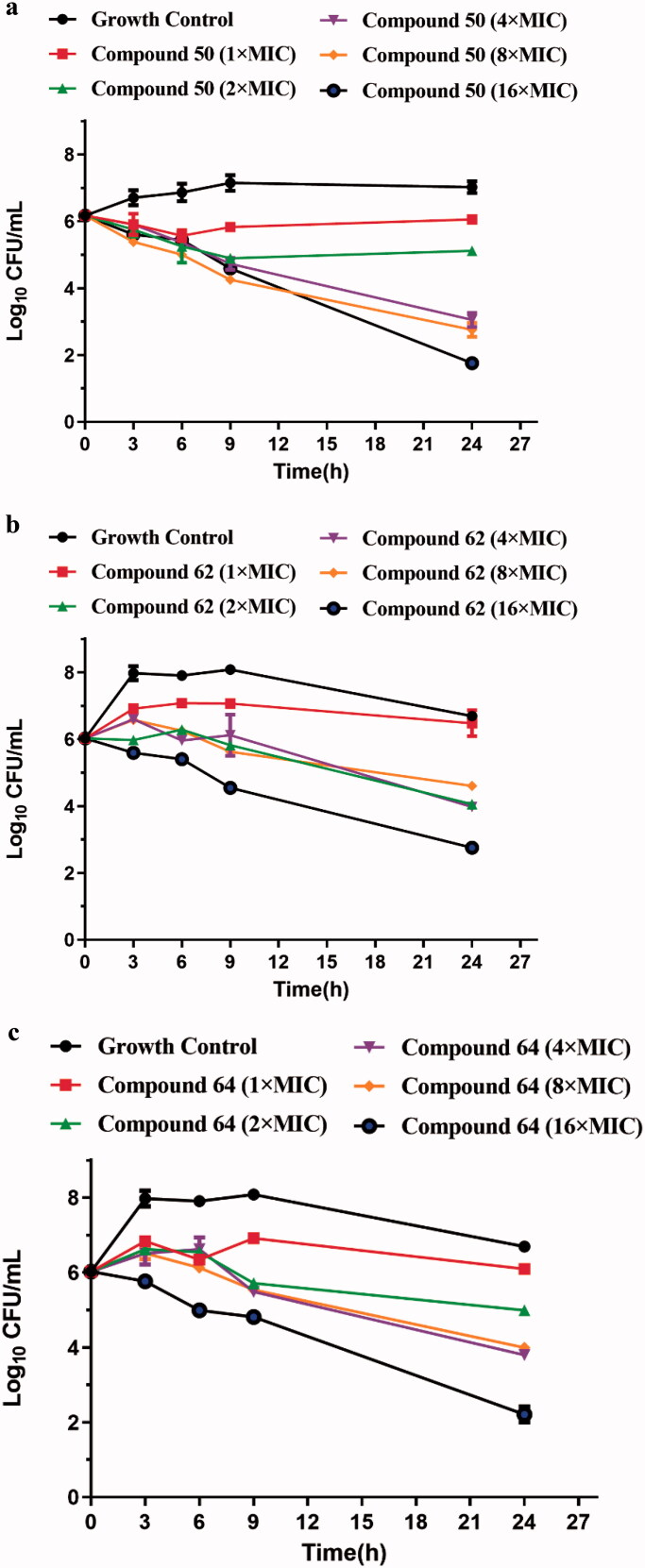
Time-kill curves for MRSA ATCC 43300 with different concentrations of compound **50** (a), compound **62** (b) and compound **64** (c).

The time-kill kinetic approach was used to investigate the anti-MRSA activity of compounds **50**, **62** and **64**. The experimental results were presented in graphic form in Figure 2. Compared with growth control, compounds **50** and **62** inhibited the reproduction of bacteria to a certain extent. When their concentrations reached 8 × MIC and cultured for 24 h, they showed relatively anti-MRSA bacteriostatic kinetics. Compound **64** at 16 × MIC induced relatively MRSA killing (2.21 log_10_ CFU/mL reduction) after 3 h incubation, and marked bacterial growth inhibition was observed at 24 h (4.49 log_10_ CFU/mL reduction). In conclusion, these data also demonstrated that compounds **50**, **62** and **64** induced dose- and time-dependent growth inhibition against MRSA, with a bactericidal effect at the concentrations higher than 4 × MIC.

### 2.3. Surface plasmon resonance (SPR) binding studies

**Figure 3.**
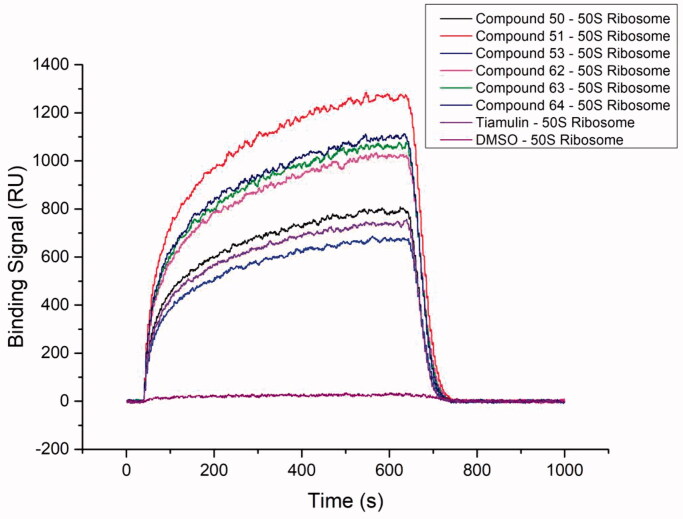
SPR binding signal curve of the selected compounds and controls to *50S* ribosome.

In order to gain some insight into the binding mode of the potent inhibitors, SPR experiments were performed. Since compounds **50**, **51**, **53**, **62**, **63**, and **64** showed more potent antibacterial activity than pleuromutilin *in vitro*, these compounds were further studied by SPR. The response unit (RU) of surface resonance was compared to determine the different binding affinities between each compound and the *50S* ribosome. The association rate constants (K_a_) and dissociation rate constant (K_d_) represent the rate of association and dissociation in the binding reaction, respectively. The equilibrium dissociation constant (K_D_) indicated the degree of dissociation of compounds and the *50S* ribosome in the equilibrium state^29^. The K_a_, K_d_ and K_D_ of the selected compounds and controls were shown in Table 3. Their binding curves during the experiment were shown in Figure 3, as well as the concentration gradient curves were shown in Figure SI **27–34**.

**Table 3.** Affinity constants of pleuromutilin derivatives and controls.

Compound No.	Protein Name	K_a_ (1/Ms)^a^	K_d_ (1/s)^b^	K_D_ (M)^c^
50	*50S* Ribosome	1.70 × 10^2^	3.93 × 10^−6^	2.32 × 10^−8^
51	*50S* Ribosome	3.80 × 10^3^	3.29 × 10^−2^	8.66 × 10^−6^
53	*50S* Ribosome	7.89 × 10	4.03 × 10^−3^	5.10 × 10^−5^
62	*50S* Ribosome	7.34 × 10^2^	1.30 × 10^−2^	1.77 × 10^−5^
63	*50S* Ribosome	9.92 × 10^2^	3.45 × 10^−3^	3.48 × 10^−6^
64	*50S* Ribosome	1.20 × 10^3^	7.64 × 10^−4^	6.36 × 10^−7^
Tiamulin	*50S* Ribosome	1.22 × 10^2^	4.44 × 10^−6^	3.63 × 10^−8^
DMSO	*50S* Ribosome	1.19	6.83 × 10^−1^	5.75 × 10^−1^

All values are represented as the mean ± SD: ^a^K_a_ represents the ratio of binding complex formed by combination in unit time to the initial binding complex before dissociation; ^b^K_d_ represents the ratio of dissociated binding complex in unit time to the initial binding complex before dissociation; ^c^K_D_ represents the dissociation degree of the binding complex at equilibrium, K_D_ = K_d_/K_a_.

According to the affinity measurement, the selected compounds all showed certain affinities for *50S* ribosome. The surface of the chip regenerated well, indicating that there was no irreversible binding between the tested compound and *50S* ribosome, which was in line with the Langmuir binding model. Among all the compounds to be tested, compounds **50**, **51**, **53**, **63**, **64** and tiamulin showed strong interaction intensity level (10^−8 ^M < K_D_ <10^−5 ^M) for *50S* ribosome, and compound **62** showed middle affinity (10^−5 ^M < K_D_ < 10^−3 ^M)^30^. Compounds **51**, **53**, **62**, **63** have higher K_d_ values, which means faster dissociation rates and would presumably be difficult to utilise *in vivo*^30^. The *50S* ribosome was integrated with these derivatives in a dose-dependent manner (Figure SI **27–33**), while the combination with DMSO was not obvious (Figure SI **34**). The result suggested that these derivatives were effective in interacting with 5*0S* ribosome through reversible binding. In addition, compound **50** showed the strongest interaction intensity with the macromolecule, followed by compound **64**.

### 2.4. Molecular docking study

**Figure 4.**
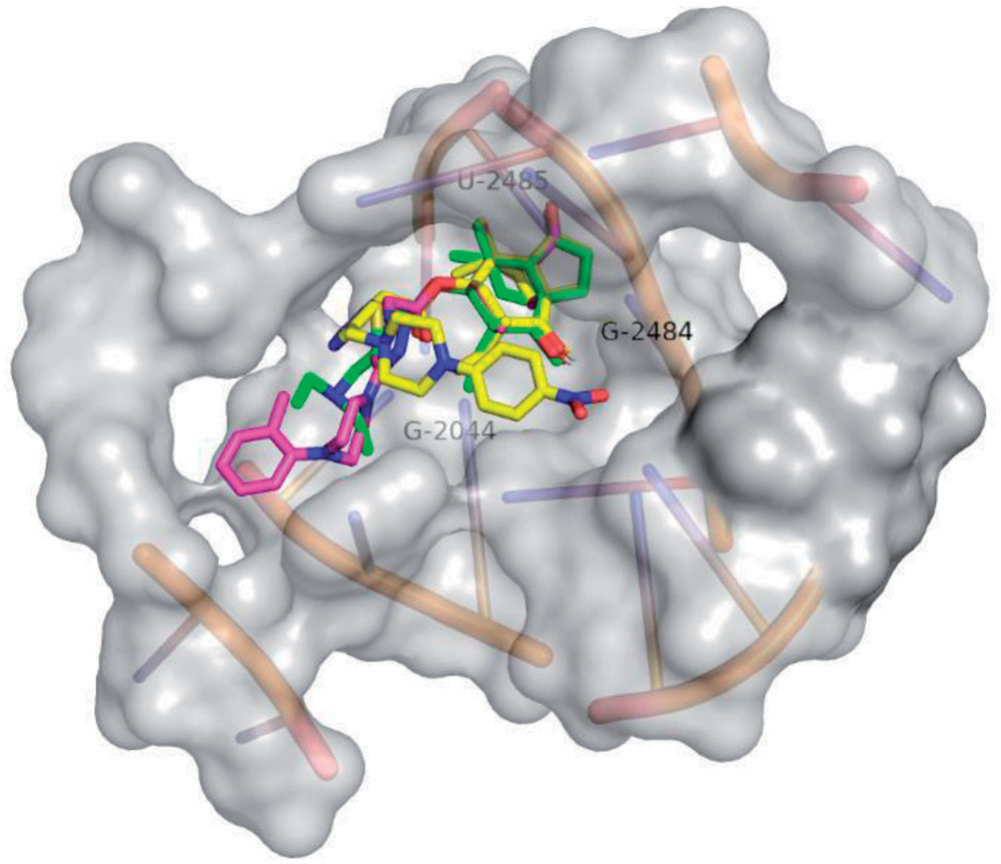
The superposition of the best compound conformation of tiamulin (green) and compound **50** (magenta) and compound **64** (yellow) docked to the binding pocket of *50S* ribosome (1XBP).

To further study the binding of compounds with ribosome, a series of docking simulations were conducted to compare the binding modes of **50** and **64**. The results for the two compounds and tiamulin (Figure SI **35**) present a similar binding mode, the superposition of the three docked compounds were presented in Figure 4.

**Figure 5.**
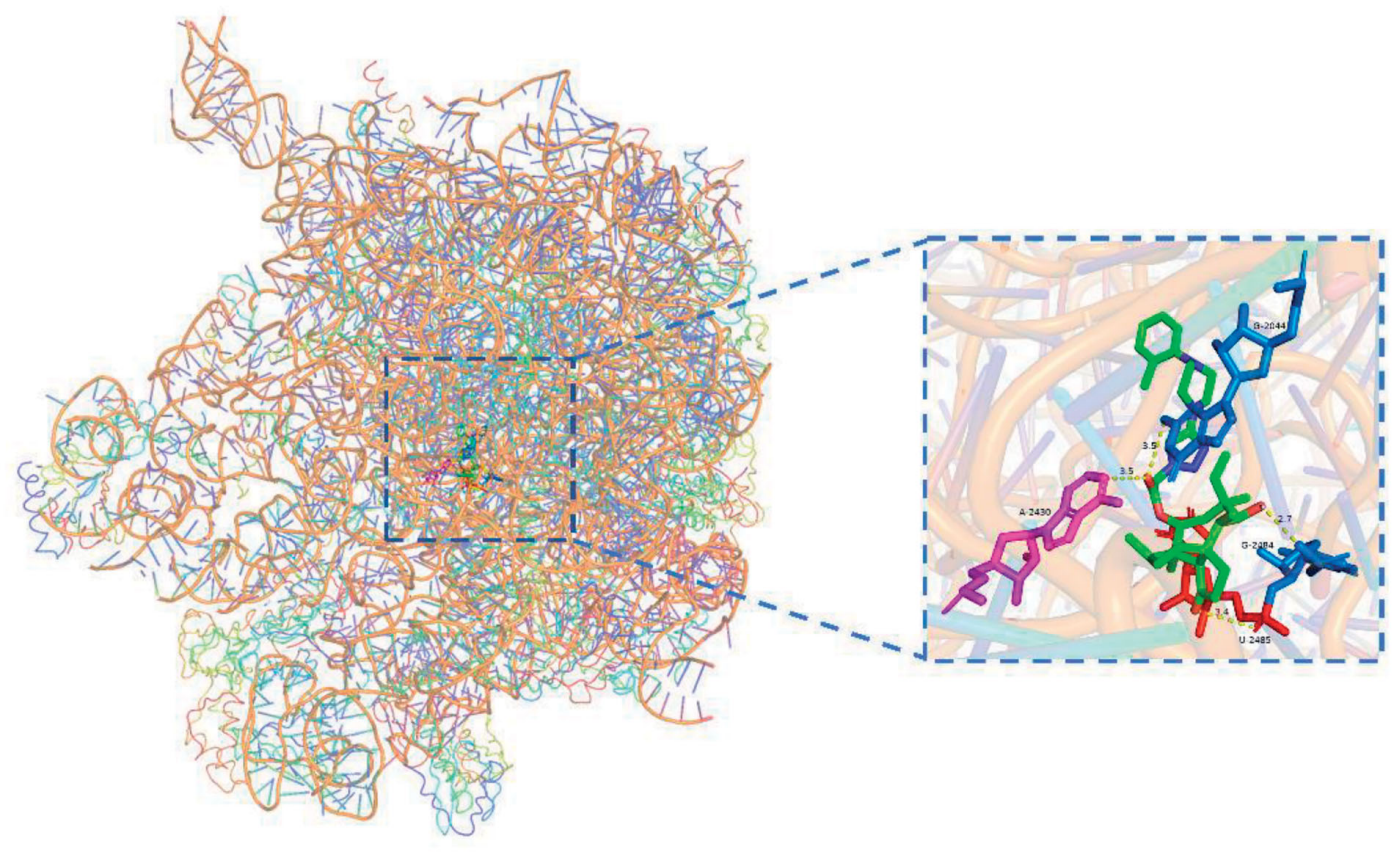
Docking mode of compound **50** (green) to *50S* ribosome (1XBP).

**Figure 6.**
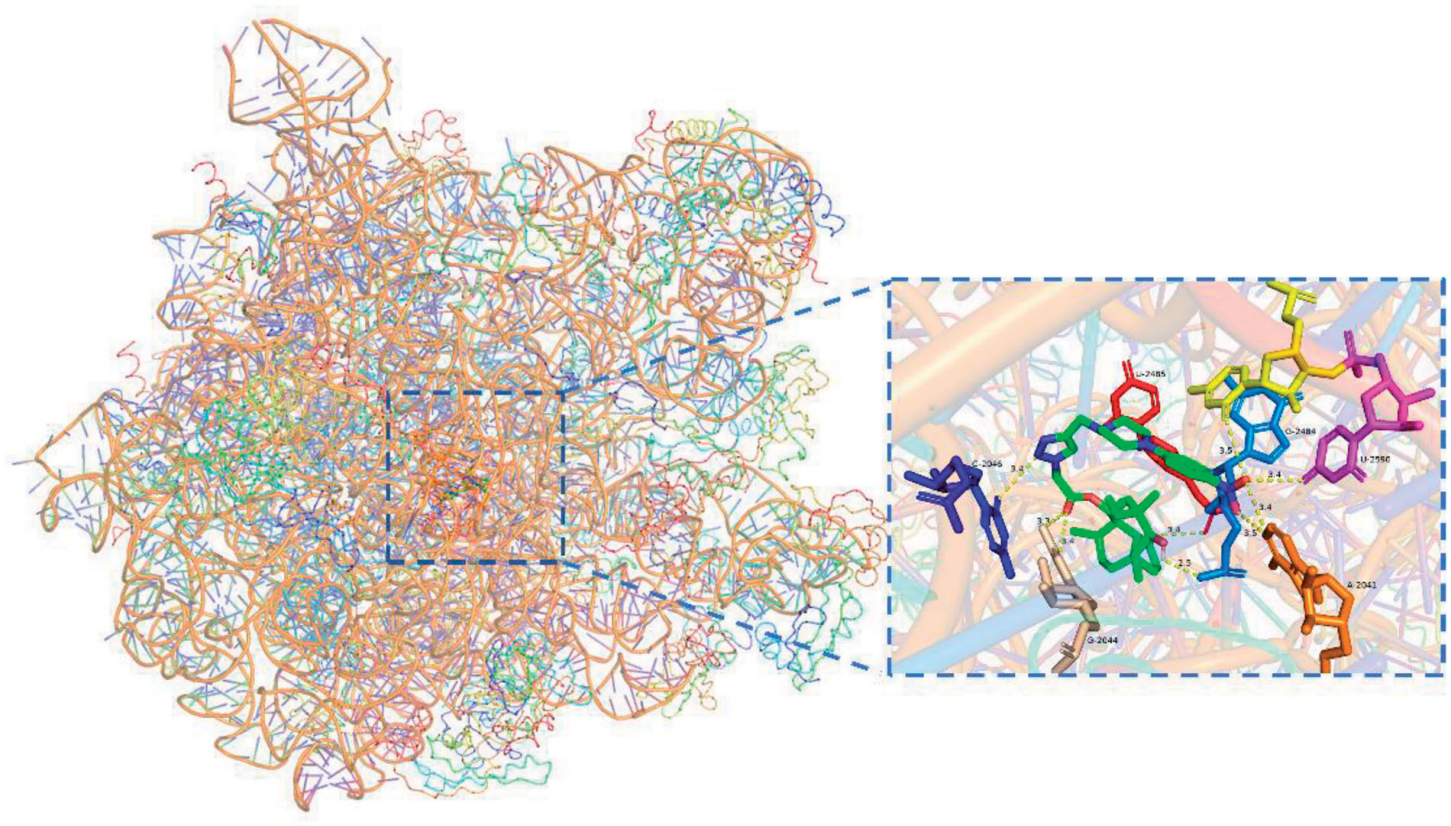
Docking mode of compound **64** (green) to *50S* ribosome (1XBP).

Upon semi-flexible docking into the *50S* ribosomal subunit (PDB ID code: 1XBP), the binding free energy (ΔG_b_) of compound **50** (RMSD = 0.736 Å) was calculated to be −12.0 kcal/mol. The ΔG_b_ of compound **64** (RMSD = 1.529 Å) was calculated to be −11.1 kcal/mol. Docking mode of compounds 50 and 64 to *50S* ribosome (1XBP) were presented in Figures 5 and 6. For each docking models, at least three strong hydrogen bonds were formed through the interaction of the core structure of the ligand and the macromolecule, namely the hydroxyl group of the eight-membered ring and the residue of G-2484, the carbonyl group of the five-membered ring and the residue of U-2485, the carbonyl group of the side chain C-21 and the residue of G-2044 (Table 4).

**Table 4.** Binding free energy and number of noncovalent molecular interactions

Compound No.	ΔG_b_ (kcal/mol)	Noncovalent Molecular Interaction			
		Hydro I Interaction	Atom of Compound	Residue	Distance (Å)
50	−12.0	H-bonding	C=O (ester)	G-2044	3.5
		H-bonding	C=O (ester)	A-2430	3.5
		H-bonding	OH (eight-membered ring)	G-2484	2.7
		H-bonding	C=O (five-membered ring)	U-2485	3.4
64	−11.1	H-bonding	NO_2_	A-2041	3.4
		H-bonding	NO_2_	A-2041	3.5
		H-bonding	C=O (ester)	G-2044	3.3
		H-bonding	C=O (ester)	G-2044	3.4
		H-bonding	Triazole	C-2046	3.4
		H-bonding	OH (eight-membered ring)	G-2484	2.5
		H-bonding	C=O (five-membered ring)	U-2485	3.4
		H-bonding	NO_2_	C-2589	3.5
		H-bonding	NO_2_	U-2590	3.4
Tiamulin	−8.5	H-bonding	C=O (ester)	G-2044	3.3
		H-bonding	C=O (ester)	G-2044	3.4
		H-bonding	C=O (ester)	A-2430	3.3
		H-bonding	OH (eight-membered ring)	G-2484	3.3
		H-bonding	C=O (five-membered ring)	C-2431	3.3
		H-bonding	C=O (five-membered ring)	U-2485	3.8
		H-bonding	N (diethylamino group)	C-2046	3.2

The nine hydrogen bonds which were formed between compound **64** and *50S* ribosome (1XBP) revealed why compound **64** was the most active agents. When compound **50** was docked, it displayed four hydrogen bonds. Compound 50 displayed a docking model similar to that of tiamulin, with an RMSD of 0.736 Å. This may explain that compound **50** and tiamulin have a similar affinity for *50S* ribosome in SPR experiments, namely, they have similar K_D_ values (2.32 × 10^−8 ^M VS 3.63 × 10^−8 ^M). The superior docking modes of compound **50** indicated that it might has a stronger affinity with *50S* ribosome.

### 2.5. Cytotoxicity

**Figure 7.**
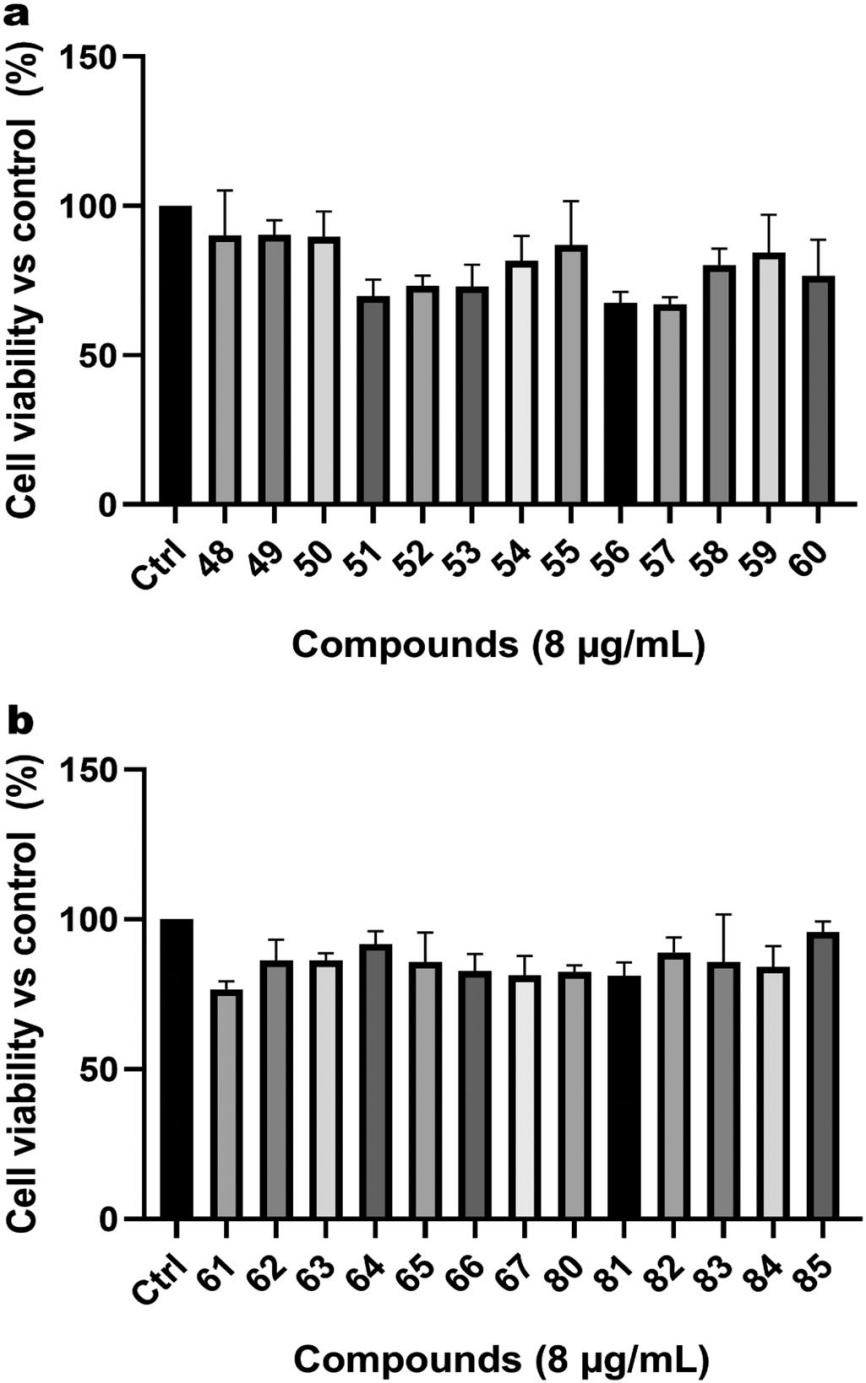
The cytotoxicity assay of pleuromutilin derivatives to RAW 264.7 cells at the concentration of 8 μg/mL.

The presence of compounds can affect cellular basic physiological processes, inhibited proliferation, even reduce cell survival, etc. Macrophage generally exhibited microbicidal activity and was an important line of defense against invading microorganism. Therefore, cytotoxicity of the pleuromutilin derivatives to RAW 264.7 cells was evaluated by MTT assay. The results were shown in Figure 7. By and large, most of these compounds did not affect the viability of RAW 264.7 cells at the concentration of 8 µg/mL, which was an acceptable starting point for further drug clinical trials.

## 3. Conclusions

Novel series of pleuromutilin derivatives possessing 1,2,3-triazole moieties were synthesised via click reactions under mild conditions. These derivatives were first evaluated for their *in vitro* antibacterial activity against MRSA (ATCC 43300), *S. aureus* (ATCC 29213), *S. aureus* (AD3), and *S. aureus* (144). The obtained MIC and MBC values revealed that all the synthesised derivatives showed potent antibacterial activity. Among the derivatives prepared, compounds **50**, **62** and **64** were the most active antibacterial agents against MRSA. In the following time-kill kinetic approach, compounds **50**, **62** and **64** showed relatively bacteriostatic kinetics against MRSA. According to the SPR affinity measurement, all the selected compounds (**50**, **51**, **53**, **63**, **64**) performed an obvious affinity to *50S* ribosome, and the binding mode between these compounds and *50S* ribosome was reversible. Two compounds (**50** and **64**) with good antibacterial activity and high affinity with *50S* ribosome have been further analysed by molecular docking. The results showed that the binding free energies were −12.0 kcal/mol and −11.1 kcal/mol, respectively. SPR and molecular docking study suggest these pleuromutilin derivatives bind within the *50S* subunit of ribosome, thereby interfering with bacterial protein synthesis and exerting antibacterial activity. The current research results indicated that the promising pleuromutilin derivatives **50** might serve as a possible lead compound for further optimisation and discovery of new antibiotics.

## 4. Experimental

### 4.1. Materials

Pleuromutilin (>90% pure) was purchased from Great Enjoyhood Biochemical Co. Ltd., (Sichuan, China). Unless noted otherwise, all reagents and solvents were purchased from commercial suppliers as reagent grade and were used as supplied. Purification of all compounds by column chromatography was carried out using silica gel (200–300 mesh, Branch of Qingdao Haiyang Chemical Co. Ltd., Shandong, China). ^1^H-NMR and ^13 ^C-NMR spectra were recorded at Bruker AV-400 or AV-600 spectrometer. The chemical shift values (*δ*) are reported in ppm relative to tetramethylsilane as internal standard and the coupling constant (*J*) is in Hertz. High-resolution mass spectra were conducted using Waters Acquity UPLC-LCT Premier XE with an electro spray ionisation (ESI) source or Thermo Scientific^TM^ Q Exactive Focus LC-MS.

### 4.2. Synthesis

A general synthesis strategy based on compound 22-*O*-tosylpheuromutilin (compound **7**) and a variety of piperazine derivatives or secondary amines were used (Schemes 1 and 2).

#### 4.2.1. 22-*O*-Tosylpleuromutilin (6)

A solution of pleuromutilin **1** (1.0 g, 2.64 mmol) in ethyl acetate (10.0 ml) was stirred in a round bottom flask, and *p*-toluenesulfonyl chloride (0.55 g, 2.90 mmol) was added. Sodium hydroxide (0.21 g, 5.28 mmol) was dissolved in 5 ml of water and dropped into the aforementioned solution with the whole dropping time of about 0.5 h. The mixture was then stirred at room temperature for 3 h, TLC analysis (ethyl acetate/dichloromethane/petroleum ether = 1:1:2) indicated complete consumption of starting material. Then 10 ml of water was added to the solution and the resulting mixture continue stirring for 0.5 h. Then the was poured into the separation funnel and extract with 10 ml CHCl_3_ for three times. The organic phase was combined and washed with brine and water. Then the organic phase was dried over anhydrous Na_2_SO_4_, filtered, and concentrated *in vacuo* to give a residue. The residue was precipitated from isopropanol to give a white solid (1.20 g, 85.7%).

#### 4.2.2. 22-*O*-Azidoacetate-deoxypleuromutilin (7)

Compound **6** (1 g, 1.87 mmol) was dissolved in acetone (20 ml), to which a solution of sodium azide (0.36 g, 5.61 mmol) in deionised water (5 ml) was added. The reaction was mixed in a round bottom flask and refluxed for 4 h. After completion of the reaction, the reaction solution was poured into a separating funnel, extracted twice with 10 ml CHCl_3_, and washed twice with a saturated aqueous sodium chloride solution. The organic phase was dried over anhydrous sodium sulphate and concentrated under reduced pressure to give the crude product. The crude product was purified by silica gel column chromatography using petroleum ether/ethyl acetate (v: v = 2:1) as eluent to obtain compound **7** (white solid, 71.3% yield).

#### 4.2.3. General synthesis method of piperazine derivatives containing propynyl group (28–27)

Piperazine derivatives (13.67 mmol) was dissolved in DCM (30 ml), potassium carbonate (3.78 g, 27.34 mmol) was added. The reaction was cooled in an ice bath and 3-bromopropyne (1.63 g, 13.67 mmol) was added dropwise. The reaction was performed at room temperature overnight. After the reaction was completed, 20 ml water was added, the reaction solution was continuously stirred for 0.5 h and poured into a separating funnel, extracted twice with 20 ml chloroform. The organic phase was washed twice with brine, then dried over Na_2_SO_4_ and concentrated under reduced pressure to obtain a crude product. The crude product was purified by column chromatography to obtain compounds **28–47**, respectively.

#### 4.2.4. 22-(2-(4-(4-Methyl-piperazin-1-yl)-methyl)-1H-1,2,3-triazol-1-yl)-22-deoxy pleuromutilin (48)

Compound **7** (0.69 g, 1.70 mmol) and 1-methyl-4-(prop-2-yn-1-yl) piperazine (**28**) (0.19 g, 1.70 mmol) were added in a mixture solution of *t*-butanol (10 ml) and of water (10 ml), and copper sulphate pentahydrate (0.0033 g, 0.068 mmol) and sodium ascorbate (0.0013 g, 0.068 mmol), and the reaction was stirred at room temperature for 3 h. After the reaction was completed, the reaction solution was poured into a separating funnel, 40 ml of ethyl acetate was added for extraction, and the organic phases were combined. After TLC analysis displayed that the reaction was complete, the reaction solution was poured into a separatory funnel, and 40 ml of ethyl acetate was added for extraction. The organic phase was washed twice with saturated aqueous sodium chloride solution, dried over Na_2_SO_4_, and concentrated under reduced pressure to obtain crude product. The crude product was purified by silica gel column chromatography using petroleum ether/ethyl acetate (v: v = 4:1) as eluent to obtain the product compound **48**. White powder; yield: 48%; ^1^H NMR (600 MHz, DMSO-d_6_) *δ* 7.93 (s, 1H), 6.12 (dd, *J* = 17.8, 11.2 Hz, 1H), 5.76 (s, 1H), 5.56 (d, *J* = 8.3 Hz, 1H), 5.33**–**5.22 (m, 2H), 5.13**–**5.02 (m, 2H), 4.54 (d, *J* = 6.1 Hz, 1H), 3.54 (s, 1H), 3.41 (t, *J* = 6.1 Hz, 1H), 3.32 (s, 4H), 2.50 (p, *J* = 1.8 Hz, 4H), 2.42**–**2.37 (m, 1H), 2.14 (s, 4H), 2.11**–**2.01 (m, 3H), 1.69**–**1.57 (m, 2H), 1.46 (dtd, *J* = 14.1, 7.1, 3.4 Hz, 1H), 1.39**–**1.20 (m, 5H), 1.18 (s, 3H), 1.06 (s, 3H), 0.81 (d, *J* = 7.0 Hz, 3H), 0.60 (d, *J* = 7.1 Hz, 3H). ^13 ^C NMR (151 MHz, DMSO) *δ* 217.51, 166.05, 144.05, 141.19, 125.56, 115.86, 72.96, 70.87, 57.58, 55.38, 55.08, 52.89, 52.61, 51.50, 46.15, 45.39, 44.60, 43.72, 41.95, 40.53, 36.93, 36.69, 34.45, 30.52, 29.06, 27.01, 24.92, 16.55, 14.66, 12.01. HR-MS (ESI): Calcd for C_30_H_47_N_5_O_4_(M + H^+^): 542.3628; Found: 542.3715.

#### 4.2.5. 22-(2-(4-(4-Phenyl-piperazin-1-yl)-methyl)-1H-1,2,3-triazol-1-yl)-22-deoxy pleuromutilin (49)

Compound **49** was prepared from compound **7** and 1-phenyl-4-(prop-2-yn-1-yl) piperazine (**29)** in the same procedure as described for compound **48**. White powder; yield: 57%; ^1^H NMR (600 MHz, DMSO-d_6_) *δ* 8.00 (s, 1H), 7.23**–**7.17 (m, 2H), 6.93**–**6.88 (m, 2H), 6.76 (tt, *J* = 7.2, 1.0 Hz, 1H), 6.12 (dd, *J* = 17.8, 11.2 Hz, 1H), 5.57 (d, *J* = 8.4 Hz, 1H), 5.37**–**5.23 (m, 2H), 5.16**–**5.01 (m, 2H), 4.55 (d, *J* = 6.1 Hz, 1H), 3.64 (d, *J* = 14.7 Hz, 2H), 3.43**–**3.37 (m, 1H), 3.32 (s, 2H), 3.11 (s, 4H), 2.50 (p, *J* = 1.8 Hz, 3H), 2.38 (t, *J* = 1.8 Hz, 1H), 2.21**–**2.13 (m, 1H), 2.11**–**1.95 (m, 3H), 1.69**–**1.53 (m, 2H), 1.46 (dtd, *J* = 14.1, 7.1, 3.4 Hz, 1H), 1.40**–**1.23 (m, 4H), 1.19 (s, 3H), 1.06 (s, 3H), 0.81 (d, *J* = 7.0 Hz, 3H), 0.62 (d, *J* = 7.1 Hz, 3H). ^13 ^C NMR (151 MHz, DMSO) *δ* 217.49, 166.03, 151.48, 141.18, 129.34, 125.79, 125.75, 119.24, 115.88, 115.82, 72.96, 70.89, 60.22, 57.58, 52.49, 51.58, 48.67, 46.23, 45.38, 44.61, 43.71, 43.03, 41.96, 40.54, 36.93, 36.69, 34.45, 30.52, 29.08, 27.01, 24.91, 21.24, 16.56, 14.68, 12.01. HR-MS (ESI): Calcd for C_35_H_49_N_5_O_4_(M + H^+^): 604.3784; Found: 604.3824.

#### 4.2.6. 22-(2-(4-(4-(*o*-Tolyl)-piperazin-1-yl)-methyl)-1H-1,2,3-triazol -1-yl)-22-deoxy pleuromutilin (50)

Compound **50** was prepared from compound **7** and 1-(prop-2-yn-1-yl)-4-(*o*-tolyl) piperazine (**30)** in the same procedure as described for compound **48**. White powder; yield: 58%; ^1^H NMR (600 MHz, DMSO-d_6_) *δ* 8.00 (s, 1H), 7.13 (ddd, *J* = 14.7, 7.3, 1.6 Hz, 1H), 6.99 (dd, *J* = 8.0, 1.2 Hz, 1H), 6.94 (td, *J* = 7.4, 1.2 Hz, 1H), 6.14 (ddd, *J* = 20.0, 17.8, 11.2 Hz, 1H), 5.75 (s, 1H), 5.61 (dd, *J* = 41.0, 8.4 Hz, 1H), 5.37**–**5.23 (m, 1H), 5.14**–**5.03 (m, 2H), 4.55 (dd, *J* = 7.3, 6.1 Hz, 1H), 4.10**–**3.97 (m, 1H), 3.66 (s, 1H), 3.42 (dt, *J* = 18.3, 6.1 Hz, 1H), 3.32 (s, 1H), 2.88**–**2.71 (m, 3H), 2.68**–**2.53 (m, 2H), 2.50 (p, *J* = 1.8 Hz, 3H), 2.48**–**2.41 (m, 1H), 2.40**–**2.27 (m, 1H), 2.22 (s, 2H), 2.20**–**2.00 (m, 4H), 1.70**–**1.66 (m, 1H), 1.64**–**1.59 (m, 1H), 1.58**–**1.40 (m, 1H), 1.38 (s, 2H), 1.35**–**1.21 (m, 3H), 1.19 (s, 2H), 1.07 (d, *J* = 6.4 Hz, 3H), 1.04**–**0.94 (m, 1H), 0.82 (dd, *J* = 17.0, 7.0 Hz, 3H), 0.62 (t, *J* = 7.6 Hz, 3H). ^13 ^C NMR (151 MHz, DMSO) *δ* 217.56, 167.74, 166.02, 141.27, 132.20, 131.26, 126.97, 119.16, 115.87, 72.96, 70.89, 70.66, 57.66, 55.38, 50.80, 45.43, 44.62, 43.91, 43.73, 41.99, 36.98, 36.75, 36.69, 34.46, 34.44, 30.57, 30.52, 29.06, 27.03, 24.96, 18.03, 16.57, 16.46, 14.95, 14.69, 12.01. HR-MS (ESI): Calcd for C_36_H_51_N_5_O_4_(M + H^+^): 618.3941; Found: 618.4003.

#### 4.2.7. 22-(2-(4-(4-(*m*-Tolyl)-piperazin-1-yl)-methyl)-1H-1,2,3-triazol-1-yl)-22-deoxy pleuromutilin (51)

Compound **51** was prepared from compound **7** and 1-(prop-2-yn-1-yl)-4-(*m*-tolyl) piperazine (**31)** in the same procedure as described for compound **48**. White powder; yield: 52%; ^1^H NMR (600 MHz, Chloroform-*d*) *δ* 7.65 (s, 1H), 7.15 (t, *J* = 7.7 Hz, 1H), 6.74 (d, *J* = 11.8 Hz, 2H), 6.69 (d, *J* = 7.4 Hz, 1H), 6.41 (dd, *J* = 11.0, 17.4 Hz, 1H), 5.84 (d, *J* = 8.5 Hz, 1H), 5.38**–**5.30 (m, 1H), 5.23 (dd, *J* = 1.4, 17.4 Hz, 1H), 5.12 (d, *J* = 17.3 Hz, 1H), 5.04 (d, *J* = 17.5 Hz, 1H), 3.79 (s, 2H), 3.37 (dd, *J* = 6.5, 10.6 Hz, 1H), 3.28 (s, 4H), 2.73 (s, 4H), 2.35**–**2.16 (m, 6H), 2.10 (s, 1H), 1.78 (dd, *J* = 3.2, 14.4 Hz, 1H), 1.57**–**1.39 (m, 3H), 1.37 (s, 3H), 1.36**–**1.28 (m, 2H), 1.27 (s, 2H), 1.20 (s, 3H), 1.15 (td, *J* = 4.5, 14.2 Hz, 1H), 0.89 (d, *J* = 7.0 Hz, 3H), 0.73 (d, *J* = 7.1 Hz, 3H). ^13 ^C NMR (151 MHz, CDCl_3_) *δ* 216.44, 166.05, 162.08, 156.26, 150.57, 138.75, 128.91, 128.91, 120.70, 120.69, 113.30, 96.31, 90.59, 77.21, 76.99, 76.78, 74.54, 73.41, 71.05, 57.98, 45.39, 44.74, 44.00, 41.85, 36.53, 36.06, 34.36, 30.33, 29.69, 29.22, 26.78, 26.38, 24.80, 21.74, 16.80, 14.65, 11.44. HR-MS (ESI): Calcd for C_36_H_51_N_5_O_4_(M + H^+^): 618.3941; Found: 618.3901.

#### 4.2.8. 22-(2-(4-(4-(*p*-Tolyl)-piperazin-1-yl)-methyl)-1H-1,2,3-triazol-1-yl)-22-deoxy pleuromutilin (52)

Compound **52** was prepared from compound **7** and 1-(prop-2-yn-1-yl)-4-(*p*-tolyl) piperazine (**32)** in the same procedure as described for compound **48**. White powder; yield: 43%; ^1^H NMR (600 MHz, Chloroform-*d*) *δ* 7.65 (s, 1H), 7.08 (d, *J* = 8.2 Hz, 2H), 6.85 (d, *J* = 8.1 Hz, 2H), 6.41 (dd, *J* = 11.0, 17.4 Hz, 1H), 5.84 (d, *J* = 8.6 Hz, 1H), 5.40**–**5.31 (m, 2H), 5.25**–**5.20 (m, 1H), 5.14**–**5.03 (m, 2H), 3.79 (s, 2H), 3.36 (dd, *J* = 6.5, 10.7 Hz, 1H), 3.22 (s, 4H), 2.73 (s, 4H), 2.39**–**2.29 (m, 2H), 2.28 (s, 3H), 2.26**–**2.18 (m, 1H), 2.09 (s, 2H), 1.78 (d, *J* = 14.3 Hz, 1H), 1.72**–**1.62 (m, 2H), 1.37 (s, 3H), 1.27 (d, *J* = 3.0 Hz, 5H), 1.19 (s, 3H), 0.89 (d, *J* = 5.5 Hz, 3H), 0.73 (d, *J* = 7.1 Hz, 3H). ^13 ^C NMR (151 MHz, DMSO) *δ* 165.88, 141.21, 130.39, 129.79, 128.08, 116.14, 115.88, 100.65, 78.40, 73.00, 70.94, 62.76, 57.60, 51.92, 45.39, 44.62, 43.76, 41.97, 40.60, 36.93, 36.91, 36.69, 35.88, 34.45, 30.52, 30.32, 29.07, 27.01, 24.91, 20.49, 16.55, 14.69, 14.64, 11.99. HR-MS (ESI): Calcd for C_36_H_51_N_5_O_4_(M + H^+^): 618.3941; Found: 618.3959.

#### 4.2.9. 22-(2-(4-(4-(2-Fluorophenyl)-piperazin-1-yl)-methyl)-1H-1,2,3-triazol-1-yl)-22-deoxy pleuromutilin (53)

Compound **53** was prepared from compound **7** and 1-(2-fluorophenyl)-4-(prop-2-yn-1-yl)piperazine (**33)** in the same procedure as described for compound **48**. White powder; yield: 53%; ^1^H NMR (600 MHz, DMSO-d_6_) *δ* 8.03 (s, 1H), 7.14**–**7.05 (m, 2H), 7.03**–**6.98 (m, 1H), 6.95 (qd, *J* = 6.5, 5.6, 2.4 Hz, 1H), 6.13 (dd, *J* = 17.8, 11.2 Hz, 1H), 5.76 (s, 1H), 5.57 (d, *J* = 8.4 Hz, 1H), 5.38**–**5.24 (m, 2H), 5.18**–**5.02 (m, 2H), 4.55 (d, *J* = 6.1 Hz, 1H), 3.41 (t, *J* = 6.1 Hz, 1H), 3.32 (s, 1H), 3.00 (s, 4H), 2.50 (p, *J* = 1.9 Hz, 3H), 2.42**–**2.34 (m, 1H), 2.23**–**2.13 (m, 1H), 2.11**–**1.98 (m, 3H), 1.71**–**1.57 (m, 2H), 1.47 (ddd, *J* = 11.2, 6.9, 3.4 Hz, 1H), 1.45**–**1.16 (m, 8H), 1.06 (s, 3H), 0.99 (td, *J* = 14.0, 4.4 Hz, 1H), 0.81 (d, *J* = 7.0 Hz, 3H), 0.62 (d, *J* = 7.1 Hz, 3H). ^13 ^C NMR (151 MHz, DMSO) *δ* 217.47, 165.97, 156.20, 154.58, 141.18, 125.26, 125.23, 122.75, 122.70, 119.64, 116.43, 116.29, 115.87, 72.97, 70.90, 57.57, 55.38, 51.50, 50.46, 45.38, 44.60, 43.72, 41.96, 40.55, 36.92, 36.69, 34.44, 30.51, 29.06, 27.01, 24.91, 21.96, 16.56, 14.67, 12.01. HR-MS (ESI): Calcd for C_35_H_48_FN_5_O_4_(M + H^+^): 622.3690; Found: 622.3629.

#### 4.2.10. 22-(2-(4-(4-(3-Fluorophenyl)-piperazin-1-yl)-methyl)-1H-1,2,3-triazol-1-yl)-22-deoxy pleuromutilin (54)

Compound **54** was prepared from compound **7** and 1-(3-fluorophenyl)-4-(prop-2-yn-1-yl)piperazine (**34)** in the same procedure as described for compound **48**. White powder; yield: 52%; ^1^H NMR (600 MHz, Chloroform-*d*) *δ* 7.63 (s, 1H), 7.19 (td, *J* = 7.0, 8.3 Hz, 1H), 6.67 (dd, *J* = 2.3, 8.6 Hz, 1H), 6.62**–**6.50 (m, 2H), 6.42 (dd, *J* = 11.0, 17.4 Hz, 1H), 5.84 (d, *J* = 8.5 Hz, 1H), 5.38**–**5.30 (m, 1H), 5.23 (dd, *J* = 1.5, 17.4 Hz, 1H), 5.15**–**5.02 (m, 2H), 3.78 (s, 2H), 3.36 (dd, *J* = 6.5, 10.5 Hz, 1H), 3.24 (s, 4H), 2.70 (s, 4H), 2.34**–**2.16 (m, 3H), 2.14**–**2.06 (m, 2H), 1.78 (dd, *J* = 3.2, 14.6 Hz, 1H), 1.68 (ddd, *J* = 2.8, 5.9, 13.3 Hz, 1H), 1.46 (d, *J* = 10.5 Hz, 1H), 1.36 (s, 3H), 1.32 (d, *J* = 16.1 Hz, 4H), 1.28**–**1.26 (m, 1H), 1.19 (s, 3H), 1.14 (td, *J* = 4.4, 14.1 Hz, 1H), 0.89 (d, *J* = 7.0 Hz, 3H), 0.73 (d, *J* = 7.1 Hz, 3H). ^13 ^C NMR (151 MHz, CDCl_3_) *δ* 216.46, 164.98, 163.03, 138.55, 130.11, 130.05, 117.57, 111.16, 105.91, 102.76, 102.59, 77.21, 77.00, 76.79, 74.53, 71.00, 64.67, 64.63, 57.98, 48.54, 48.24, 45.39, 44.74, 44.00, 41.85, 36.53, 36.05, 34.36, 30.32, 26.78, 26.38, 24.80, 16.78, 14.65, 11.44. HR-MS (ESI): Calcd for C_35_H_48_FN_5_O_4_(M + H^+^): 622.3690; Found: 622.3621.

#### 4.2.11. 22-(2-(4-(4-(4-Fluorophenyl)-piperazin-1-yl)-methyl)-1H-1,2,3-triazol-1-yl)-22-deoxy pleuromutilin (55)

Compound **55** was prepared from compound **7** and 1-(4-fluorophenyl)-4-(prop-2-yn-1-yl)piperazine (**35)** in the same procedure as described for compound **48**. White powder; yield: 50%; ^1^H NMR (600 MHz, Chloroform-*d*) *δ* 7.63 (s, 1H), 7.09**–**6.99 (m, 2H), 6.99**–**6.91 (m, 2H), 6.42 (dd, *J* = 11.0, 17.4 Hz, 1H), 5.84 (d, *J* = 8.5 Hz, 1H), 5.38**–**5.30 (m, 1H), 5.23 (dd, *J* = 1.5, 17.4 Hz, 1H), 5.05 (d, *J* = 17.5 Hz, 1H), 3.80 (s, 2H), 3.36 (dd, *J* = 6.5, 10.6 Hz, 1H), 3.15 (s, 4H), 2.74 (s, 4H), 2.34**–**2.16 (m, 3H), 2.14**–**2.03 (m, 2H), 1.78 (dd, *J* = 3.2, 14.6 Hz, 1H), 1.73**–**1.63 (m, 1H), 1.58**–**1.38 (m, 4H), 1.36 (s, 3H), 1.35**–**1.25 (m, 2H), 1.19 (s, 3H), 1.14 (td, *J* = 4.4, 14.2 Hz, 1H), 0.89 (d, *J* = 7.0 Hz, 3H), 0.73 (d, *J* = 7.1 Hz, 3H). ^13 ^C NMR (151 MHz, CDCl_3_) *δ* 216.45, 168.59, 167.79, 165.01, 144.86, 140.06, 138.56, 124.44, 122.42, 119.02, 117.59, 116.00, 77.21, 76.99, 76.78, 74.54, 70.97, 57.98, 53.02, 51.61, 50.37, 44.75, 43.99, 41.85, 36.53, 36.05, 34.36, 30.33, 26.78, 26.36, 24.81, 16.78, 14.64, 11.44. HR-MS (ESI): Calcd for C_35_H_48_FN_5_O_4_(M + H^+^): 622.3690; Found: 622.3649.

#### 4.2.12. 22-(2-(4-(4-(2-Chlorophenyl)-piperazin-1-yl)-methyl)-1H-1,2,3-triazol-1-yl)-22-deoxy pleuromutilin (56)

Compound **56** was prepared from compound **7** and 1-(2-chlorophenyl)-4-(prop-2-yn-1-yl)piperazine (**36)** in the same procedure as described for compound **48**. White powder; yield: 36%; ^1^H NMR (600 MHz, Chloroform-*d*) *δ* 7.62 (s, 1H), 7.36 (dd, *J* = 1.5, 7.9 Hz, 1H), 7.22 (ddd, *J* = 1.6, 7.4, 8.1 Hz, 1H), 7.06 (dd, *J* = 1.5, 8.1 Hz, 1H), 6.98 (td, *J* = 1.5, 7.6 Hz, 1H), 6.42 (dd, *J* = 11.0, 17.4 Hz, 1H), 5.84 (d, *J* = 8.6 Hz, 1H), 5.38**–**5.30 (m, 1H), 5.23 (dd, *J* = 1.5, 17.4 Hz, 1H), 5.15**–**5.03 (m, 2H), 3.81 (s, 2H), 3.37 (dd, *J* = 6.5, 10.5 Hz, 1H), 3.11 (s, 4H), 2.74 (s, 4H), 2.34**–**2.16 (m, 3H), 2.10 (s, 1H), 1.78 (dd, *J* = 3.3, 14.5 Hz, 1H), 1.73**–**1.62 (m, 2H), 1.50**–**1.38 (m, 3H), 1.36 (s, 3H), 1.35**–**1.25 (m, 4H), 1.19 (s, 3H), 1.14 (td, *J* = 4.4, 14.1 Hz, 1H), 0.89 (d, *J* = 7.0 Hz, 3H), 0.73 (d, *J* = 7.1 Hz, 3H). ^13 ^C NMR (151 MHz, CDCl_3_) *δ* 216.46, 138.57, 132.63, 130.63, 128.80, 127.55, 123.66, 120.41, 117.60, 77.20, 76.99, 76.78, 75.69, 74.54, 70.93, 57.99, 54.62, 53.12, 51.59, 51.07, 45.39, 44.75, 43.99, 41.85, 36.54, 36.06, 34.36, 30.34, 26.78, 26.36, 24.81, 20.19, 16.78, 14.65, 11.44. HR-MS (ESI): Calcd for C_35_H_48_ClN_5_O_4_(M + H^+^): 638.3394; Found: 638.3401.

#### 4.2.13. 22-(2-(4-(4-(3-Chlorophenyl)-piperazin-1-yl)-methyl)-1H-1,2,3-triazol-1-yl)-22-deoxy pleuromutilin (57)

Compound **57** was prepared from compound **7** and 1-(3-chlorophenyl)-4-(prop-2-yn-1-yl)piperazine (**37)** in the same procedure as described for compound **48**. White powder; yield: 38%; ^1^H NMR (600 MHz, Chloroform-*d*) *δ* 7.63 (s, 1H), 7.16 (t, *J* = 8.1 Hz, 1H), 6.87 (t, *J* = 2.2 Hz, 1H), 6.84**–**6.76 (m, 2H), 6.42 (dd, *J* = 11.0, 17.4 Hz, 1H), 5.84 (d, *J* = 8.5 Hz, 1H), 5.38**–**5.30 (m, 1H), 5.23 (dd, *J* = 1.5, 17.4 Hz, 1H), 5.05 (d, *J* = 17.5 Hz, 1H), 3.78 (s, 2H), 3.37 (dd, *J* = 6.5, 10.5 Hz, 1H), 3.23 (s, 4H), 2.69 (s, 4H), 2.34**–**2.16 (m, 3H), 2.14**–**2.01 (m, 2H), 1.78 (dd, *J* = 3.3, 14.5 Hz, 1H), 1.73**–**1.62 (m, 2H), 1.57**–**1.39 (m, 3H), 1.36 (s, 3H), 1.35**–**1.25 (m, 2H), 1.19 (s, 3H), 1.15 (td, *J* = 4.4, 14.1 Hz, 1H), 0.89 (d, *J* = 7.0 Hz, 3H), 0.73 (d, *J* = 7.1 Hz, 3H). ^13 ^C NMR (151 MHz, CDCl_3_) *δ* 216.45, 192.88, 180.14, 167.59, 162.03, 138.56, 136.56, 134.94, 130.00, 117.58, 115.78, 113.92, 77.21, 76.99, 76.78, 74.53, 71.00, 57.98, 49.51, 45.39, 44.75, 44.00, 41.85, 36.52, 36.06, 34.36, 30.32, 26.78, 26.38, 24.80, 17.89, 16.78, 14.65, 11.44. HR-MS (ESI): Calcd for C_35_H_48_ClN_5_O_4_(M + H^+^): 638.3394; Found: 638.3301.

#### 4.2.14. 22-(2-(4-(4-(4-Chlorophenyl)-piperazin-1-yl)-methyl)-1H-1,2,3-triazol-1-yl)-22-deoxy pleuromutilin (58)

Compound **58** was prepared from compound **7** and 1-(4-chlorophenyl)-4-(prop-2-yn-1-yl)piperazine (**38)** in the same procedure as described for compound **48**. White powder; yield: 38%; ^1^H NMR (600 MHz, Chloroform-*d*) *δ* 7.79 (s, 1H), 7.21 (d, *J* = 8.4 Hz, 2H), 6.84 (d, *J* = 8.4 Hz, 2H), 6.41 (dd, *J* = 10.9, 17.4 Hz, 1H), 5.83 (d, *J* = 8.5 Hz, 1H), 5.38**–**5.30 (m, 1H), 5.26**–**5.17 (m, 1H), 5.12 (s, 1H), 3.89 (s, 2H), 3.36 (dd, *J* = 6.5, 10.4 Hz, 1H), 3.28 (s, 4H), 2.82 (s, 4H), 2.33**–**2.17 (m, 3H), 2.11 (d, *J* = 19.0 Hz, 2H), 1.78 (dd, *J* = 3.0, 14.9 Hz, 1H), 1.70**–**1.61 (m, 1H), 1.57**–**1.42 (m, 3H), 1.42**–**1.37 (m, 1H), 1.36**–**1.25 (m, 5H), 1.19 (s, 3H), 1.14 (td, *J* = 4.4, 14.1 Hz, 1H), 0.89 (d, *J* = 7.0 Hz, 3H), 0.72 (d, *J* = 7.0 Hz, 3H). ^13 ^C NMR (151 MHz, CDCl_3_) *δ* 216.45, 199.23, 180.90, 143.62, 138.48, 137.72, 135.31, 128.98, 117.53, 104.00, 90.95, 77.21, 77.00, 76.79, 74.54, 74.54, 71.08, 57.97, 45.39, 44.74, 44.01, 43.57, 41.85, 36.52, 36.05, 35.33, 34.36, 30.32, 26.78, 26.39, 24.80, 16.81, 14.66, 12.44, 11.44.HR-MS (ESI): Calcd for C_35_H_48_ClN_5_O_4_(M + H^+^): 638.3394; Found: 638.3452.

#### 4.2.15. 22-(2-(4-(4-(2-Methoxyphenyl)-piperazin-1-yl)-methyl)-1H-1,2,3-triazol-1-yl)-22-deoxy pleuromutilin (59)

Compound **59** was prepared from compound **7** and 1-(2-methoxyphenyl)-4-(prop-2-yn-1-yl)piperazine (**39)** in the same procedure as described for compound **48**. White powder; yield: 47%; ^1^H NMR (600 MHz, DMSO-d_6_) *δ* 8.00 (s, 1H), 6.96**–**6.90 (m, 2H), 6.88**–**6.82 (m, 2H), 6.13 (dd, *J* = 17.8, 11.2 Hz, 1H), 5.75 (s, 1H), 5.57 (d, *J* = 8.4 Hz, 1H), 5.38**–**5.23 (m, 2H), 5.15**–**5.03 (m, 2H), 4.54 (d, *J* = 6.1 Hz, 1H), 3.76 (s, 3H), 3.64 (s, 2H), 3.41 (t, *J* = 6.1 Hz, 1H), 3.32 (s, 3H), 2.95 (s, 4H), 2.51**–**2.50 (m, 3H), 2.43**–**2.36 (m, 1H), 2.23**–**2.13 (m, 1H), 2.11**–**1.98 (m, 3H), 1.68**–**1.55 (m, 2H), 1.47 (ddt, *J* = 11.8, 8.4, 4.2 Hz, 1H), 1.38**–**1.27 (m, 2H), 1.19 (s, 3H), 1.07 (s, 3H), 0.81 (d, *J* = 7.0 Hz, 3H), 0.62 (d, *J* = 7.1 Hz, 3H). ^13 ^C NMR (151 MHz, DMSO) *δ* 217.51, 166.04, 152.43, 141.67, 141.19, 122.83, 121.27, 118.36, 115.87, 112.37, 72.97, 70.91, 57.58, 55.77, 55.38, 52.95, 52.87, 51.59, 50.44, 45.39, 44.98, 44.60, 43.73, 41.96, 40.53, 36.93, 36.70, 34.44, 33.24, 30.52, 29.05, 27.01, 24.92, 16.57, 14.67, 12.01. HR-MS (ESI): Calcd for C_36_H_51_N_5_O_5_(M + H^+^): 634.3890; Found: 634.3895.

#### 4.2.16. 22-(2-(4-(4-(3-Methoxyphenyl)-piperazin-1-yl)-methyl)-1H-1,2,3-triazol-1-yl)-22-deoxy pleuromutilin (60)

Compound **60** was prepared from compound **7** and 1-(3-methoxyphenyl)-4-(prop-2-yn-1-yl)piperazine (**40)** in the same procedure as described for compound **48**. White powder; yield: 62%; ^1^H NMR (600 MHz, DMSO-d_6_) *δ* 8.04 (s, 1H), 7.09 (t, *J* = 8.1 Hz, 1H), 6.53**–**6.48 (m, 1H), 6.42 (t, *J* = 2.2 Hz, 1H), 6.12 (dd, *J* = 11.2, 17.8 Hz, 1H), 5.57 (d, *J* = 8.3 Hz, 1H), 5.35 (d, *J* = 17.6 Hz, 1H), 5.28 (d, *J* = 17.6 Hz, 1H), 5.08 (dd, *J* = 14.5, 32.5 Hz, 2H), 4.57 (d, *J* = 6.0 Hz, 1H), 3.70 (s, 3H), 3.41 (t, *J* = 6.2 Hz, 1H), 3.35 (d, *J* = 2.0 Hz, 6H), 3.13 (s, 4H), 2.50 (d, *J* = 2.0 Hz, 3H), 2.39 (s, 1H), 2.17 (dd, *J* = 11.0, 19.1 Hz, 1H), 2.12**–**1.98 (m, 3H), 1.63 (dd, *J* = 13.0, 25.7 Hz, 2H), 1.46 (h, *J* = 5.3, 6.3 Hz, 1H), 1.34 (t, *J* = 14.4 Hz, 2H), 1.30**–**1.22 (m, 3H), 1.19 (s, 3H), 1.07 (s, 3H), 0.99 (td, *J* = 4.4, 14.2 Hz, 1H), 0.81 (d, *J* = 6.9 Hz, 3H), 0.61 (d, *J* = 7.1 Hz, 3H). ^13 ^C NMR (151 MHz, DMSO) *δ* 217.47, 165.97, 160.66, 152.89, 141.19, 130.04, 125.88, 115.86, 108.53, 104.56, 101.98, 73.00, 70.97, 57.60, 55.33, 51.73, 48.55, 45.39, 44.62, 43.75, 41.97, 40.57, 36.93, 36.69, 34.45, 30.52, 29.06, 27.01, 24.91, 22.55, 16.56, 14.68, 11.99. HR-MS (ESI): Calcd for C_36_H_51_N_5_O_5_(M + H^+^): 634.3890; Found: 634.3891.

#### 4.2.17. 22-(2-(4-(4-(4-Methoxyphenyl)-piperazin-1-yl)-methyl)-1H-1,2,3-triazol-1-yl)-22-deoxy pleuromutilin (61)

Compound **61** was prepared from compound **7** and 1-(4-methoxyphenyl)-4-(prop-2-yn-1-yl)piperazine (**41)** in the same procedure as described for compound **48**. White powder; yield: 68%; ^1^H NMR (600 MHz, DMSO-d_6_) *δ* 7.01**–**6.84 (m, 2H), 6.83**–**6.78 (m, 1H), 6.16**–**6.07 (m, 1H), 5.57 (d, *J* = 8.6 Hz, 1H), 5.38**–**5.24 (m, 1H), 5.15**–**5.01 (m, 2H), 4.60**–**4.53 (m, 1H), 3.79**–**3.70 (m, 1H), 3.67 (s, 3H), 3.41 (t, *J* = 6.2 Hz, 1H), 3.34 (s, 8H), 3.01 (s, 2H), 2.59 (s, 2H), 2.39 (s, 1H), 2.17 (dd, *J* = 11.4, 18.7 Hz, 1H), 2.04 (ddt, *J* = 8.4, 14.6, 28.0 Hz, 2H), 1.68**–**1.56 (m, 2H), 1.46 (td, *J* = 4.1, 8.0 Hz, 1H), 1.41**–**1.30 (m, 2H), 1.30**–**1.21 (m, 3H), 1.18 (d, *J* = 8.5 Hz, 3H), 1.06 (d, *J* = 3.1 Hz, 3H), 0.99 (td, *J* = 4.5, 14.2 Hz, 1H), 0.81 (d, *J* = 6.9 Hz, 3H), 0.60 (dd, *J* = 7.0, 17.4 Hz, 3H). ^13 ^C NMR (151 MHz, DMSO) *δ* 217.48, 165.98, 162.82, 153.38, 141.21, 126.13, 125.81, 117.83, 115.87, 115.25, 115.08, 114.73, 73.00, 70.96, 57.60, 55.85, 55.67, 51.73, 45.40, 44.62, 43.77, 41.97, 41.95, 40.59, 36.93, 36.70, 34.45, 30.52, 29.06, 27.01, 24.91, 16.56, 14.69, 14.64, 11.98. HR-MS (ESI): Calcd for C_36_H_51_N_5_O_5_(M + H^+^): 634.3890; Found: 634.3963.

#### 4.2.18. 22-(2-(4-(4-(2-Nitrophenyl)-piperazin-1-yl)-methyl)-1H-1,2,3-triazol-1-yl)-22-deoxy pleuromutilin (62)

Compound **62** was prepared from compound **7** and 1-(2-nitrophenyl)-4-(prop-2-yn-1-yl)piperazine (**42)** in the same procedure as described for compound **48**. Yellow powder; yield: 59%; ^1^H NMR (600 MHz, DMSO-d_6_) *δ* 8.04 (s, 1H), 7.78 (dd, *J* = 8.1, 1.6 Hz, 1H), 7.57 (ddd, *J* = 8.6, 7.3, 1.6 Hz, 1H), 7.31 (dd, *J* = 8.3, 1.2 Hz, 1H), 7.12 (ddd, *J* = 8.2, 7.3, 1.1 Hz, 1H), 6.17**–**6.08 (m, 1H), 5.76 (s, 1H), 5.57 (d, *J* = 8.4 Hz, 1H), 5.39**–**5.25 (m, 2H), 5.14**–**5.01 (m, 2H), 4.55 (d, *J* = 6.1 Hz, 1H), 3.41 (t, *J* = 6.1 Hz, 1H), 3.33 (s, 5H), 3.01 (s, 4H), 2.50 (q, *J* = 1.9 Hz, 3H), 2.39 (d, *J* = 2.5 Hz, 1H), 2.22**–**2.12 (m, 1H), 2.11**–**1.98 (m, 3H), 1.69**–**1.58 (m, 2H), 1.51**–**1.43 (m, *J* = 5.9, 4.5 Hz, 1H), 1.39**–**1.31 (m, 2H), 1.19 (s, 3H), 1.06 (s, 3H), 0.81 (d, *J* = 7.0 Hz, 3H), 0.62 (d, *J* = 7.2 Hz, 3H). ^13 ^C NMR (151 MHz, DMSO) *δ* 217.45, 165.96, 145.67, 143.57, 141.17, 134.16, 125.76, 122.46, 121.96, 115.87, 72.97, 70.93, 57.66, 57.56, 55.38, 51.71, 45.43, 45.38, 44.60, 43.70, 41.96, 40.55, 39.65, 36.92, 36.74, 36.68, 34.43, 30.51, 29.06, 27.00, 24.91, 16.56, 14.95, 14.66, 12.00. HR-MS (ESI): Calcd for C_35_H_48_N_6_O_6_(M + H^+^): 649.3635; Found: 649.3704.

#### 4.2.19. 22-(2-(4-(4-(3-Nitrophenyl)-piperazin-1-yl)-methyl)-1H-1,2,3-triazol-1-yl)-22-deoxy pleuromutilin (63)

Compound **63** was prepared from compound **7** and 1-(3-nitrophenyl)-4-(prop-2-yn-1-yl)piperazine (**43)** in the same procedure as described for compound **48**. Yellow powder; yield: 42%; ^1^H NMR (600 MHz, DMSO-d_6_) *δ* 8.13 (s, 1H), 7.63 (s, 1H), 7.57 (dd, *J* = 1.9, 7.9 Hz, 1H), 7.46 (t, *J* = 8.1 Hz, 1H), 7.39 (dd, *J* = 2.3, 8.3 Hz, 1H), 6.17**–**6.08 (m, 1H), 5.57 (d, *J* = 8.4 Hz, 1H), 5.43**–**5.26 (m, 2H), 5.14**–**5.03 (m, 2H), 4.57 (d, *J* = 6.0 Hz, 1H), 3.42 (dt, *J* = 6.1, 22.1 Hz, 1H), 3.29 (s, 4H), 2.83 (s, 2H), 2.38 (s, 1H), 2.21**–**2.13 (m, 1H), 2.12**–**1.97 (m, 3H), 1.68**–**1.56 (m, 2H), 1.46 (ddt, *J* = 5.1, 10.3, 14.0 Hz, 1H), 1.42**–**1.28 (m, 3H), 1.26 (ddd, *J* = 3.1, 8.1, 16.4 Hz, 2H), 1.20 (s, 3H), 1.07 (d, *J* = 5.3 Hz, 3H), 0.99 (td, *J* = 4.3, 14.0 Hz, 1H), 0.81 (d, *J* = 6.9 Hz, 3H), 0.62 (d, *J* = 7.2 Hz, 3H). ^13 ^C NMR (151 MHz, DMSO) *δ* 217.43, 165.90, 152.33, 149.32, 141.21, 130.57, 121.76, 115.88, 113.12, 108.78, 73.02, 70.94, 57.68, 57.61, 55.37, 50.84, 45.40, 44.61, 43.77, 41.97, 40.61, 36.93, 36.69, 34.45, 30.58, 30.52, 29.06, 27.01, 24.96, 24.91, 16.55, 16.45, 14.96, 14.69, 11.98. HR-MS (ESI): Calcd for C_35_H_48_N_6_O_6_(M + H^+^): 649.3635; Found: 649.3604.

#### 4.2.20. 22-(2-(4-(4-(4-Nitrophenyl)-piperazin-1-yl)-methyl)-1H-1,2,3-triazol-1-yl)-22-deoxy pleuromutilin (64)

Compound **64** was prepared from compound **7** and 1-(4-nitrophenyl)-4-(prop-2-yn-1-yl)piperazine (**44)** in the same procedure as described for compound **48**. Yellow powder; yield: 62%; ^1^H NMR (600 MHz, DMSO-d_6_) *δ* 8.12**–**7.89 (m, 1H), 7.14**–**6.83 (m, 1H), 6.14 (ddd, *J* = 11.2, 17.8, 25.4 Hz, 1H), 5.60 (dd, *J* = 8.3, 49.9 Hz, 1H), 5.37**–**5.22 (m, 1H), 5.14**–**5.03 (m, 2H), 4.58 (d, *J* = 6.1 Hz, 1H), 4.26**–**3.92 (m, 1H), 3.69 (s, 1H), 3.51**–**3.36 (m, 3H), 3.34 (s, 6H), 2.59 (d, *J* = 33.4 Hz, 2H), 2.46**–**2.29 (m, 1H), 2.24**–**1.97 (m, 4H), 1.71**–**1.55 (m, 2H), 1.55**–**1.40 (m, 1H), 1.37 (d, *J* = 9.5 Hz, 2H), 1.34**–**1.21 (m, 4H), 1.16 (s, 1H), 1.07 (d, *J* = 6.7 Hz, 3H), 1.05**–**0.96 (m, 1H), 0.82 (dd, *J* = 7.0, 17.6 Hz, 3H), 0.61 (dd, *J* = 7.1, 16.4 Hz, 3H). ^13 ^C NMR (151 MHz, DMSO) *δ* 167.75, 166.01, 155.13, 141.30, 141.19, 126.19, 115.89, 115.80, 113.03, 73.05, 73.01, 70.90, 70.68, 57.67, 57.60, 50.82, 45.43, 45.39, 44.63, 43.94, 43.74, 42.00, 40.57, 36.99, 36.75, 34.46, 30.58, 29.05, 27.03, 24.96, 16.54, 16.46, 14.95, 14.67, 12.03. HR-MS (ESI): Calcd for C_35_H_48_N_6_O_6_(M + H^+^): 649.3635; Found: 649.3713.

#### 4.2.21. 22-(2-(4-(4-(2-Hydroxyphenyl)-piperazin-1-yl)-methyl)-1H-1,2,3-triazol-1-yl)-22-deoxy pleuromutilin (65)

Compound **65** was prepared from compound **7** and 2-(4-(prop-2-yn-1-yl)piperazin-1-yl)phenol (**45)** in the same procedure as described for compound **48**. White powder; yield: 32%; ^1^H NMR (600 MHz, DMSO-d_6_) *δ* 8.90 (s, 1H), 8.00 (s, 1H), 6.87**–**6.79 (m, 2H), 6.77**–**6.69 (m, 2H), 6.13 (dd, *J* = 17.8, 11.2 Hz, 1H), 5.75 (s, 1H), 5.57 (d, *J* = 8.4 Hz, 1H), 5.37**–**5.25 (m, 2H), 5.15**–**5.03 (m, 2H), 4.54 (d, *J* = 6.1 Hz, 1H), 3.66**–**3.62 (m, 2H), 3.41 (t, *J* = 6.1 Hz, 1H), 3.32 (s, 3H), 2.94 (s, 3H), 2.50 (q, *J* = 1.9 Hz, 3H), 2.40 (d, *J* = 2.6 Hz, 1H), 2.23**–**2.12 (m, 1H), 2.09**–**1.98 (m, 3H), 1.69**–**1.56 (m, 2H), 1.51**–**1.44 (m, 1H), 1.38**–**1.31 (m, 2H), 1.30**–**1.25 (m, 1H), 1.19 (s, 3H), 1.07 (s, 3H), 0.81 (d, *J* = 7.0 Hz, 3H), 0.62 (d, *J* = 7.1 Hz, 3H). ^13 ^C NMR (151 MHz, DMSO) *δ* 217.51, 166.02, 150.52, 147.71, 144.10, 141.19, 140.26, 125.66, 123.20, 119.81, 118.98, 115.97, 115.87, 72.96, 70.92, 57.56, 55.38, 53.10, 51.59, 50.43, 45.39, 44.60, 43.72, 41.96, 40.53, 36.92, 36.69, 34.44, 30.52, 29.06, 27.01, 24.91, 16.58, 14.67, 12.01. HR-MS (ESI): Calcd for C_35_H_49_N_5_O_5_(M + H^+^): 620.3733; Found: 620.3784.

#### 4.2.22. 22-(2-(4-(4-(3-Hydroxyphenyl)-piperazin-1-yl)-methyl)-1H-1,2,3-triazol -1-yl)-22-deoxy pleuromutilin (66)

Compound **66** was prepared from compound **7** and 3-(4-(prop-2-yn-1-yl)piperazin-1-yl)phenol (**46)** in the same procedure as described for compound **48**. White powder; yield: 33%; ^1^H NMR (600 MHz, Chloroform-*d*) *δ* 7.71 (s, 1H), 7.11 (t, *J* = 8.1 Hz, 1H), 6.51**–**6.47 (m, 1H), 6.45**–**6.39 (m, 2H), 6.37**–**6.32 (m, 1H), 5.83 (d, *J* = 8.6 Hz, 1H), 5.38 − 5.30 (m, 2H), 5.23 (dd, *J* = 1.4, 17.4 Hz, 1H), 5.15**–**5.03 (m, 2H), 3.86 (s, 2H), 3.36 (dd, *J* = 6.5, 10.5 Hz, 1H), 3.23 (s, 4H), 2.75 (s, 4H), 2.31**–**2.19 (m, 3H), 2.09 (s, 1H), 1.78 (dd, *J* = 3.2, 14.5 Hz, 1H), 1.70**–**1.61 (m, 2H), 1.57**–**1.38 (m, 4H), 1.35 (s, 3H), 1.33**–**1.24 (m, 2H), 1.19 (s, 3H), 1.14 (td, *J* = 4.4, 14.1 Hz, 1H), 0.89 (d, *J* = 7.0 Hz, 3H), 0.72 (d, *J* = 7.1 Hz, 3H). ^13 ^C NMR (151 MHz, CDCl_3_) *δ* 216.82, 164.94, 160.87, 156.73, 138.56, 130.01, 117.62, 108.58, 103.40, 94.02, 77.21, 77.11, 76.99, 76.78, 74.53, 71.01, 58.02, 52.53, 51.63, 48.63, 45.42, 44.75, 44.00, 41.86, 36.58, 36.02, 34.41, 30.88, 30.34, 26.77, 26.36, 24.80, 16.80, 14.74, 11.44. HR-MS (ESI): Calcd for C_35_H_49_N_5_O_5_(M + H^+^): 620.3733; Found: 620.3662.

#### 4.2.23. 22-(2-(4-(4-(4-Hydroxyphenyl)-piperazin-1-yl)-methyl)-1H-1,2,3-triazol-1-yl)-22-deoxy pleuromutilin (67)

Compound **67** was prepared from compound **7** and 4-(4-(prop-2-yn-1-yl)piperazin-1-yl)phenol (**47)** in the same procedure as described for compound **48**. White powder; yield: 39%; ^1^H NMR (600 MHz, Chloroform-*d*) *δ* 7.74 (s, 1H), 6.86**–**6.81 (m, 2H), 6.80**–**6.75 (m, 2H), 6.42 (dd, *J* = 11.0, 17.4 Hz, 1H), 5.83 (d, *J* = 8.6 Hz, 1H), 5.38**–**5.30 (m, 2H), 5.23 (dd, *J* = 1.5, 17.4 Hz, 1H), 5.15**–**5.02 (m, 2H), 3.86 (s, 2H), 3.36 (dd, *J* = 6.5, 10.6 Hz, 1H), 3.13 (s, 4H), 2.78 (s, 4H), 2.34**–**2.17 (m, 3H), 2.09 (s, 1H), 1.78 (dd, *J* = 3.1, 14.5 Hz, 1H), 1.71**–**1.63 (m, 1H), 1.57**–**1.47 (m, 2H), 1.47**–**1.37 (m, 2H), 1.36**–**1.26 (m, 6H), 1.19 (s, 3H), 1.14 (td, *J* = 4.5, 14.2 Hz, 1H), 0.89 (d, *J* = 7.0 Hz, 3H), 0.73 (d, *J* = 7.1 Hz, 3H). ^13 ^C NMR (151 MHz, CDCl_3_) *δ* 216.51, 193.51, 164.99, 139.57, 138.56, 118.65, 118.64, 117.62, 115.91, 87.31, 77.20, 76.99, 76.78, 74.54, 70.98, 57.98, 55.58, 52.86, 51.59, 50.27, 45.40, 44.74, 44.00, 41.85, 36.54, 36.04, 34.37, 32.90, 30.33, 26.78, 26.37, 24.80, 16.78, 14.64, 11.44. HR-MS (ESI): Calcd for C_35_H_49_N_5_O_5_(M + H^+^): 620.3733; Found: 620.3702.

#### 4.2.24. General synthesis method of secondary amine derivatives containing propynyl group (74–79)

A solution of secondary amine derivatives (13.67 mmol) in ethyl acetate (30 ml) was stirred at room temperature in a three-necked round bottom flask, and DIEPA (2.12 g, 16.40 mmol) was added. Then the reaction was cooled in an ice bath and 3-bromopropyne (1.63 g, 13.67 mmol) was added dropwise. The solution was stirred at 78 °C for 6 h. After adding CHCl_3_ (20 ml) and ice-cold water (20 ml), the reaction solution was continuously stirred for 0.5 h and poured into a separating funnel. The organic phase was washed with a saturated aqueous solution of NaCl and water, respectively. Then the organic phase was dried with anhydrous Na_2_SO_4_ and evaporated in vacuum. The crude product was purified by column chromatography (methanol: DCM = 1:20) using silica gel to afford the compounds **74–79**, respectively.

#### 4.2.25. 22-(2-(4-(Diethylamino)methyl)-1H-1,2,3-triazol-1-yl)-22-deoxy pleuromutilin (80)

Compound **80** was prepared from compound **7** and *N,N*-diethylprop-2-yn-1-amine (**74)** in the same procedure as described for compound **48**. White powder; yield: 51%; ^1^H NMR (600 MHz, DMSO-d_6_) *δ* 7.91 (s, 1H), 6.12 (dd, *J* = 17.8, 11.2 Hz, 1H), 5.56 (d, *J* = 8.4 Hz, 1H), 5.33**–**5.20 (m, 2H), 5.13**–**5.01 (m, 2H), 4.54 (d, *J* = 6.1 Hz, 1H), 3.69 (s, 2H), 3.41 (t, *J* = 6.1 Hz, 1H), 3.32 (s, 1H), 2.50 (p, *J* = 1.9 Hz, 2H), 2.44**–**2.41 (m, 3H), 2.21**–**2.13 (m, 1H), 2.10**–**1.98 (m, 3H), 1.68**–**1.58 (m, 2H), 1.46 (dtd, *J* = 14.2, 7.1, 3.4 Hz, 1H), 1.40**–**1.33 (m, 1H), 1.30**–**1.27 (m, 1H), 1.18 (s, 3H), 1.06 (s, 3H), 1.00 (t, *J* = 7.0 Hz, 7H), 0.81 (d, *J* = 7.0 Hz, 3H), 0.61 (d, *J* = 7.1 Hz, 3H). ^13 ^C NMR (151 MHz, DMSO) *δ* 217.50, 166.04, 141.19, 125.38, 115.84, 72.96, 70.84, 57.57, 51.49, 47.02, 46.48, 45.39, 44.59, 43.74, 41.94, 36.93, 36.70, 34.44, 31.43, 30.53, 29.06, 27.01, 24.91, 22.54, 16.54, 14.71, 14.44, 12.45, 12.01. HR-MS (ESI): Calcd for C_29_H_46_N_4_O_4_(M + H^+^): 515.3519; Found: 515.3613.

#### 4.2.26. 22-(2-(4-(Dicyclohexylamino)methyl)-1H-1,2,3-triazol-1-yl)-22-deoxy pleuromutilin (81)

Compound **81** was prepared from compound **7** and *N*-cyclohexyl-*N*-(prop-2-yn-1-yl)cyclohexanamine (**75)** in the same procedure as described for compound **48**. White powder; yield: 55%; ^1^H NMR (600 MHz, DMSO-d_6_) *δ* 7.77 (s, 1H), 6.13 (dd, *J* = 17.8, 11.2 Hz, 1H), 5.56 (d, *J* = 8.4 Hz, 1H), 5.30**–**5.19 (m, 2H), 5.13**–**5.00 (m, 2H), 4.54 (d, *J* = 6.1 Hz, 1H), 3.76 (s, 2H), 3.41 (t, *J* = 6.2 Hz, 1H), 3.32 (s, 1H), 2.50 (p, *J* = 1.8 Hz, 4H), 2.39**–**2.36 (m, 1H), 2.24**–**2.12 (m, 1H), 2.09**–**1.98 (m, 3H), 1.72**–**1.43 (m, 14H), 1.40**–**1.20 (m, 9H), 1.16 (s, 4H), 1.06 (s, 3H), 0.88**–**0.84 (m, 1H), 0.81 (d, *J* = 7.0 Hz, 3H), 0.59 (d, *J* = 7.0 Hz, 3H). ^13 ^C NMR (151 MHz, DMSO) *δ* 217.50, 166.05, 141.19, 124.62, 115.79, 72.96, 70.73, 57.86, 57.60, 51.52, 45.40, 44.59, 43.74, 41.94, 40.54, 39.78, 36.93, 36.68, 34.44, 31.88, 31.76, 31.71, 31.43, 30.52, 29.06, 27.02, 26.29, 26.27, 26.17, 24.92, 22.54, 16.54, 14.66, 14.44, 14.44, 12.02, 0.58. HR-MS (ESI): Calcd for C_37_H_58_N_4_O_4_(M + H^+^): 623.4458; Found: 623.4382.

#### 4.2.27. 22-(2-(4-(Morpholinomethyl)methyl)-1H-1,2,3-triazol-1-yl)-22-deoxy pleuromutilin (82)

Compound **82** was prepared from compound **7** and 4-(prop-2-yn-1-yl)morpholine (**76)** in the same procedure as described for compound **48**. White powder; yield: 41%; ^1^H NMR (600 MHz, DMSO-d_6_) *δ* 7.97 (s, 1H), 6.12 (dd, *J* = 11.2, 17.8 Hz, 1H), 5.56 (d, *J* = 8.4 Hz, 1H), 5.32 (d, *J* = 17.5 Hz, 1H), 5.14**–**5.02 (m, 2H), 4.57 (d, *J* = 6.1 Hz, 1H), 3.55 (s, 6H), 3.41 (t, *J* = 6.1 Hz, 1H), 2.45**–**2.36 (m, 5H), 2.11**–**1.95 (m, 3H), 1.68**–**1.56 (m, 2H), 1.46 (ddt, *J* = 5.1, 10.1, 13.7 Hz, 1H), 1.41**–**1.31 (m, 2H), 1.31**–**1.21 (m, 4H), 1.18 (s, 3H), 1.06 (s, 3H), 0.99 (td, *J* = 4.4, 14.1 Hz, 1H), 0.81 (d, *J* = 7.0 Hz, 3H), 0.61 (d, *J* = 7.1 Hz, 3H). ^13 ^C NMR (151 MHz, DMSO) *δ* 217.48, 166.04, 141.22, 125.73, 115.86, 72.99, 71.33, 70.93, 66.62, 66.60, 57.60, 53.15, 51.55, 45.40, 44.61, 43.77, 41.96, 40.57, 36.93, 36.70, 35.80, 34.45, 30.53, 29.05, 27.02, 24.91, 16.55, 14.69, 11.99. HR-MS (ESI): Calcd for C_29_H_44_N_4_O_5_(M + H^+^): 529.3312; Found: 529.3427.

#### 4.2.28. 22-(2-(4-(4-Hydroxypiperidin-1-yl)methyl)-1H-1,2,3-triazol-1-yl)-22-deoxy pleuromutilin (83)

Compound **83** was prepared from compound **7** and 1-(prop-2-yn-1-yl)piperidin-4-ol (**77)** in the same procedure as described for compound **48**. White powder; yield: 70%; ^1^H NMR (600 MHz, DMSO-d_6_) *δ* 7.92 (s, 1H), 6.12 (dd, *J* = 11.2, 17.8 Hz, 1H), 5.56 (d, *J* = 8.4 Hz, 1H), 5.34**–**5.21 (m, 2H), 5.14**–**5.03 (m, 2H), 4.56 (dd, *J* = 5.0, 9.0 Hz, 2H), 3.52 (s, 2H), 3.41 (q, *J* = 8.9, 11.6 Hz, 2H), 2.70**–**2.65 (m, 2H), 2.39 (d, *J* = 2.5 Hz, 1H), 2.22**–**2.14 (m, 1H), 2.11**–**1.98 (m, 5H), 1.71**–**1.56 (m, 4H), 1.46 (ddt, *J* = 5.3, 10.4, 14.1 Hz, 1H), 1.39**–**1.20 (m, 7H), 1.17 (s, 3H), 1.06 (s, 3H), 1.05**–**0.97 (m, 1H), 0.81 (d, *J* = 7.0 Hz, 3H), 0.60 (d, *J* = 7.1 Hz, 3H). ^13 ^C NMR (151 MHz, DMSO) *δ* 217.48, 166.04, 141.22, 125.49, 115.86, 73.00, 70.90, 66.76, 57.60, 56.50, 52.97, 51.53, 51.00, 45.40, 44.60, 43.79, 41.95, 40.57, 36.93, 36.70, 34.88, 34.45, 30.53, 29.06, 27.02, 24.92, 19.03, 16.55, 14.66, 11.99. HR-MS (ESI): Calcd for C_30_H_46_N_4_O_5_(M + H^+^): 543.3468; Found: 543.3522.

#### 4.2.29. 22-(2-(4-(3-(Hydroxymethyl)piperidin-1-yl)methyl)-1H-1,2,3-triazol-1-yl)-22-deoxy pleuromutilin (84)

Compound **84** was prepared from compound **7** and (1-(prop-2-yn-1-yl)piperidin-3-yl)methanol (**78)** in the same procedure as described for compound **48**. White powder; yield: 68%; ^1^H NMR (600 MHz, Chloroform-*d*) *δ* 7.79 (s, 1H), 6.42 (ddd, *J* = 1.9, 11.0, 17.4 Hz, 1H), 5.81 (dd, *J* = 2.5, 8.6 Hz, 1H), 5.38**–**5.30 (m, 1H), 5.26**–**5.20 (m, 1H), 5.15**–**4.99 (m, 2H), 3.85 (s, 2H), 3.62 (dd, *J* = 5.2, 10.8 Hz, 1H), 3.56 (dd, *J* = 6.4, 10.8 Hz, 1H), 3.36 (s, 1H), 3.00 (d, *J* = 11.1 Hz, 1H), 2.87 (s, 1H), 2.37 (s, 1H), 2.33**–**2.15 (m, 4H), 2.09 (s, 2H), 1.95 (s, 1H), 1.79 (ddd, *J* = 10.2, 16.3, 31.3 Hz, 3H), 1.74**–**1.59 (m, 3H), 1.56**–**1.37 (m, 3H), 1.33 (d, *J* = 1.1 Hz, 4H), 1.32**–**1.24 (m, 2H), 1.20**–**1.18 (m, 3H), 1.17**–**1.10 (m, 1H), 0.89 (d, *J* = 7.0 Hz, 3H), 0.71 (t, *J* = 7.1 Hz, 3H). ^13 ^C NMR (151 MHz, CDCl_3_) *δ* 216.57, 164.93, 138.55, 132.10, 117.61, 105.90, 100.00, 77.21, 77.00, 76.78, 74.53, 71.01, 64.87, 57.99, 53.72, 53.34, 51.61, 45.40, 44.72, 44.01, 41.84, 36.55, 36.01, 34.38, 30.33, 26.78, 26.35, 24.79, 16.81, 14.66, 11.43. HR-MS (ESI): Calcd for C_31_H_48_N_4_O_5_(M + H^+^): 557.3625; Found: 557.3604.

#### 4.2.30. 22-(2-(4-(4-(2-Hydroxyethyl)piperidin-1-yl)methyl)-1H-1,2,3-triazol-1-yl)-22-deoxy pleuromutilin (85)

Compound **85** was prepared from compound **7** and 2-(1-(prop-2-yn-1-yl)piperidin-4-yl)ethan-1-ol (**79)** in the same procedure as described for compound **48**. White powder; yield: 75%; ^1^H NMR (600 MHz, Chloroform-*d*) *δ* 6.42 (dd, *J* = 11.0, 17.4 Hz, 1H), 5.82 (d, *J* = 8.5 Hz, 1H), 5.38**–**5.30 (m, 2H), 5.23 (dd, *J* = 1.5, 17.4 Hz, 1H), 5.14**–**4.99 (m, 2H), 3.82 (s, 2H), 3.71 (t, *J* = 6.3 Hz, 2H), 3.36 (dd, *J* = 6.5, 10.4 Hz, 1H), 2.34**–**2.15 (m, 3H), 2.13**–**2.06 (m, 2H), 1.81**–**1.73 (m, 4H), 1.72**–**1.61 (m, 2H), 1.57**–**1.50 (m, 4H), 1.50**–**1.38 (m, 3H), 1.34 (s, 5H), 1.31**–**1.22 (m, 2H), 1.19 (s, 4H), 1.14 (td, *J* = 4.5, 14.2 Hz, 1H), 0.89 (d, *J* = 7.1 Hz, 4H), 0.72 (d, *J* = 7.1 Hz, 4H). ^13 ^C NMR (151 MHz, CDCl_3_) *δ* 216.55, 199.84, 138.55, 125.15, 117.61, 94.50, 77.20, 76.99, 76.78, 74.54, 70.94, 63.20, 60.37, 58.00, 53.37, 51.59, 45.40, 44.74, 44.00, 41.84, 36.56, 36.03, 34.38, 31.80, 30.34, 26.78, 26.35, 24.80, 18.15, 16.79, 14.67, 11.44. HR-MS (ESI): Calcd for C_32_H_50_N_4_O_5_(M + H^+^): 571.3781; Found: 571.3830.

### 4.3. *In vitro* efficacy of pleuromutilin derivatives

#### 4.3.1. Minimal inhibitory concentration (MIC) and minimum bactericidal concentration (MBC) testing

The MIC of these novel pleuromutilin derivatives against methicillin-resistant *S. aureus* (ATCC 43300), *S. aureus* (ATCC 29213), *S. aureus* (AD3) and *S. aureus* (144) were determined by using pleuromutilin and tiamulin as positive control. MIC values were determined by the broth micro dilution methods in accordance with the Clinical and Laboratory Standards Institute (2012). Stock solutions of these compounds were dissolved in methanol to make a stock solution with a concentration of 1280 µg/mL. The working solutions (640 µg/mL) were obtained by diluting stock solutions in sterile Mueller-Hinton (MH) broth. The bacterial solution and solutions of these compounds with a final concentration of 0.016**–**32 µg/mL were distributed into 96-well plates, with a total volume of 200 µL. Three parallel experiments for each compound concentration. The plates were incubated at 37 °C for 24 h. The MIC value was recorded as the minimum drug concentration that completely inhibits the visible growth of test bacteria.

MBC values were determined according to CLSI (2003) and previously report with slightly modification^18^^,^^28^^,^^31^. After obtaining the MIC results, the 96-well plates were incubated at 37 °C for 24 h. 100 µL of the bacterial solution from the wells with no visible growth was inoculated on MH agar plates. The MH agar plates were incubated at 37 °C for colony count. The MBC was determined as the lowest concentration of compound, which reduces the viable counts for 99.9%.

#### 4.3.2. Constant concentration time-kill curves

The time-kill curve determination is an *in vitro* bactericidal kinetics model, which is used to study the sustained killing effect of bacteria within 24 h at a certain drug concentration^22^. The bactericidal activity of compounds **50**, **62** and **64** was determined by the time-kill curve as reported in our previous work^18^^,^^22^. MRSA grown in MH broth were diluted to approximately 1 × 10^6^ CFU/mL. Compounds **50**, **62** and **64** were prepared into working solutions with concentrations of 1 × MIC, 2 × MIC, 4 × MIC, 8 × MIC, 16 × MIC respectively, and then added to the above bacterial solutions. 100 µL of samples were collected from the subculture inoculums before culture (0 h) and 3 h, 6 h, 9 h and 24 h after culture, and serially diluted 10-flod with sterile saline. The dilution of each gradient (25 µL) was uniformly suspended on the MH agar plates. The total bacterial CFU/mL on the plates were counted to calculate the bacterial colonies after up to 20 h of incubation at 37 °C. The results are represented by Mean ± SD, with the log_10_ CFU/mL of bacteria counts as the ordinate and time as the abscissa, the time-kill curves of different concentrations of compounds against MRSA were established respectively.

### 4.4. SPR binding studies

SPR biosensing experiments were performed at 4 °C using the bScreen LB 991 Label-free Microarray System (Berthold Technologies, Germany) and the photo-cross-linker sensor chip. The BIODOT™ AD-1520 Array Printer (BIODOT Inc., USA) was used for printing samples and control on the chip surface. A UV Spectroirradiator 1020 (Amersham Life Science, USA) was used for the photo-cross-linking reaction quickly after the array print.

#### 4.4.1. Preparation of *50S* ribosome

As described in the reference^32–34^ with minor modifications, the ribosome of *S. aureus* were extracted and purified to obtain *50S* ribosome. MRSA (ATCC 43300) was incubated overnight at 37 °C. The bacteria solution was inoculated into a new culture solution, and cells were collected at an OD_600_ = 1.5. The bacterial culture was centrifuged 3 times at 3,500 rpm using a TX-400 rotor at 4 °C for 30 min, and then the supernatant was discarded. Cells were washed twice with 10 mM Tris-HCl (pH = 7.5), and suspend the precipitate with buffer A (20 mM HEPES-HCl pH = 7.5, 100 mM NH_4_Cl, 21 mM Mg(OAc)_2_, 1 mM EDTA, 1 mM DTT). Cells were lysed by repeated (>3×) snap-freezing and thawing, then ultrasonic disintegration was performed. To remove cell debris, the crushed product was centrifuged at 20,000 rpm for 90 min at 4 °C.The lysate layered on 15 ml of a sucrose cushion (10 mM HEPES-HCl pH = 7.5, 500 mM KCl, 25 mM Mg(OAc)_2_, 1.1 M sucrose, 0.5 mM EDTA, 1 mM DTT) and centrifuged in the ultracentrifuge(Optima^TM^ TL 100) at 45,000 rpm using a type70Ti rotor at 4 °C for 15 h to obtain ribosomal particles. Pellet was resuspended in 3 ml of buffer E (10 mM HEPES-HCl pH = 7.5, 100 mM KCl, 1 mM Mg(OAc)_2_, 0.5 mM EDTA, 1 mM DTT), then layered on 9 ml of 7–30% sucrose gradient and ultracentrifuged at 17,100 rpm using a typeSW40Ti rotor at 4 °C for 15 h. The gradient was analysed on the AKTA explorer system. The fractions corresponding to *50S* ribosome were collected and PEG 20000 was added to a final concentration of 4.5% w/v. Ribosomes were pelleted by centrifugation at 20,000 rpm for 12 min, the pellet was gently dissolved in 200 µL of storage buffer (10 mM HEPES-HCl pH = 7.5, 15 mM KCl, 60 mM NH_4_Cl, 10 mM Mg(OAc)_2_, 1 mM DTT) and then flash-frozen for storage at −80 °C.

#### 4.4.2. Binding studies

Dilute these compounds to a concentration of 10 mM with DMSO as the stationary phase printing working solution. After that, the printing working solution was printed on the sensor chip by the array printer, and each sample was printed four times repeatedly. Four groups of positive control dots (12 rapamycin dots, 3 in each corner) were printed on the four corners of the sensor chip respectively. The sample dots were dried in vacuum, and the sensor chip was quickly transferred to the UV spectroirradiator for a photo-cross-linking reaction. Subsequently, the sensor chip was washed with DMF, methanol, and water for 15 min in turn, followed by blowing dry under nitrogen, and then assembled with Flowcell Cover.

The binding studies were performed with PBST running buffer (pH = 7.4, 0.1% Tween 20) at a constant flow rate of 0.5 µL/s in instrument. *50S* Ribosome was diluted separately with PBST running buffer to 200 nM, 400 nM, 800 nM, 1600 nM and 3200 nM and injected consecutively for 600 s at associating stage, followed by running buffer for 360 s at each dissociating stage. Afterwards, the surface was regenerated to remove any remaining bound material with a pulse of 10 mM glycine-HCl (pH = 2.0) at 2 µL/s for 300 s.

To validate detection of the chemical compound-protein interactions, rapamycin was used as a system control, DMSO as a negative control and performed kinetic constant tests with FKBP12 immediately after the sample tests.

According to the real-time detection results of the SPR instrument, the kinetic curve of the interaction was fitted and the affinity parameters were output. The process and analysis of association rate constants (K_a_) and dissociation rate constants (K_d_) and the equilibrium dissociation constant (K_D_) were performed using the data analysis software of the microarray system. The kinetics of *50 s* Ribosome binding with matched these novel pleuromutilin derivatives could be analysed by 1: 1 Langmuir model with mass transfer limitations for binding kinetics determination.

### 4.5. Molecular docking

Molecular docking studies were performed by means of AutoDock Vina^35^. The conformation of tiamulin in the X-ray crystal structure of the *50S* ribosome (PDB ID code: 1XBP)^10^ was used to evaluate the accuracy of the docking performance. The receptor used for docking was extracted from the X-ray crystal structure of a *50S* ribosomal subunit (1XBP) in which tiamulin was removed. The downloaded receptor was protonated using the default parameters of H++ (http://biophysics.cs.vt.edu/). All compounds were prepared with Avogadro 1.1.1^36^, with a 5000 steps Steepest Descent as well as 1000 steps Conjugate Gradients geometry optimisation using MMFF94 force field. The centre (*x*, *y*, *z*) of the grid box was set to 17.0, 77.9, 2.6, and dimensions (Å) of the grid box were 15.8, 22.8, 16.0. For the selected compounds (**50** and **64)**, the highest ranked binding pose and the binding mode at the active site of *50S* ribosome were used for graphical representation in PyMOL1.8.0.4 (https://pymol.org/2/).

### 4.6. Cytotoxicity assay

Using the MTT assay, the cytotoxic potential of all synthesised compounds were evaluated on RAW 264.7 cells as described in the references^18^. The cells were seeded into 96-well plates at a density of 1.0 × 10^5^ cells per well and incubated at 37 °C for 24 h. These compounds were then diluted into a working solution with a concentration of 8 µg/mL to treat the cells and cultured for 16 h incubation at 37 °C. After that, the cells were incubated with 100 µL/well of MTT (0.5 mg/mL in PBS) for another 4 h under 5% CO_2_ 37 °C. After the medium was removed, 150 µL DMSO was added to each well to dissolve the cells. Absorbance at 490 nm was recorded using a microplate spectrophotometer after 30 min incubation (BIO-TEK Instrument Inc., USA).

## Supplementary Material

Supplemental Material
