# Supplementary material for: A click chemistry approach to pleuromutilin derivatives, evaluation of anti-MRSA activity and elucidation of binding mode by surface plasmon resonance and molecular docking
Source: J Enzyme Inhib Med Chem. 2021 Nov 25;36(1):2087–103. doi: 10.1080/14756366.2021.1977931 (PMC8635623; doi:10.1080/14756366.2021.1977931)
Supplement: Supplemental Material [file IENZ_A_1977931_SM7995.pdf]

Supplementary Materials

A click chemistry approach to pleuromutilin derivatives, evaluation of anti-MRSA activity and elucidation of binding mode by surface plasmon resonance and molecular docking

Zhe Zhang[a], Zhao-Sheng Zhang[a], Xiao Wang[a], Gao-Lei Xi[b], Zhen Jin[a,c] and You-Zhi Tang[a,c]

[a]*Guangdong Provincial Key Laboratory of Veterinary Pharmaceutics Development and Safety Evaluation, College of Veterinary Medicine, South China Agricultural University, Guangzhou, 510642, China*

[b]*Technology Center for China Tobacco Henan Industrial Limited Company, Zhengzhou, 450000, China*

[c]*Guangdong Laboratory for Lingnan Modern Agriculture, Guangzhou, 510642, China*

Correspondence should be addressed:

College of Veterinary Medicine, South China Agricultural University, No. 483 Wushan Road, Tianhe District, Guangzhou 510642, China

You-Zhi Tang,

Email: *youzhitang@scau. edu. cn*

The supplementary materials include:

^{1}H NMR, ^{13}C NMR and HR Mass (ESI) spectrum of the compounds (**Figure SI 1~ Figure SI 26**)

SPR binding signal curve (the concentration gradient curves) of each compound and controls to *50S* ribosome (**Figure SI 27~ Figure SI 34**)

Docking mode of tiamulin (green) to *50S* ribosome (1XBP). (**Figure SI 35**)

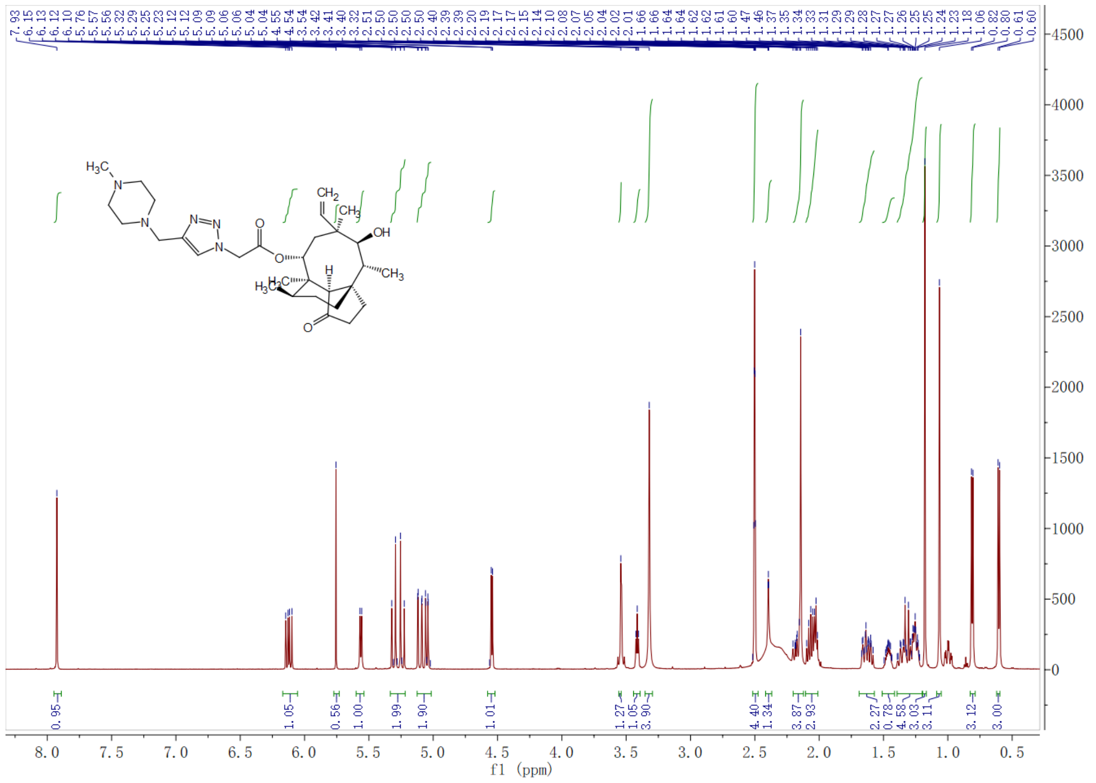

Figure SI 1-1. ^{1}H-NMR spectrum (DMSO-*d6*, 600 MHz) of compound **48**.

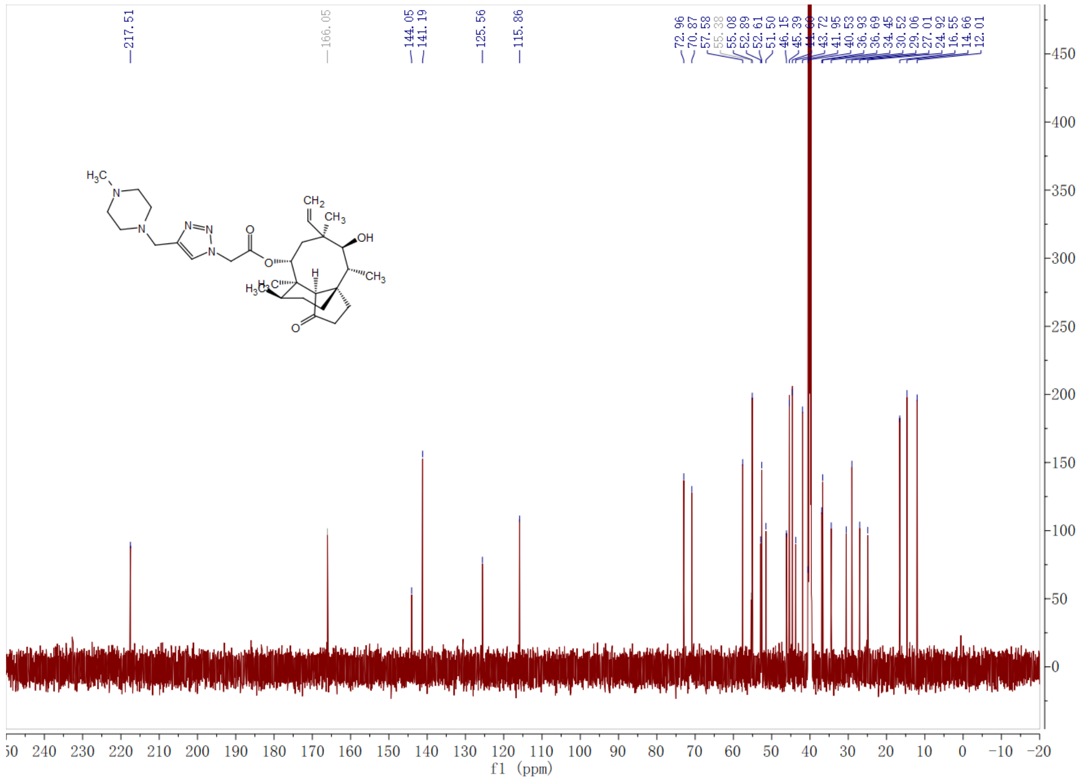

Figure SI 1-2. ^{13}C-NMR spectrum (DMSO-*d6*, 151 MHz) of compound **48.**

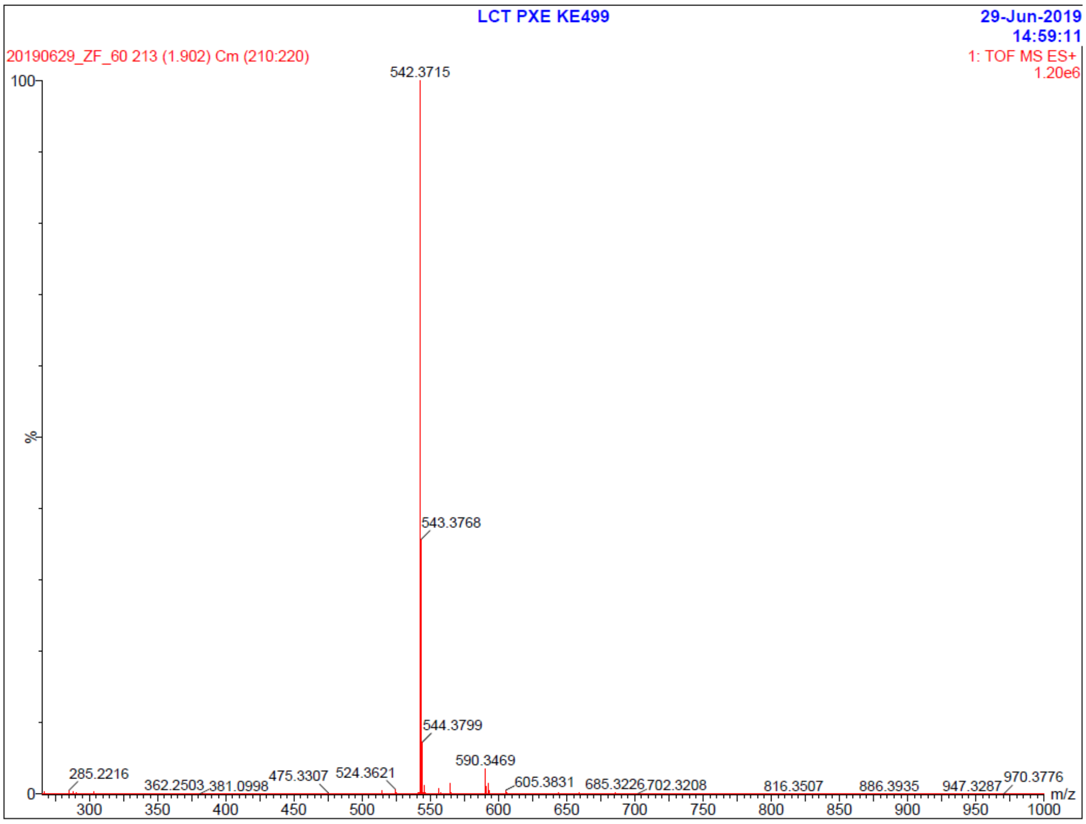

Figure SI 1-3. HR Mass spectrum (ESI) of compound **48**.

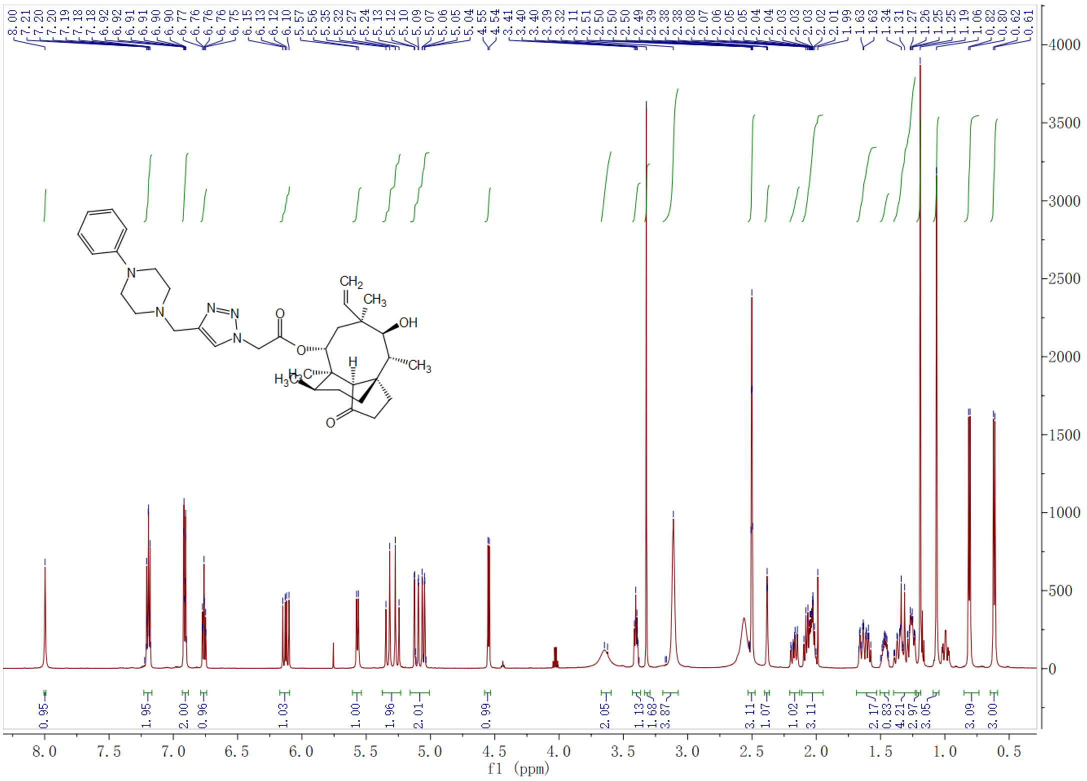

Figure SI 2-1. ^{1}H-NMR spectrum (DMSO-*d6*, 600 MHz) of compound **49.**

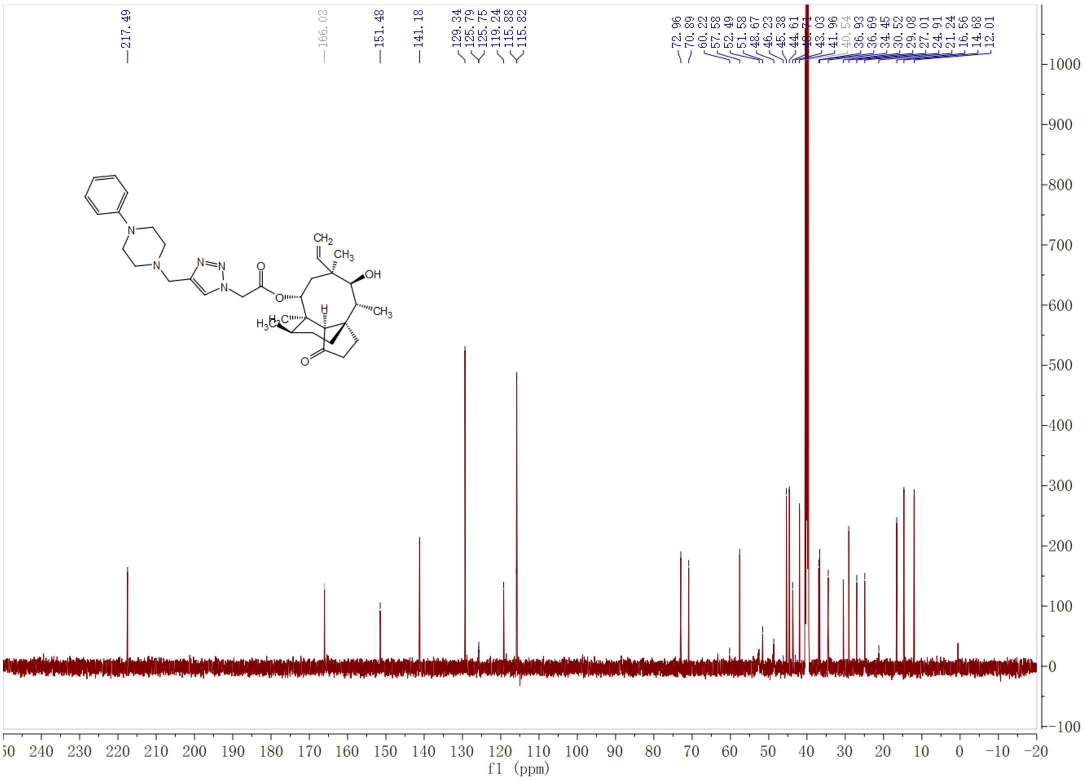

Figure SI 2-2. ^{13}C-NMR spectrum (DMSO-*d6*, 151 MHz) of compound **49.**

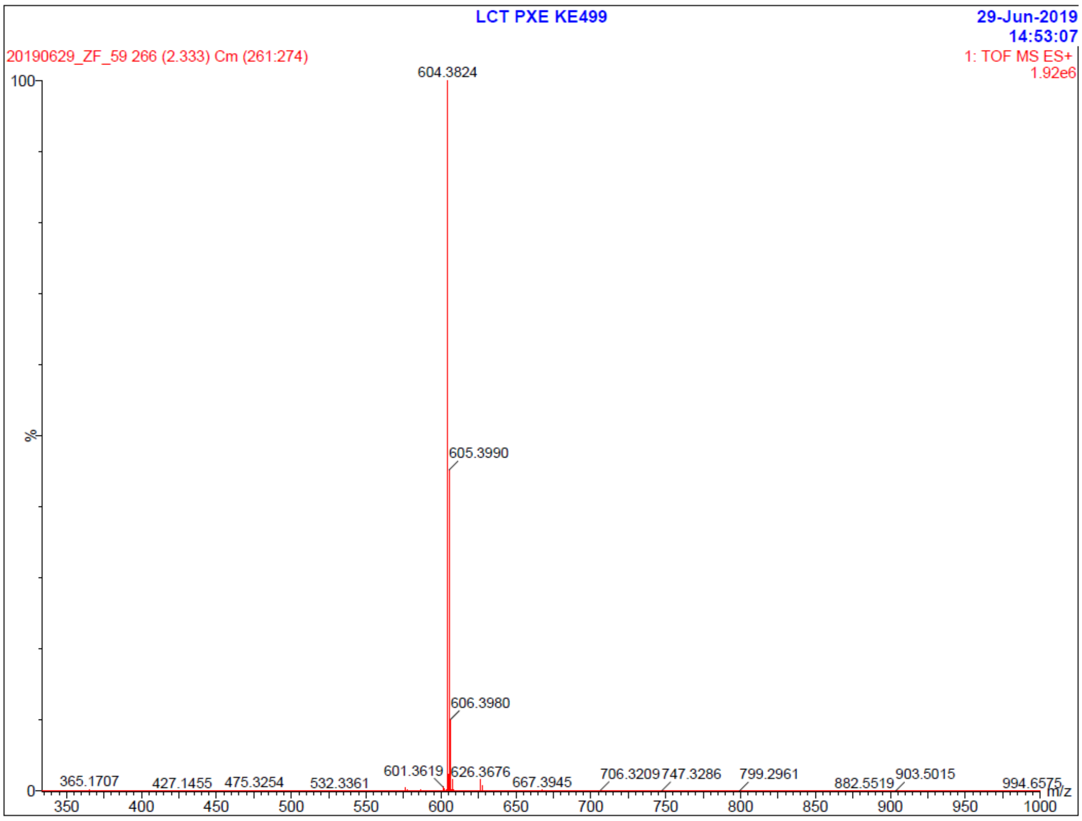

Figure SI 2-3. HR Mass spectrum (ESI) of compound **49.**

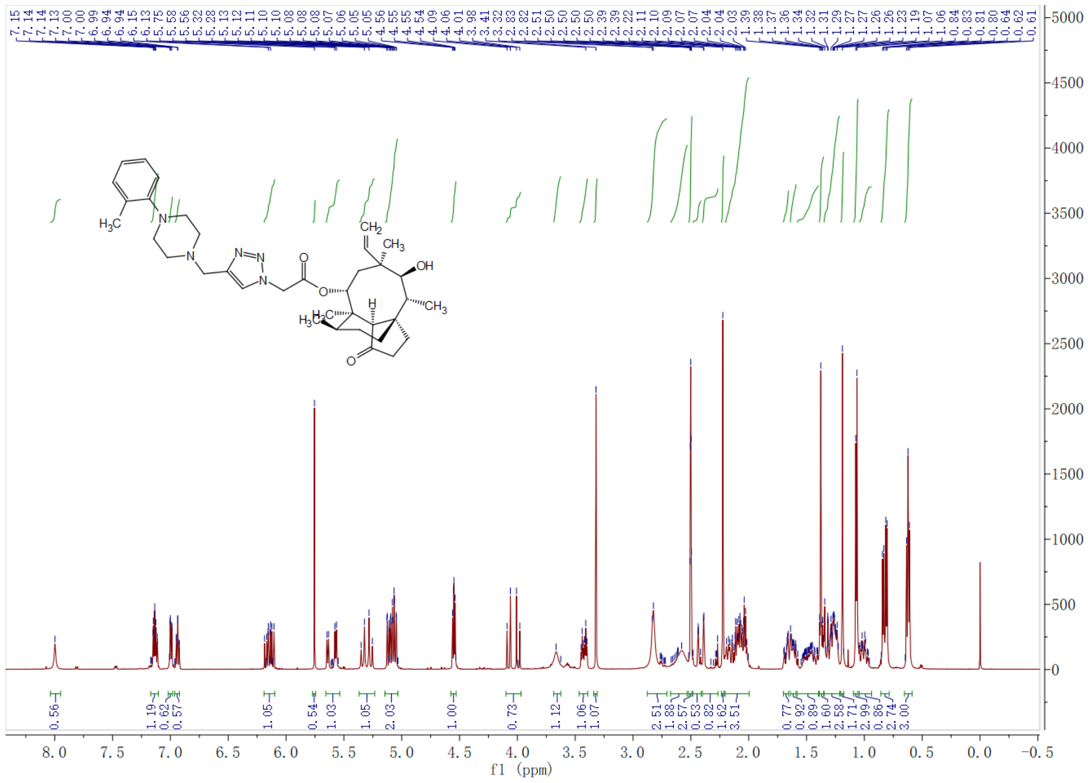

Figure SI 3-1. ^{1}H-NMR spectrum (DMSO-*d6*, 600 MHz) of compound **50.**

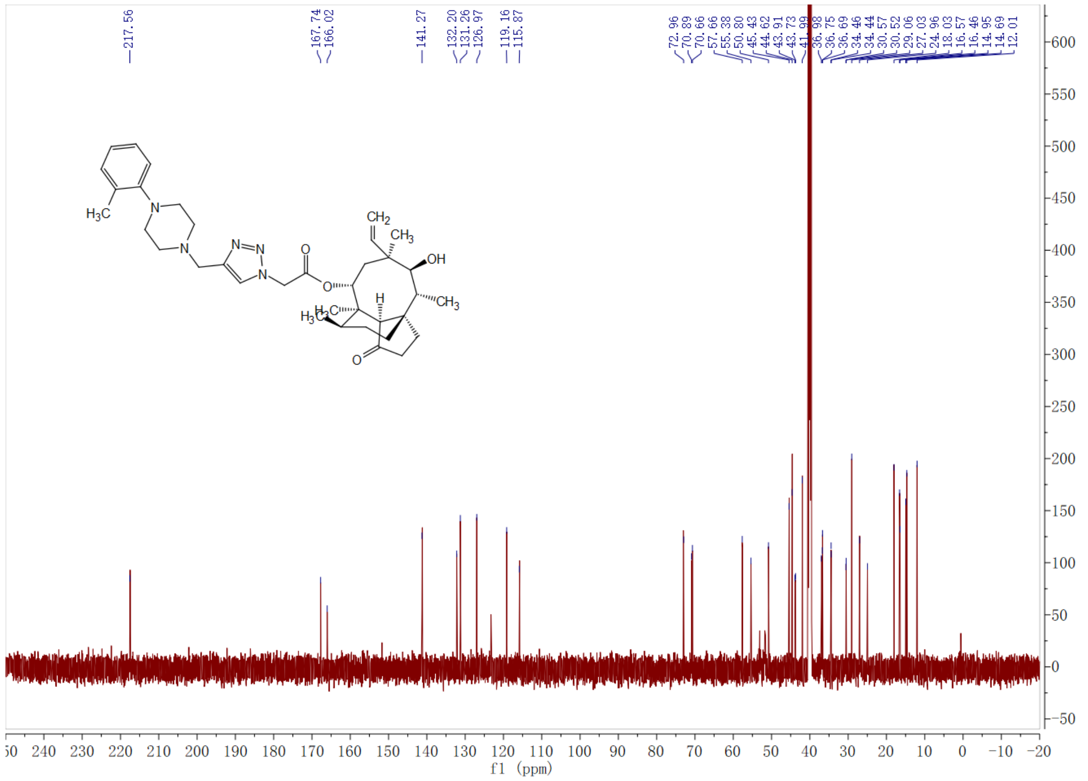

Figure SI 3-2. ^{13}C-NMR spectrum (DMSO-*d6*, 151 MHz) of compound **50.**

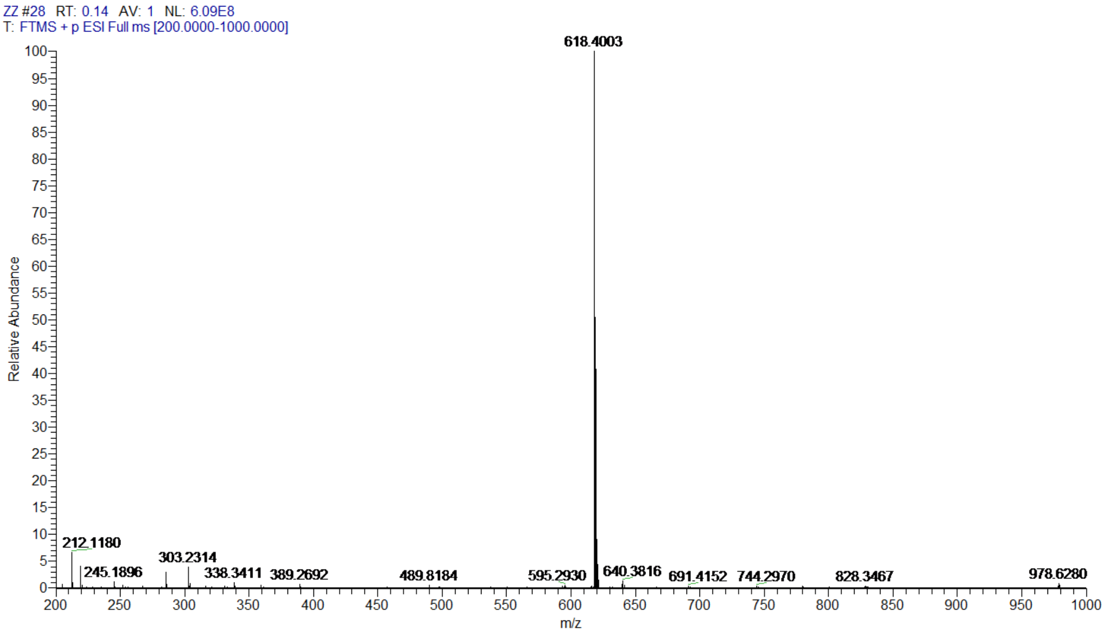

Figure SI 3-3. HR Mass spectrum (ESI) of compound **50.**

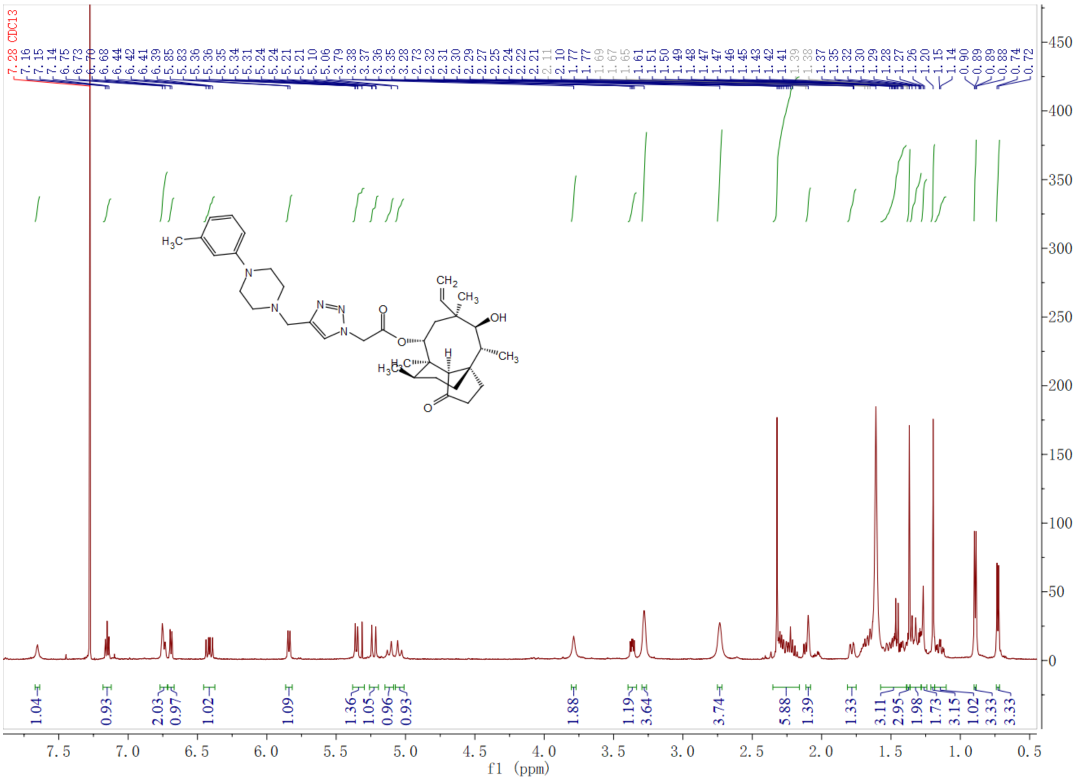

Figure SI 4-1. ^{1}H-NMR spectrum (DMSO-*d6*, 600 MHz) of compound **51.**

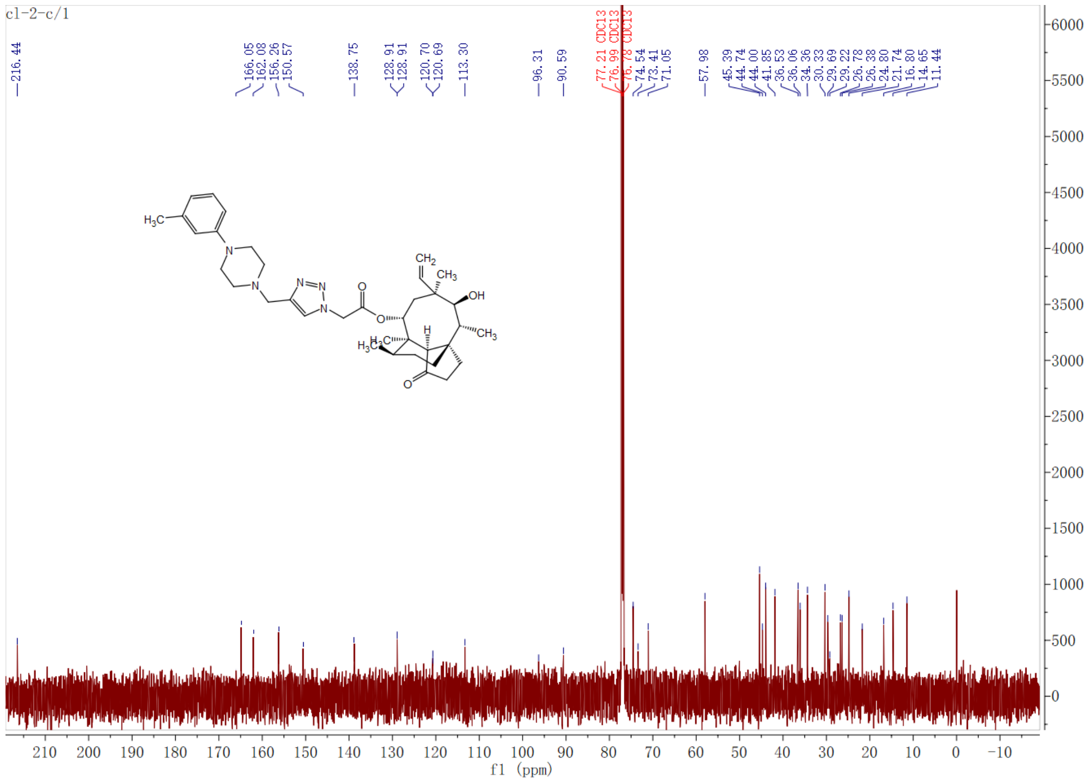

Figure SI 4-2. ^{13}C-NMR spectrum (DMSO-*d6*, 151 MHz) of compound **51.**

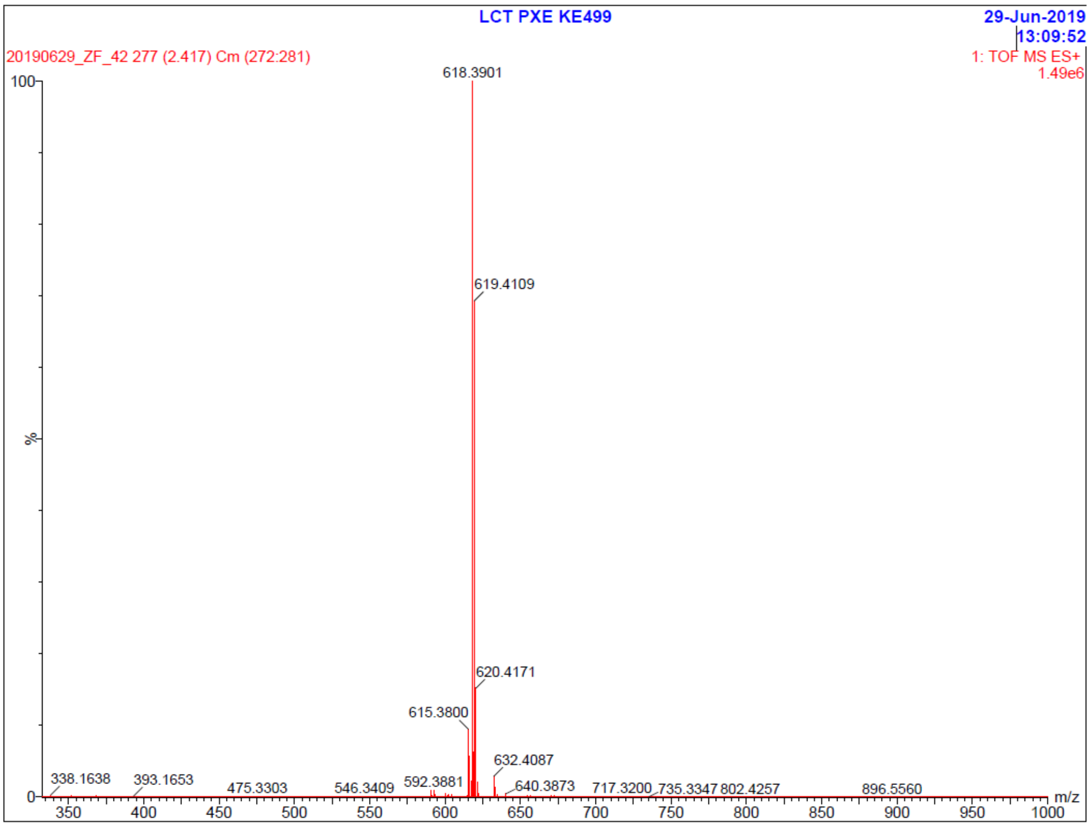

Figure SI 4-3. HR Mass spectrum (ESI) of compound **51.**

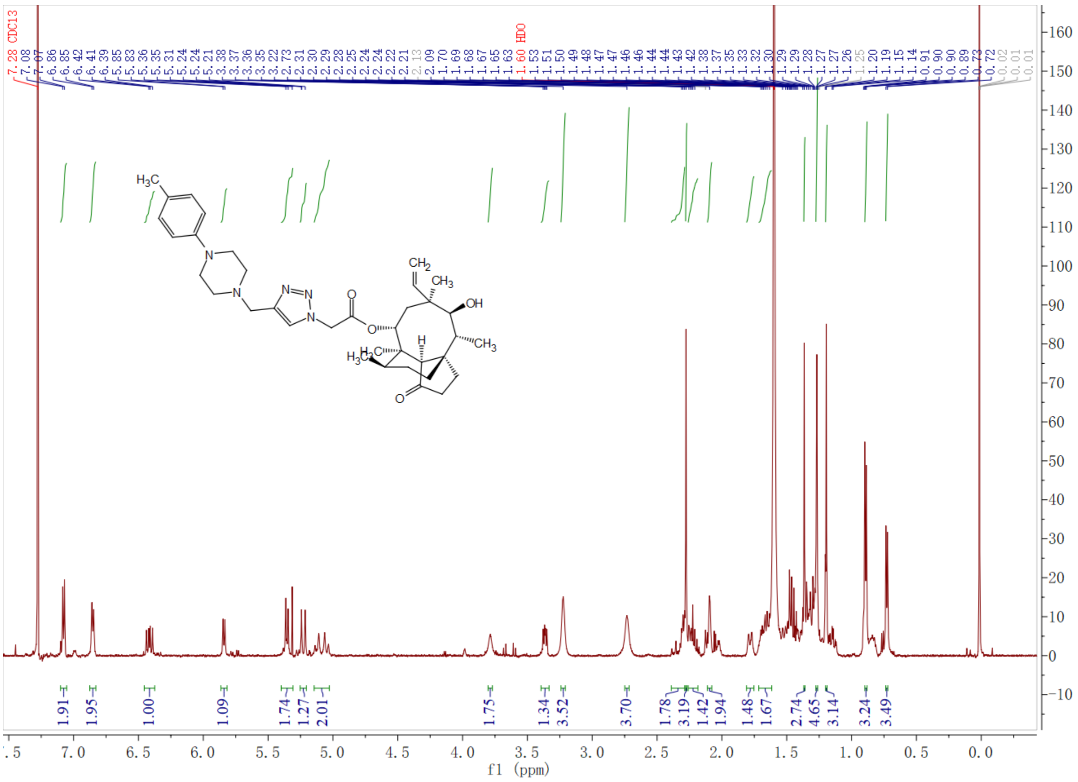

Figure SI 5-1. ^{1}H-NMR spectrum (Chloroform-*d*, 600 MHz) of compound **52.**

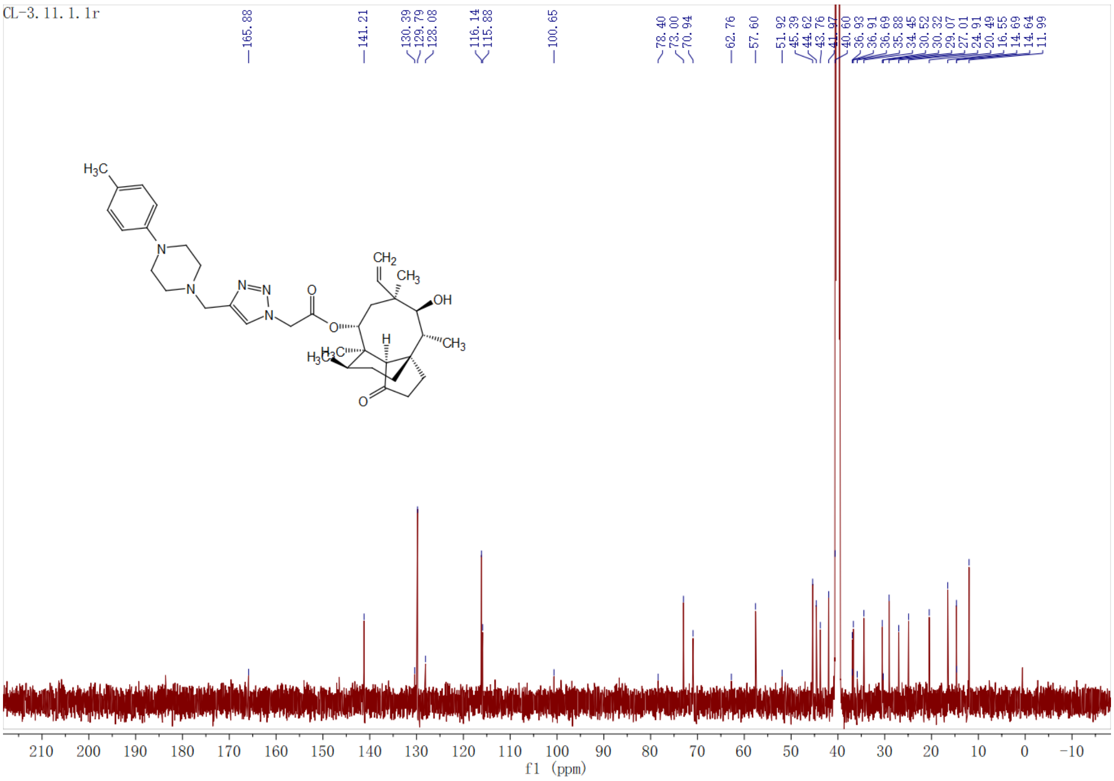

Figure SI 5-2. ^{13}C-NMR spectrum (DMSO-*d6*, 151 MHz) of compound **52.**

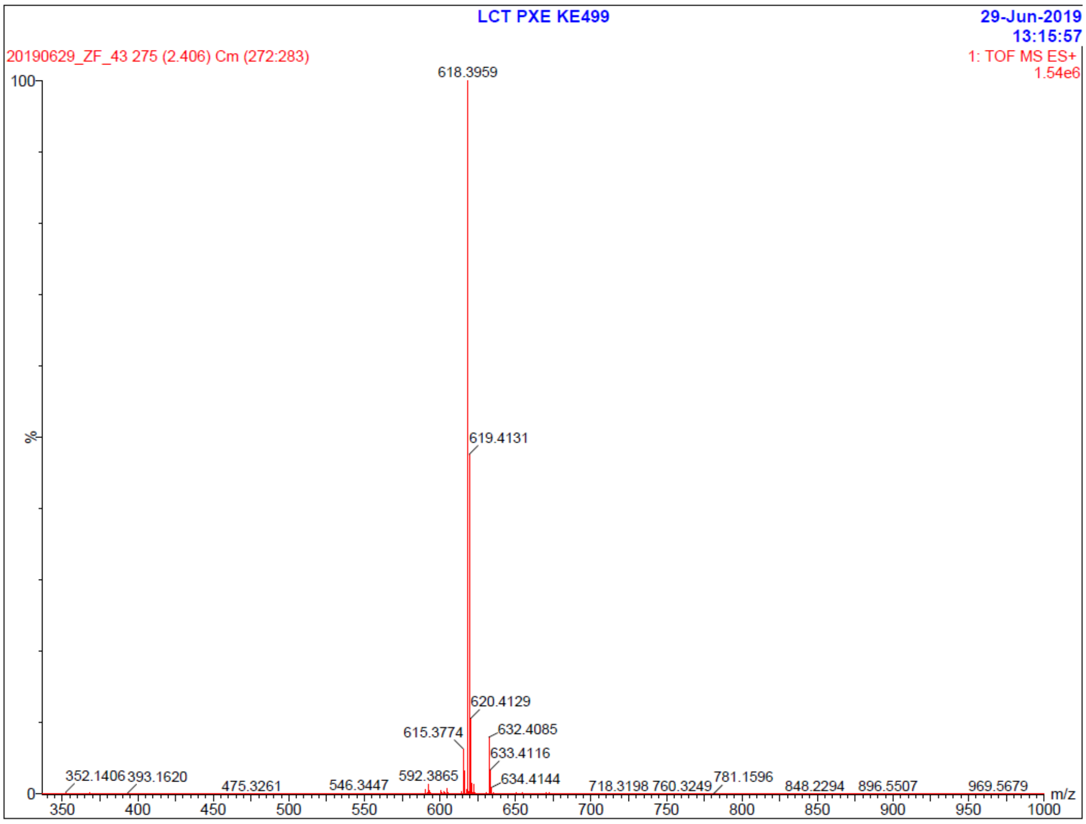

Figure SI 5-3. HR Mass spectrum (ESI) of compound **52.**

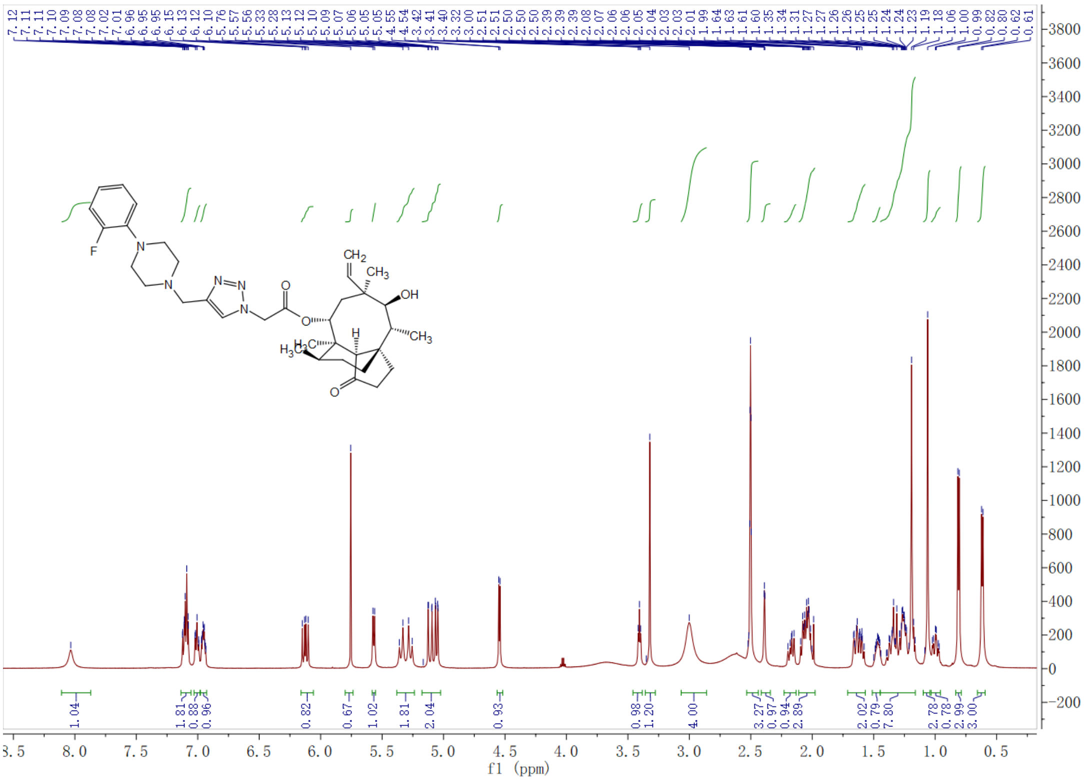

Figure SI 6-1. ^{1}H-NMR spectrum (DMSO-*d6*, 600 MHz) of compound **53.**

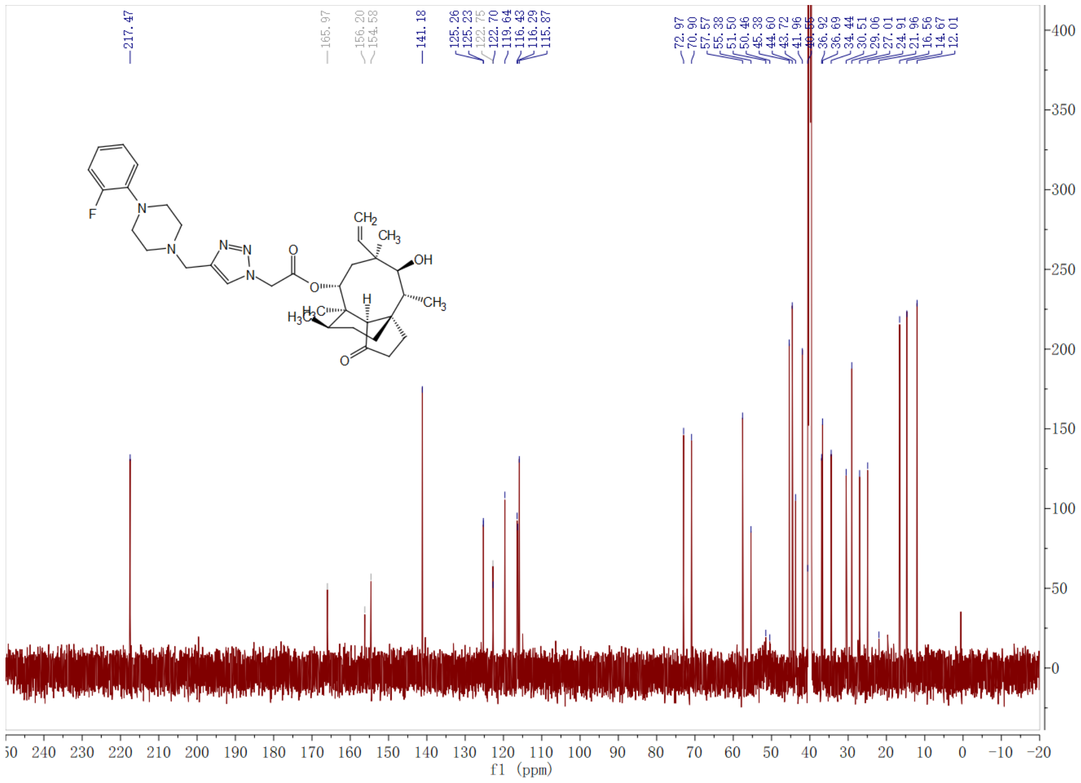

Figure SI 6-2. ^{13}C-NMR spectrum (DMSO-*d6*, 151 MHz) of compound **53.**

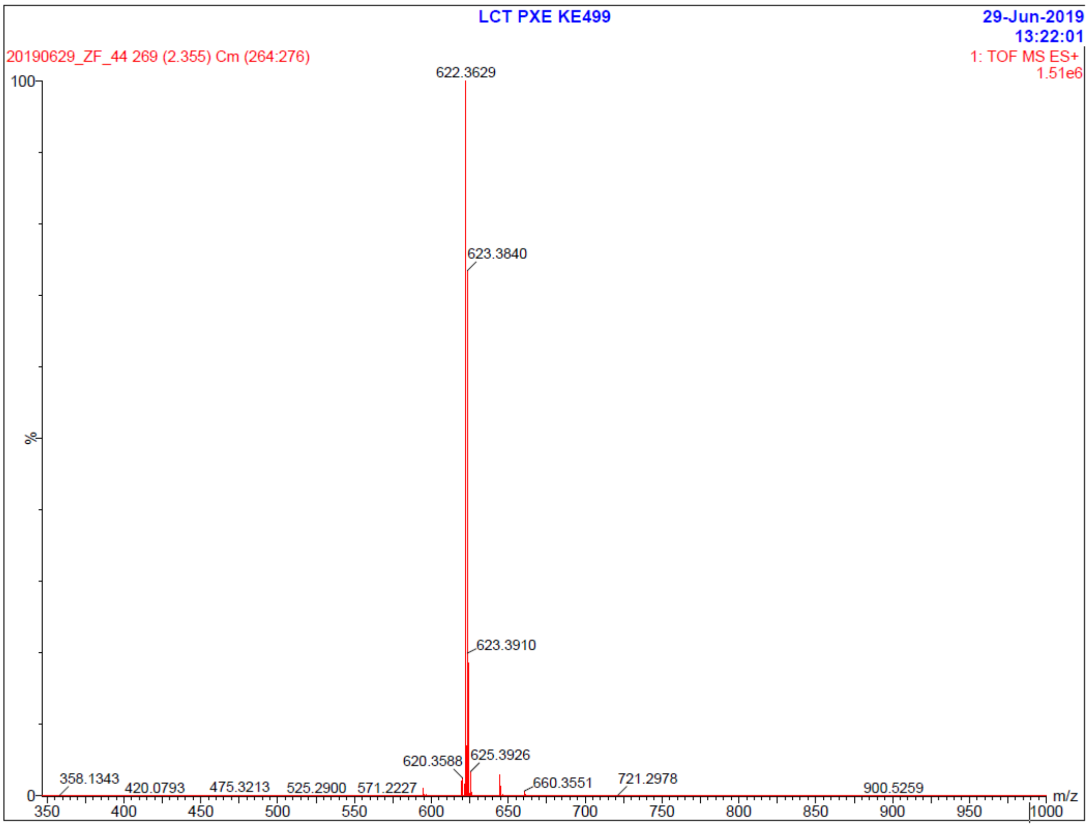

Figure SI 6-3. HR Mass spectrum (ESI) of compound **53.**

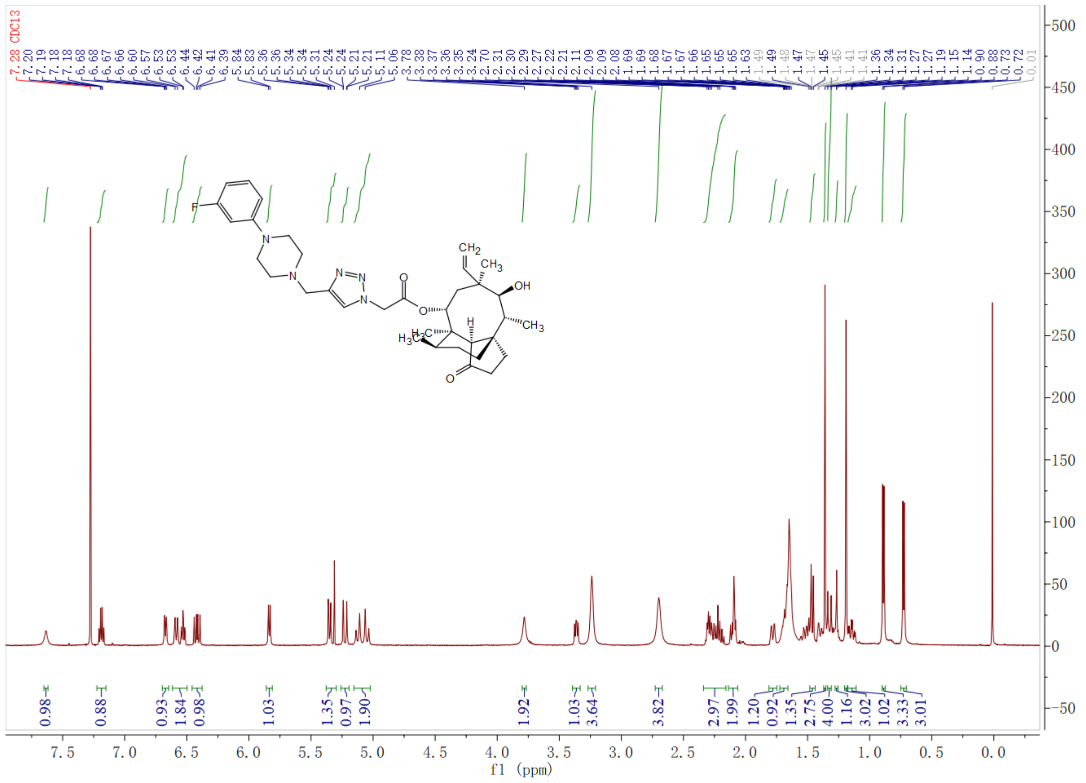

Figure SI 7-1. ^{1}H-NMR spectrum (Chloroform-*d*, 600 MHz) of compound **54.**

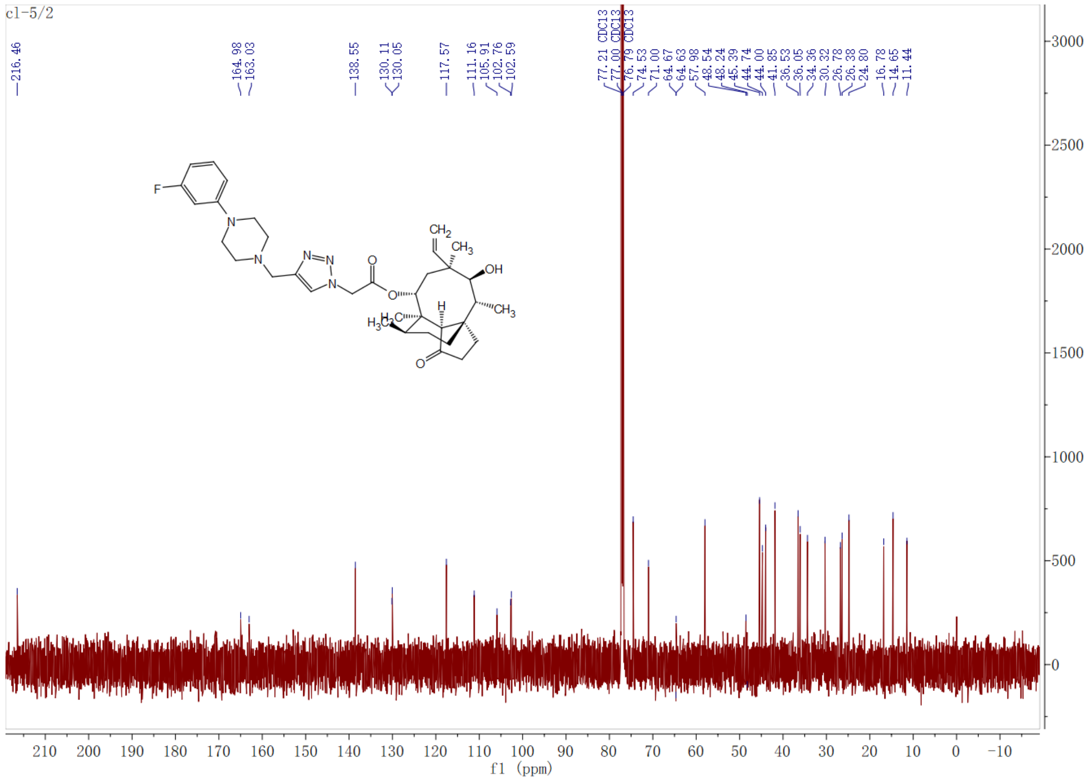

Figure SI 7-2. ^{13}C-NMR spectrum (Chloroform-*d*, 151 MHz) of compound **54.**

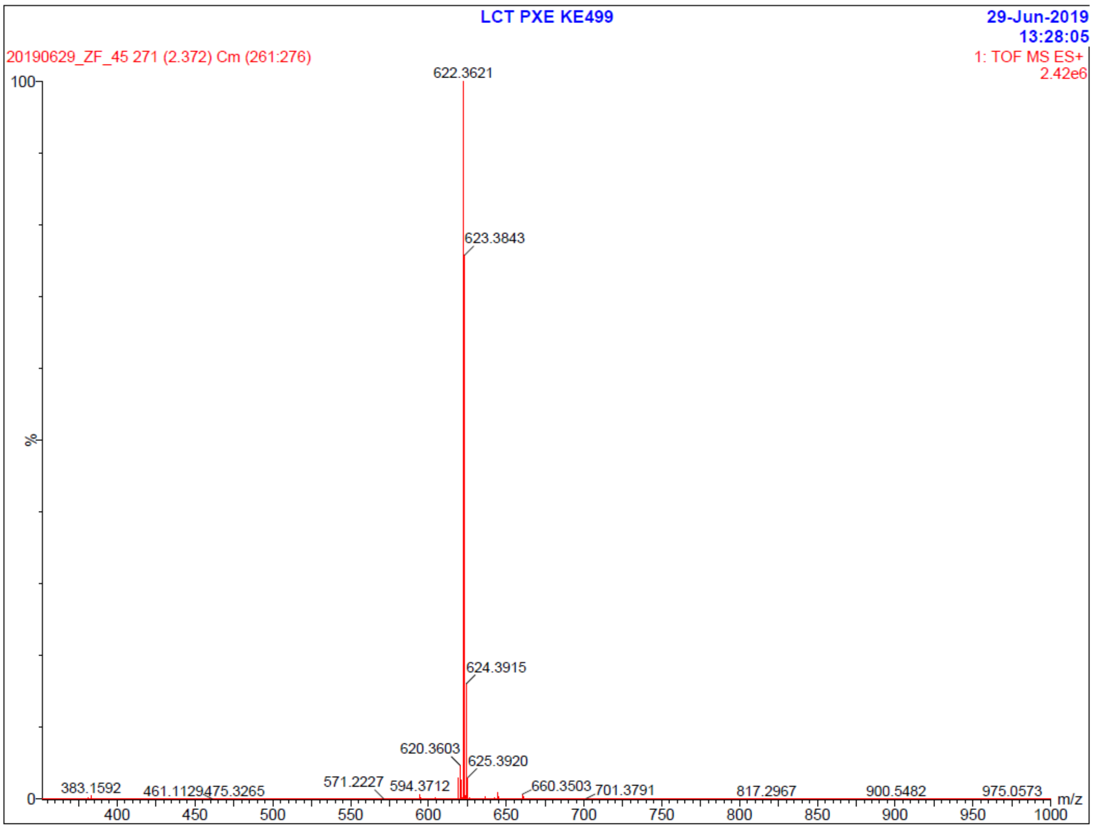

Figure SI 7-3. HR Mass spectrum (ESI) of compound **54.**

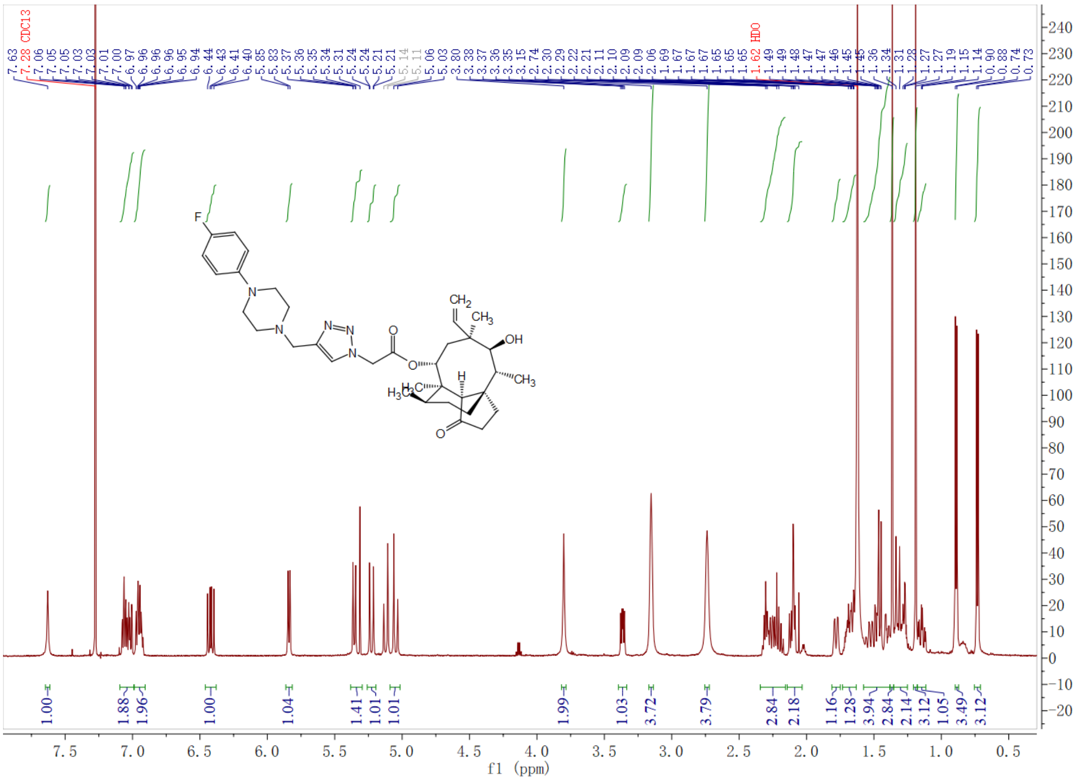

Figure SI 8-1. ^{1}H-NMR spectrum (Chloroform-d, 600 MHz) of compound **55.**

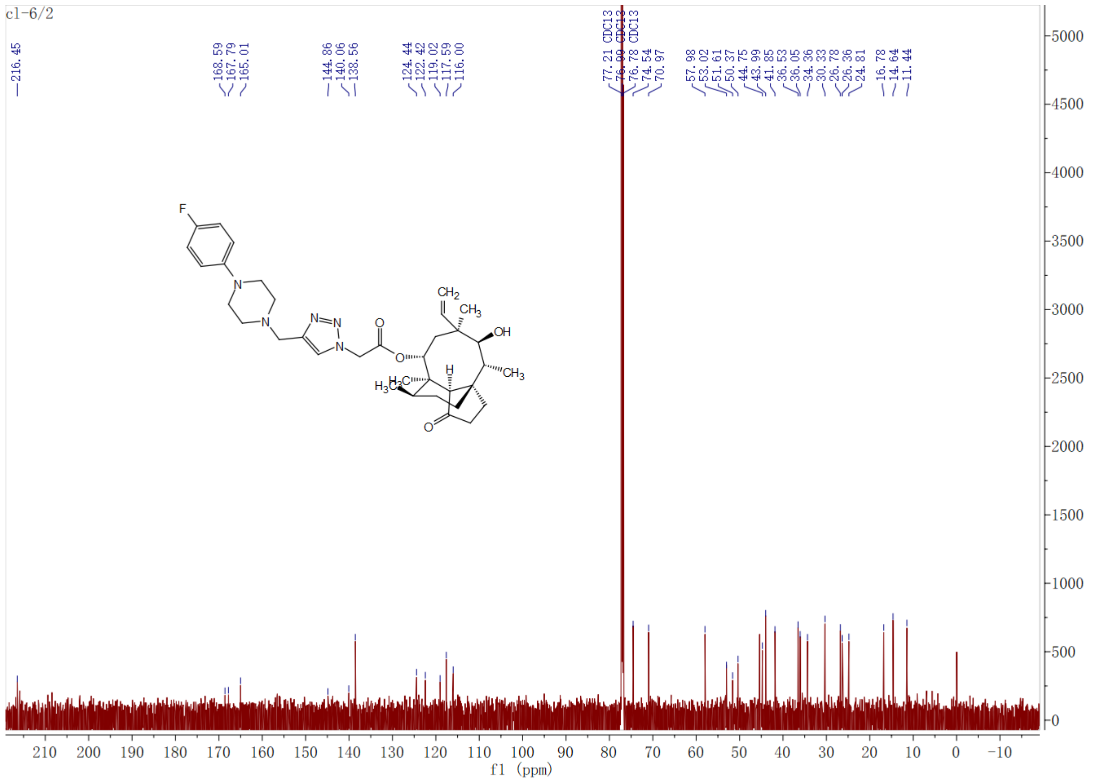

Figure SI 8-2. ^{13}C-NMR spectrum (Chloroform-*d*, 151 MHz) of compound **55.**

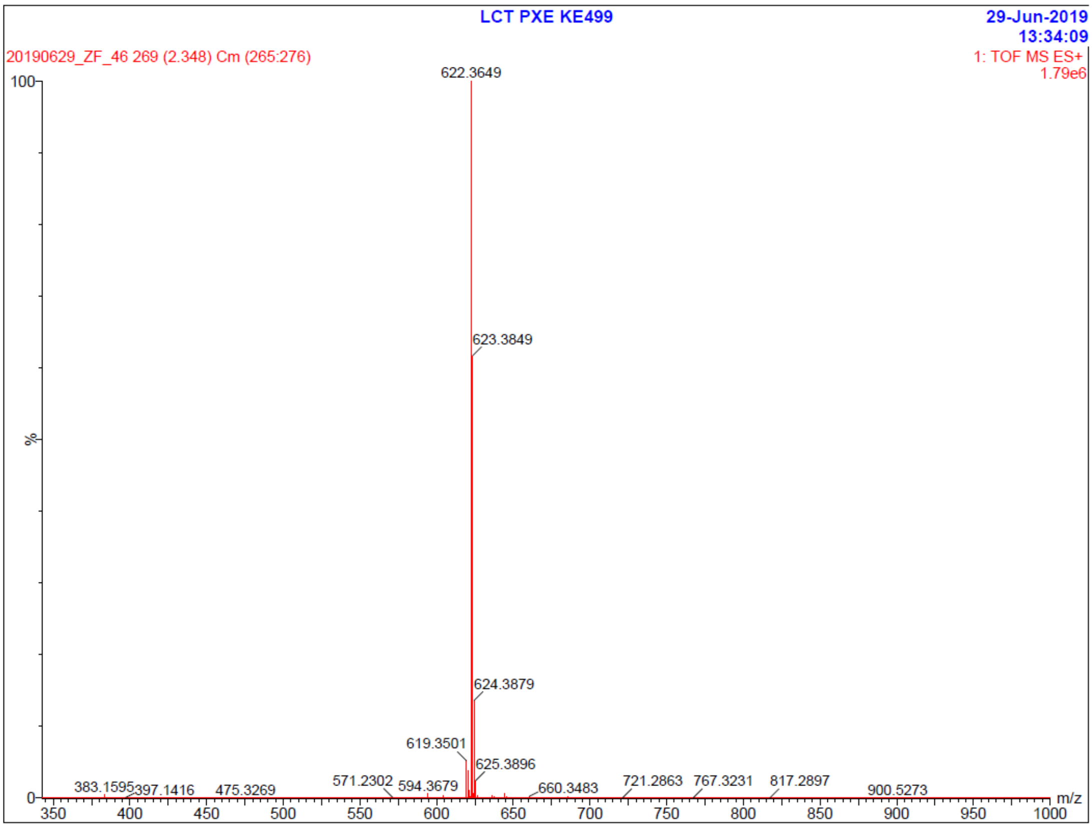

Figure SI 8-3. HR Mass spectrum (ESI) of compound **55.**

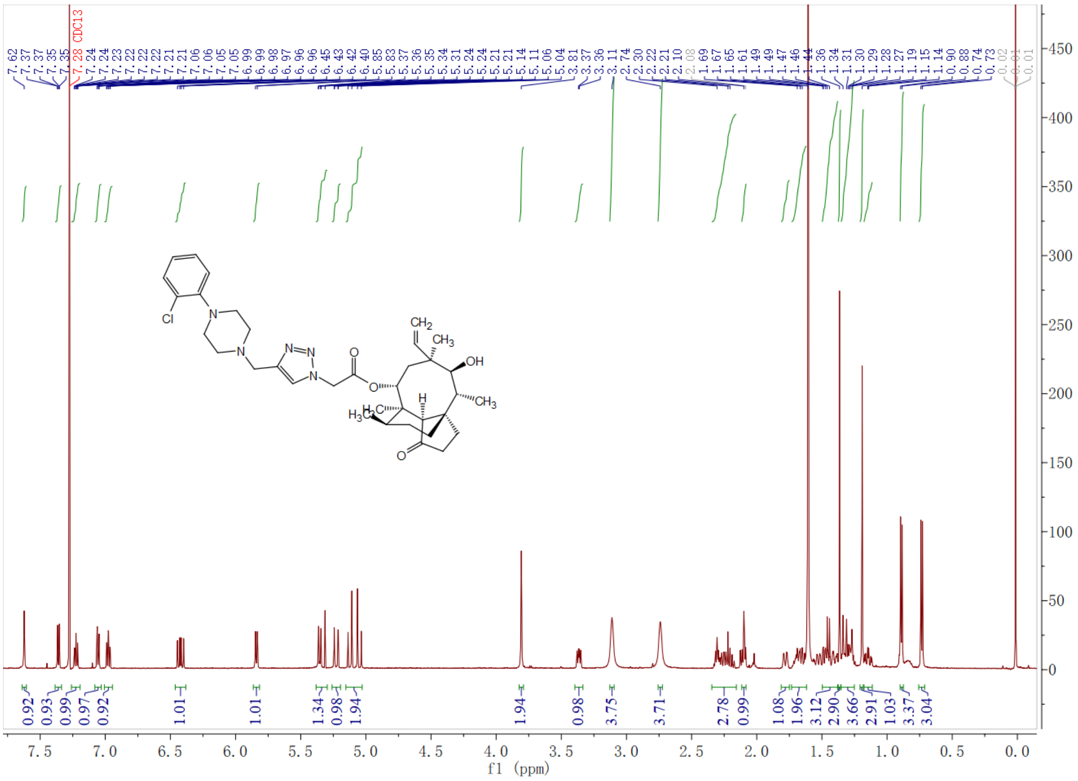

Figure SI 9-1. ^{1}H-NMR spectrum (Chloroform-*d*, 600 MHz) of compound **56.**

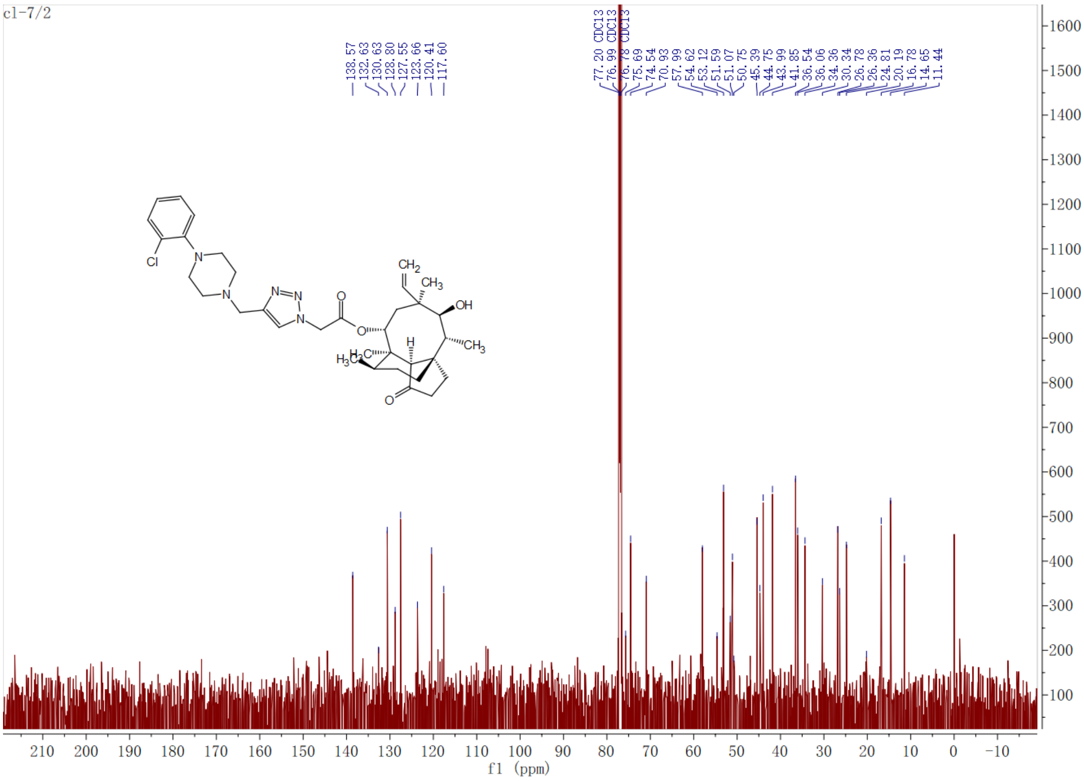

Figure SI 4-2. ^{13}C-NMR spectrum (Chloroform-*d*, 151 MHz) of compound **56.**

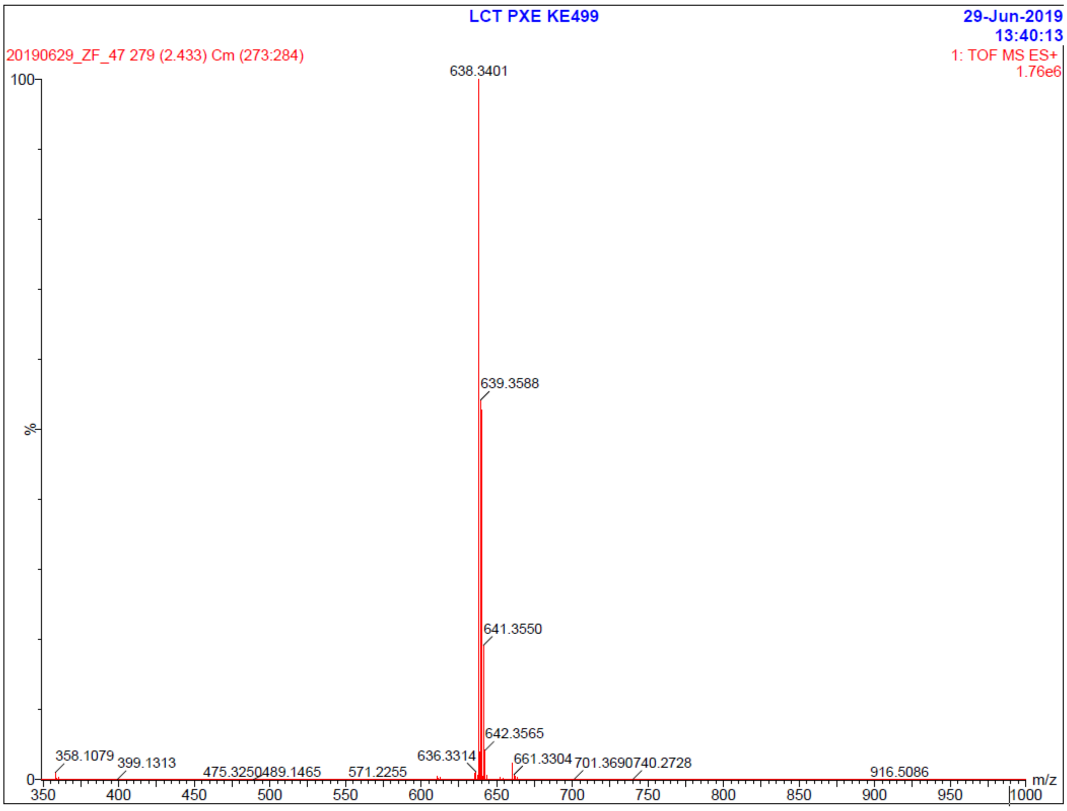

Figure SI 9-3. HR Mass spectrum (ESI) of compound **56.**

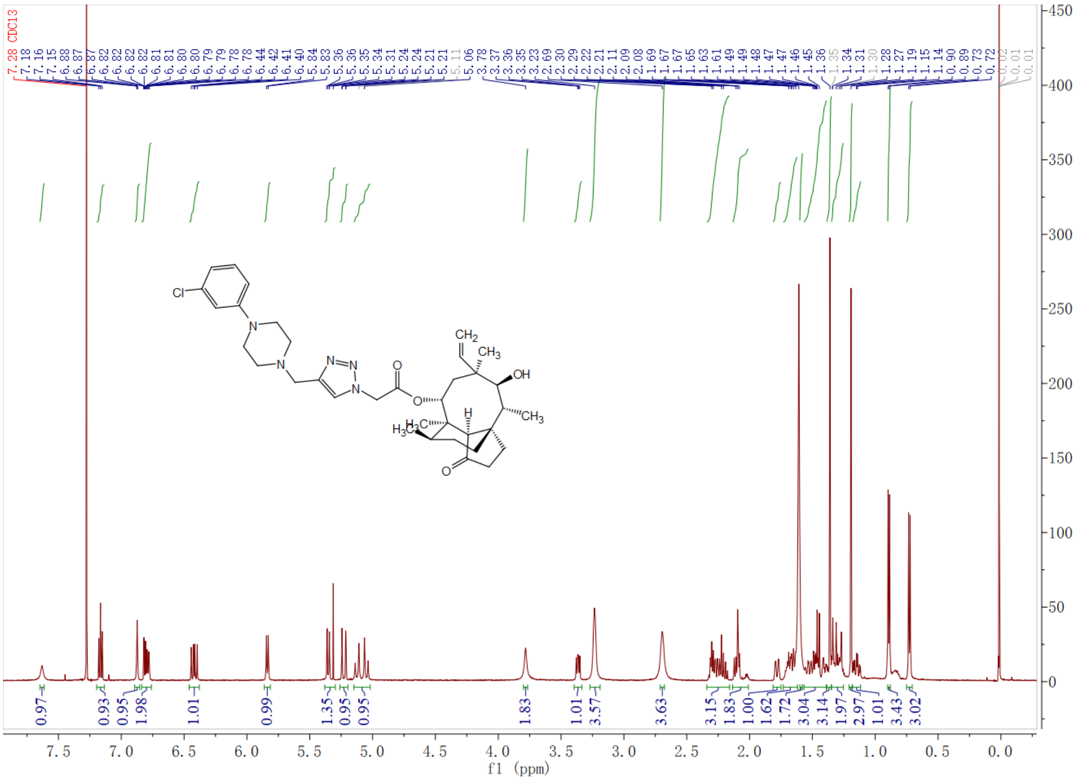

Figure SI 10-1. ^{1}H-NMR spectrum (Chloroform-d, 600 MHz) of compound **57.**

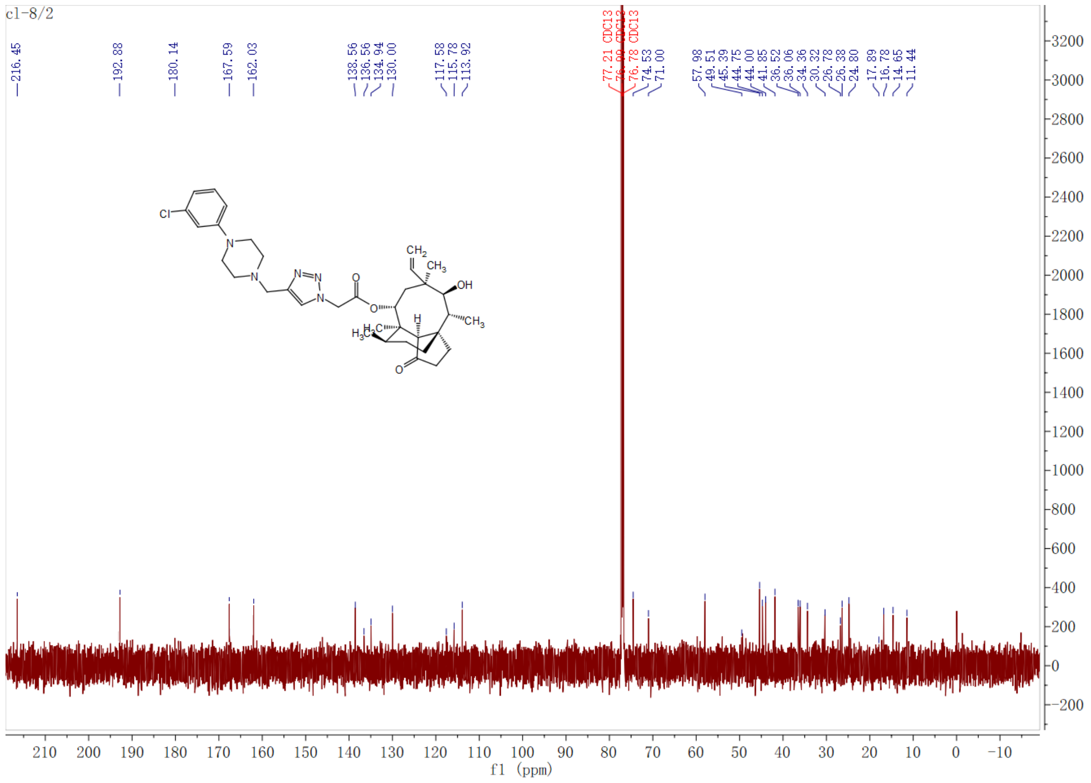

Figure SI 10-2. ^{13}C-NMR spectrum (Chloroform-*d*, 151 MHz) of compound **57.**

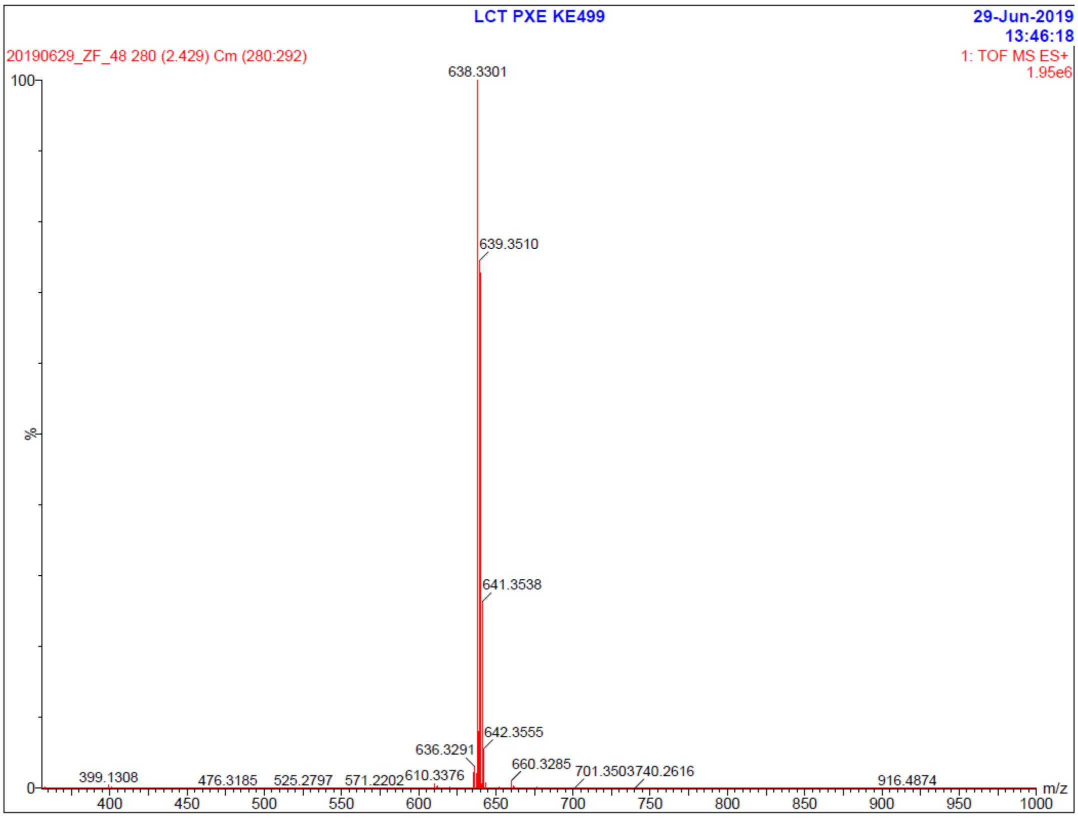

Figure SI 10-3. HR Mass spectrum (ESI) of compound **57.**

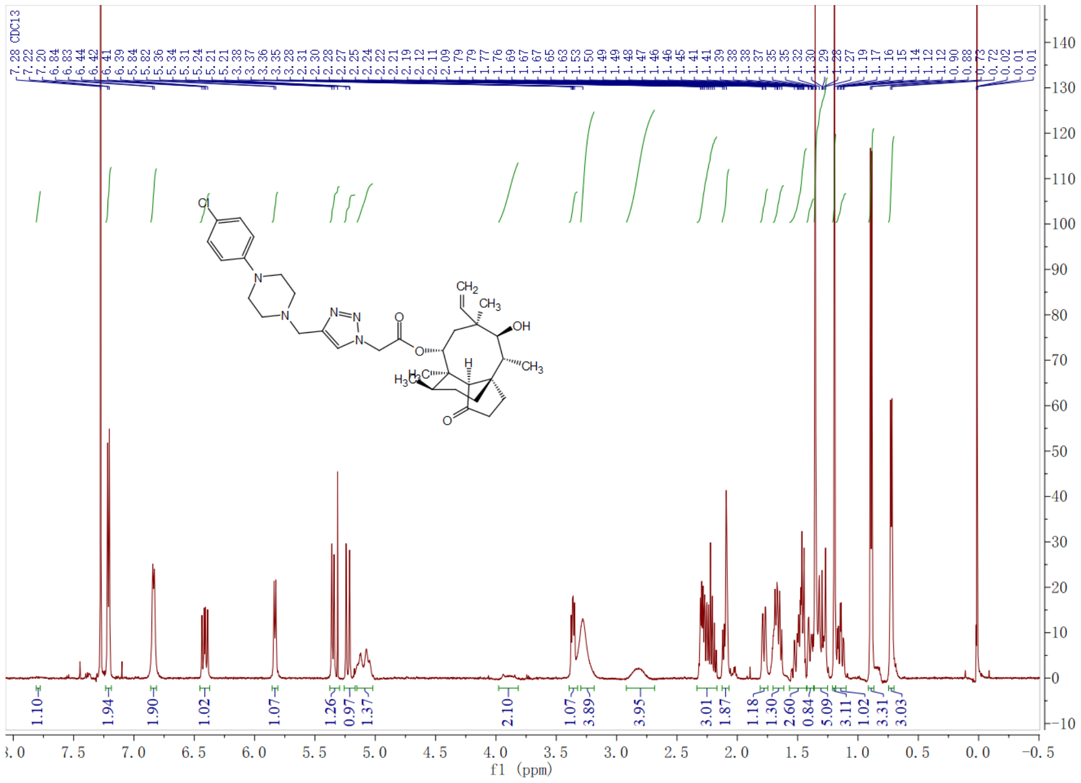

Figure SI 11-1. ^{1}H-NMR spectrum (Chloroform-*d*, 600 MHz) of compound **58.**

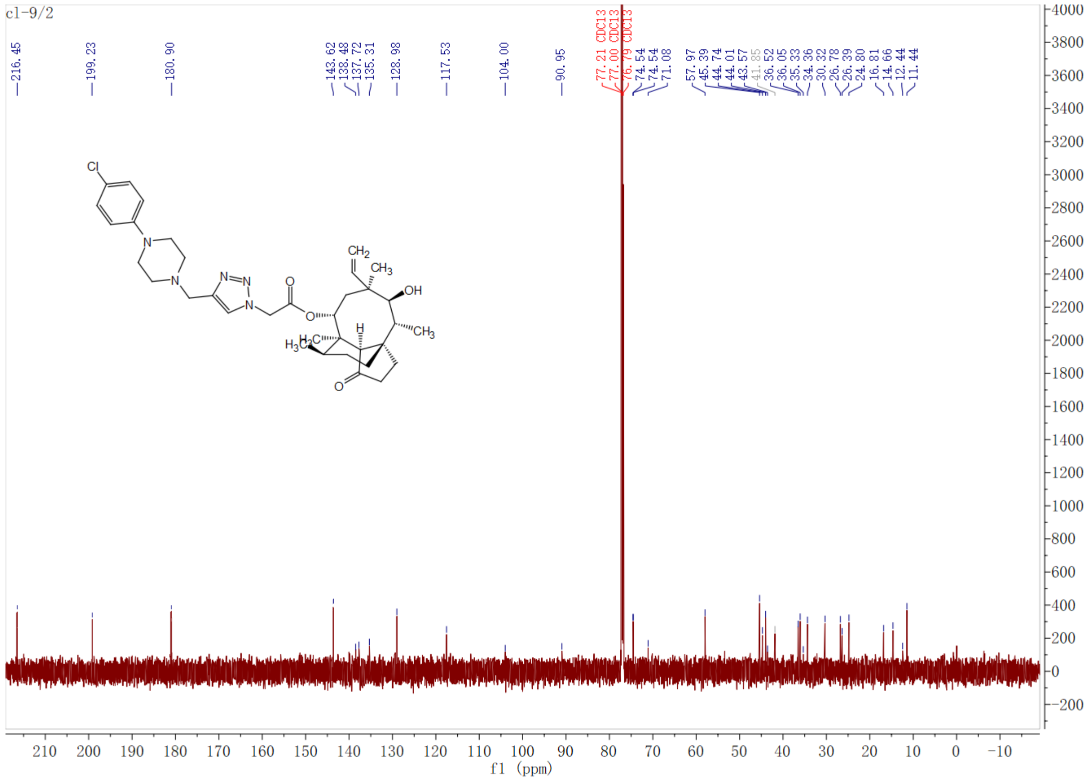

Figure SI 11-2. ^{13}C-NMR spectrum (Chloroform-*d*, 151 MHz) of compound **58.**

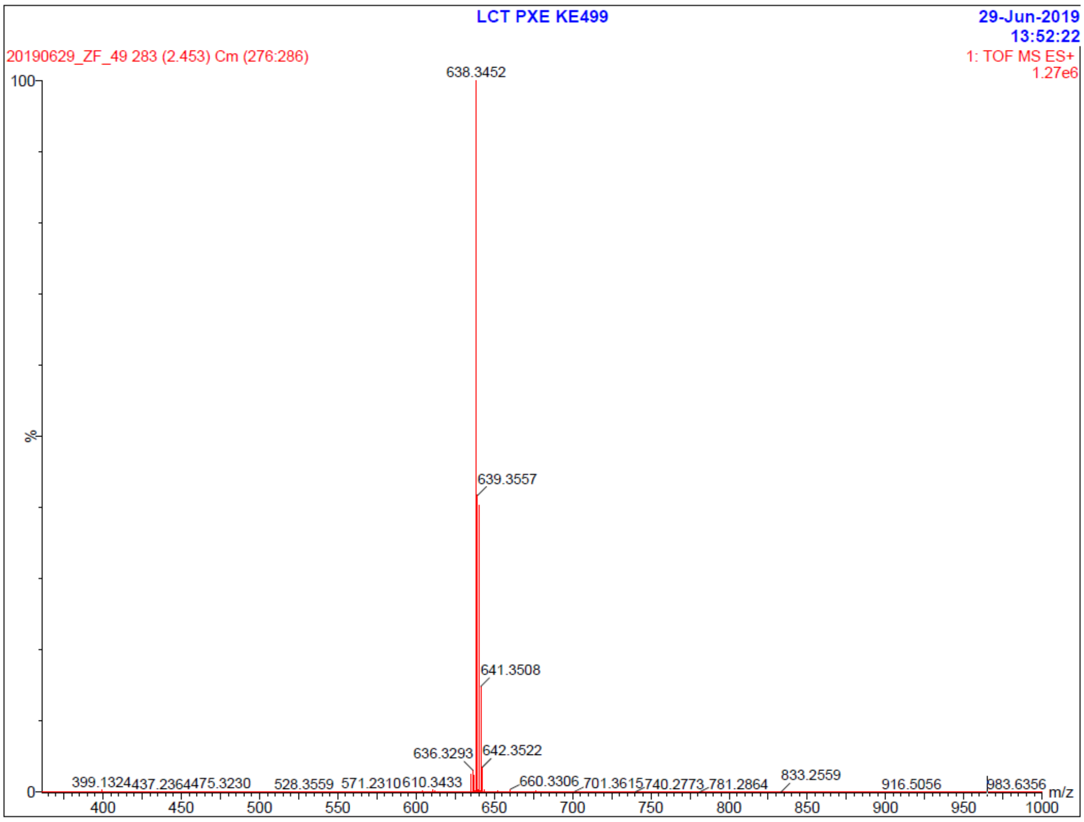

Figure SI 11-3. HR Mass spectrum (ESI) of compound **58.**

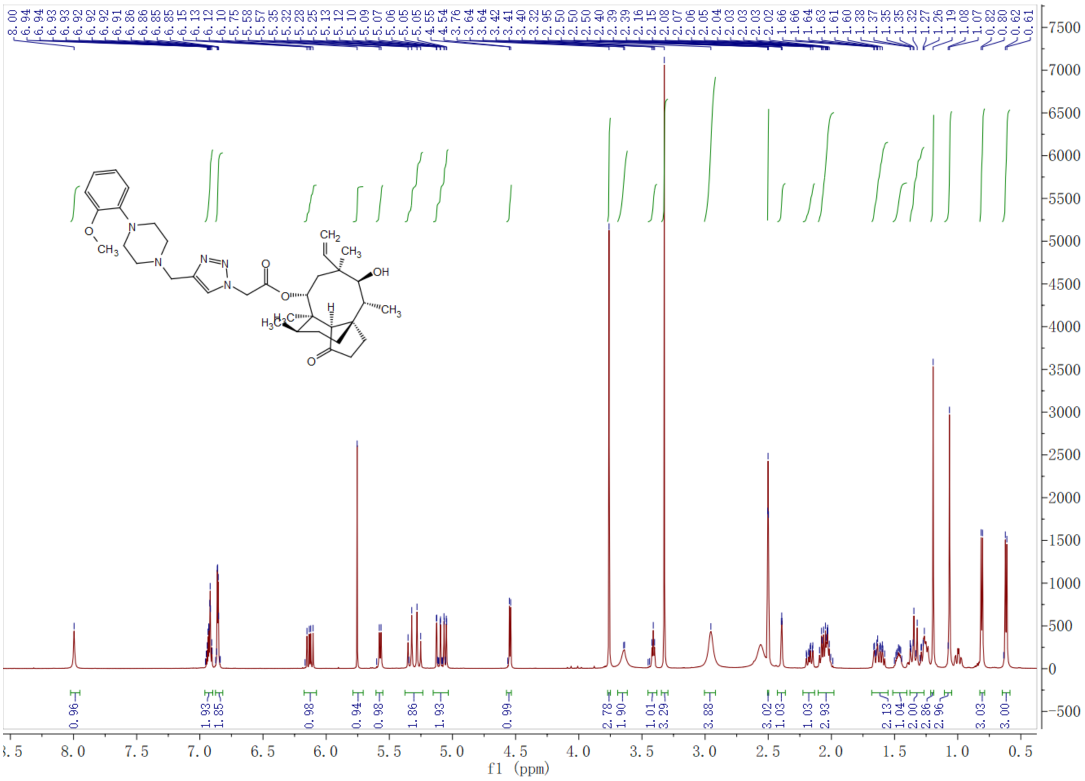

Figure SI 12-1. ^{1}H-NMR spectrum (DMSO-*d6*, 600 MHz) of compound **59.**

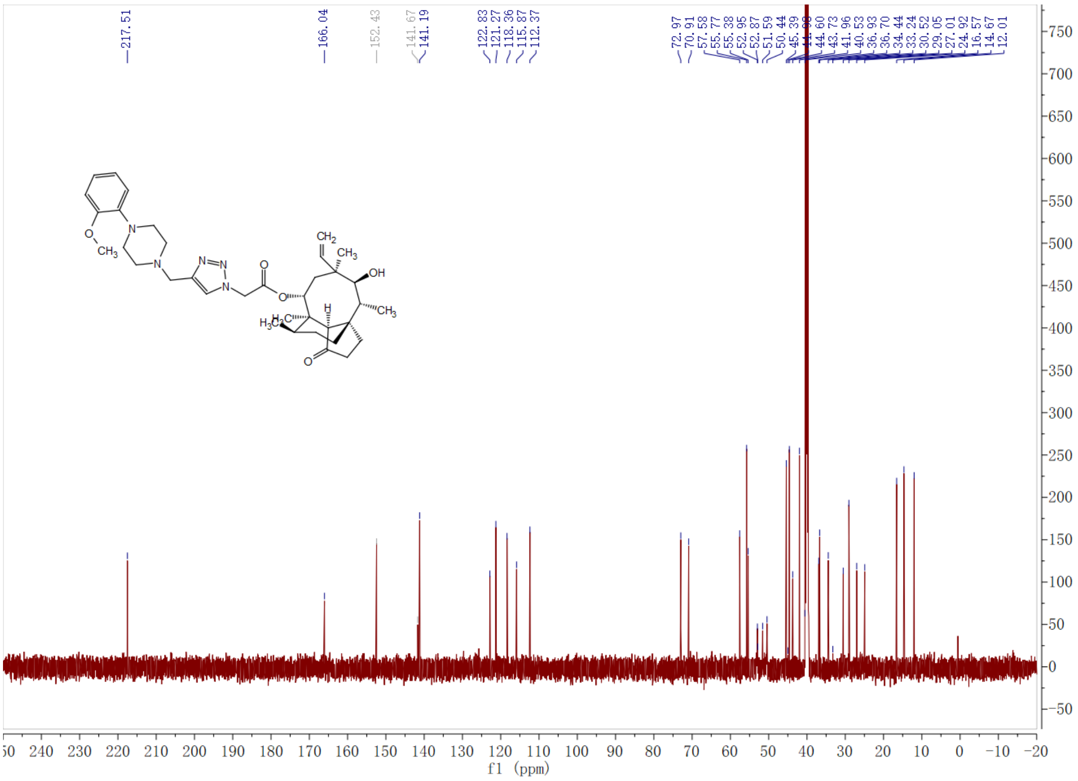

Figure SI 12-2. ^{13}C-NMR spectrum (DMSO-*d6*, 151 MHz) of compound **59.**

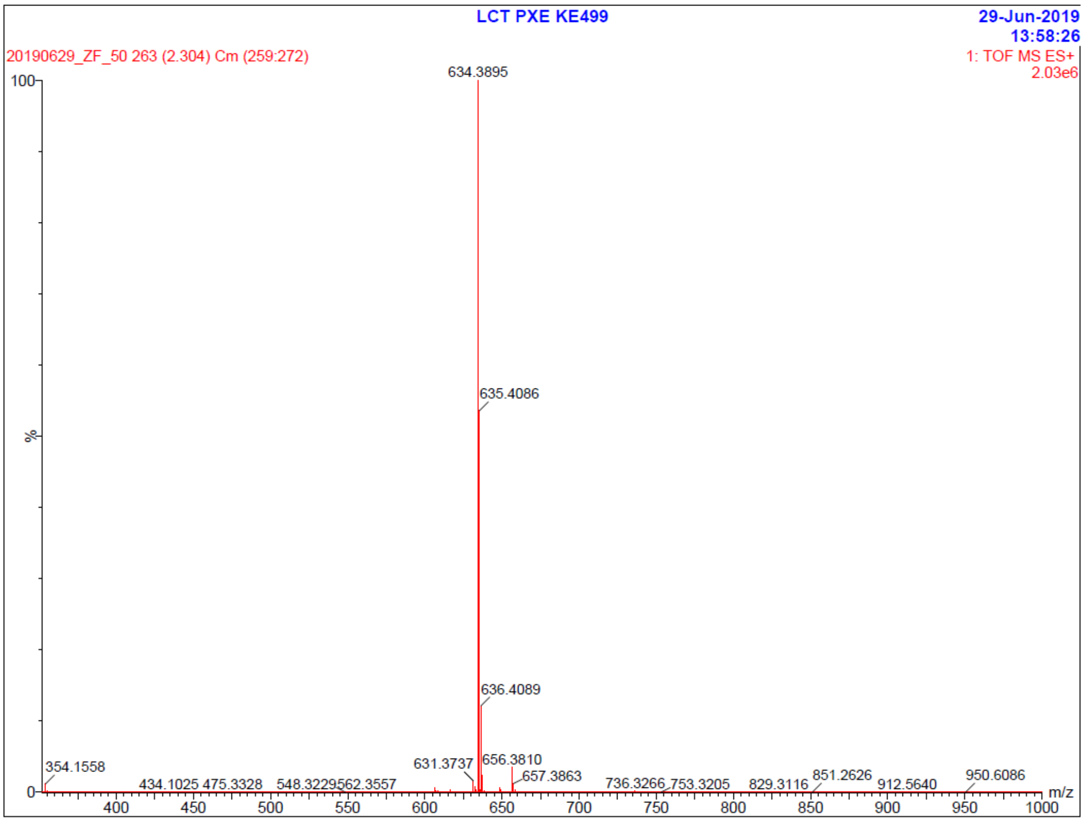

Figure SI 12-3. HR Mass spectrum (ESI) of compound **59.**

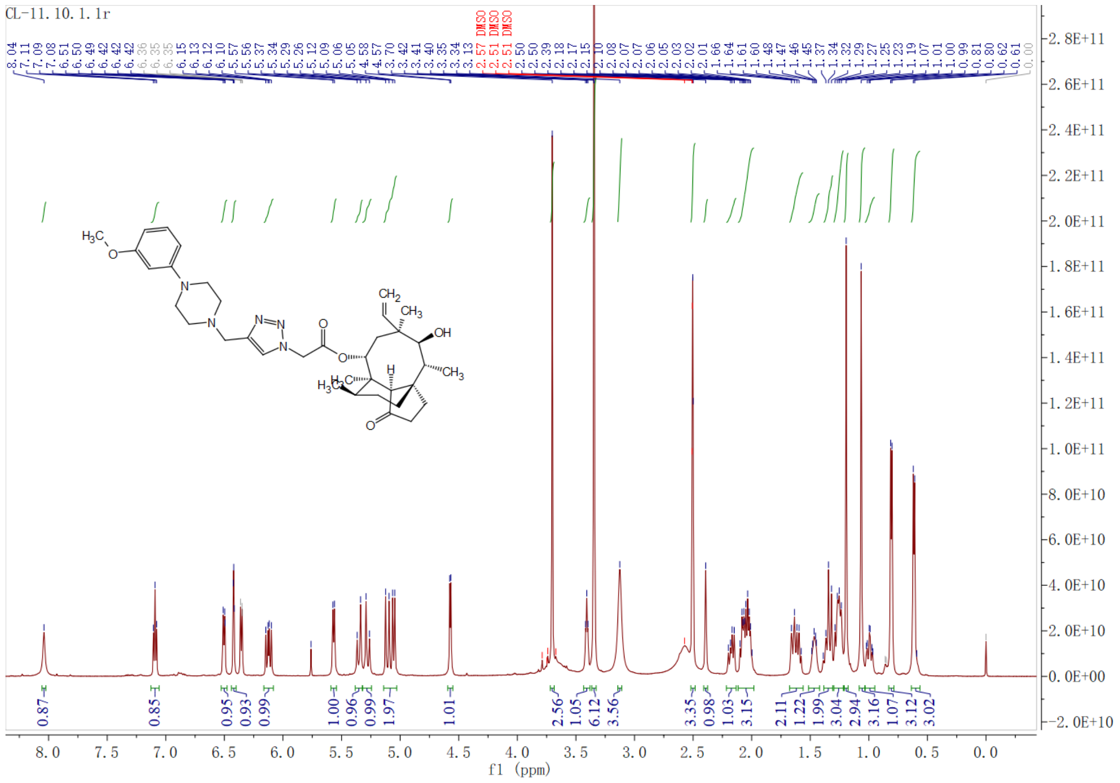

Figure SI 13-1. ^{1}H-NMR spectrum (DMSO-*d6*, 600 MHz) of compound **60.**

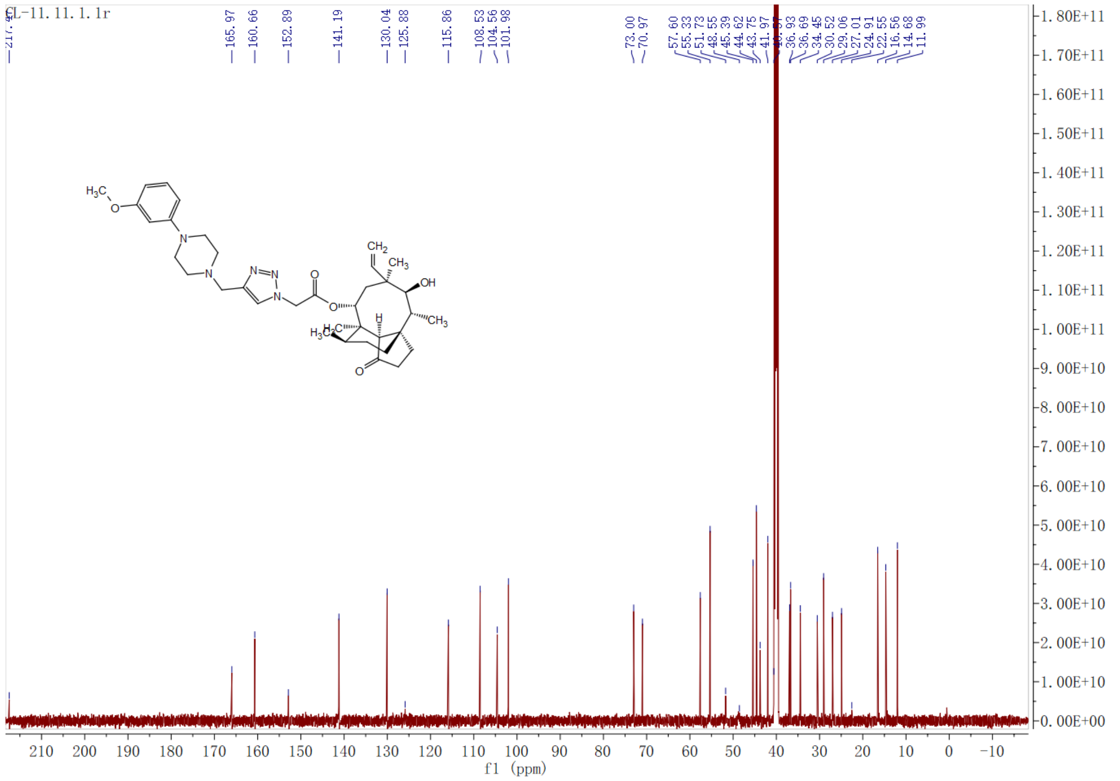

Figure SI 13-2. ^{13}C-NMR spectrum (DMSO-*d6*, 151 MHz) of compound **60.**

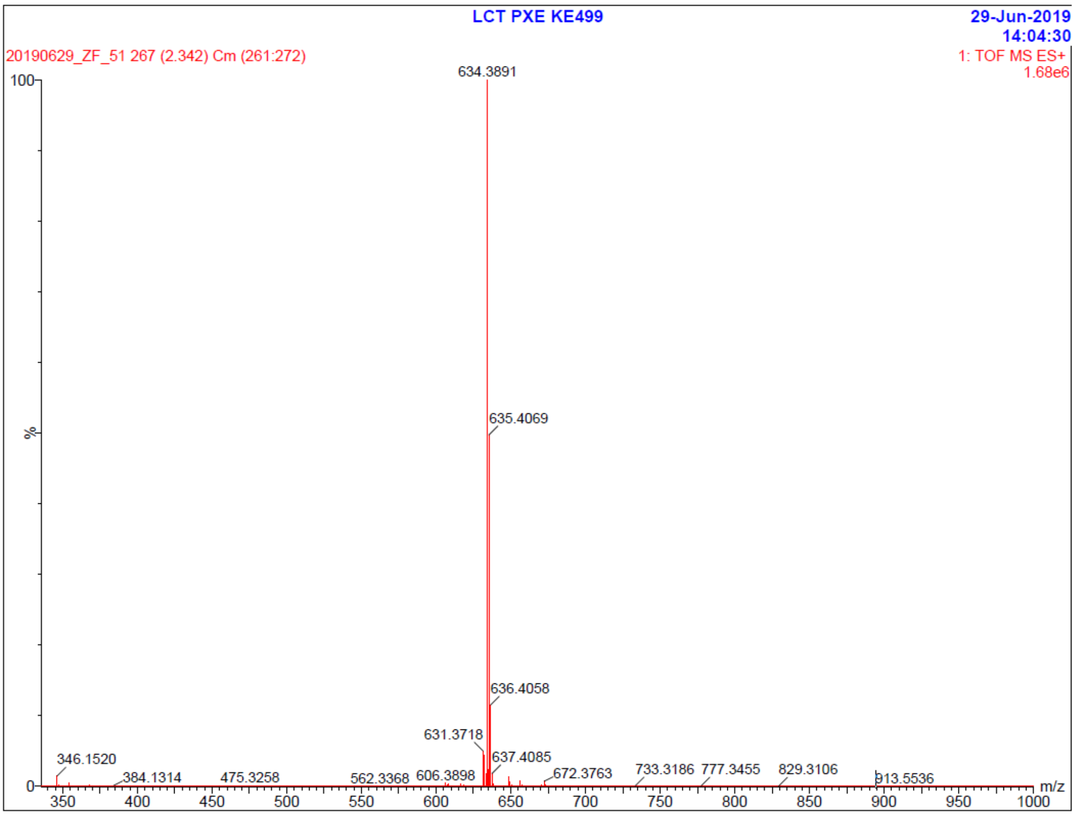

Figure SI 13-3. HR Mass spectrum (ESI) of compound **60.**

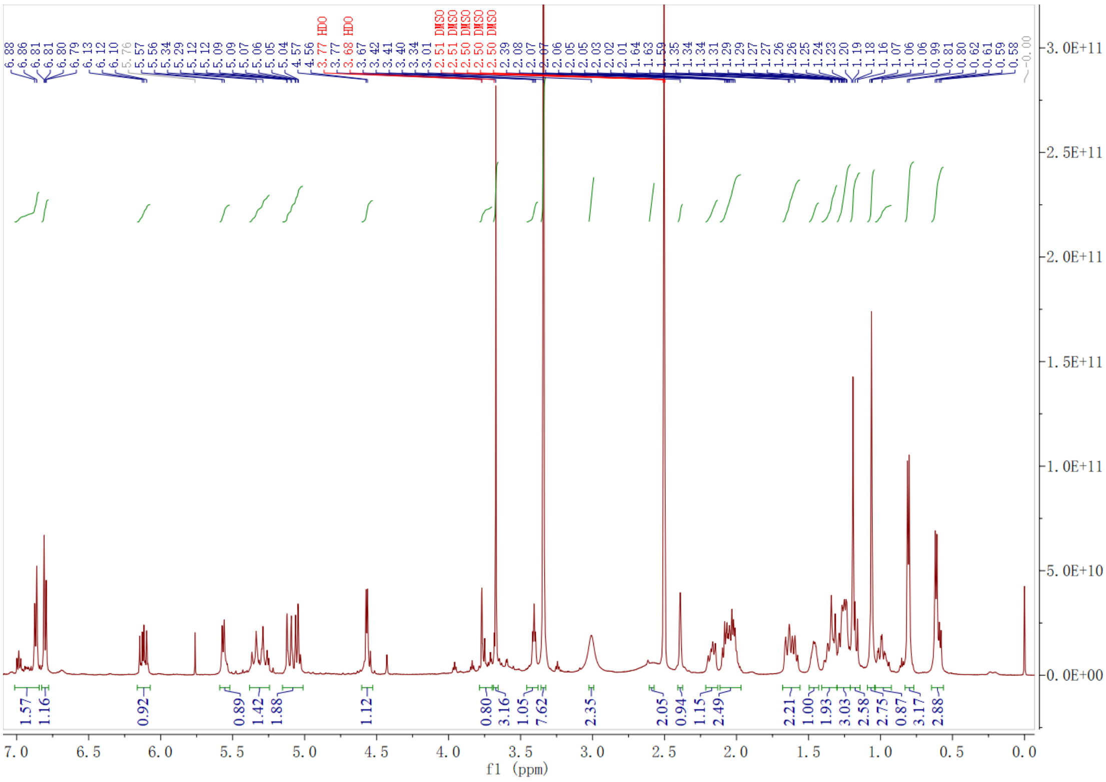

Figure SI 14-1. ^{1}H-NMR spectrum (DMSO-*d6*, 600 MHz) of compound **61.**

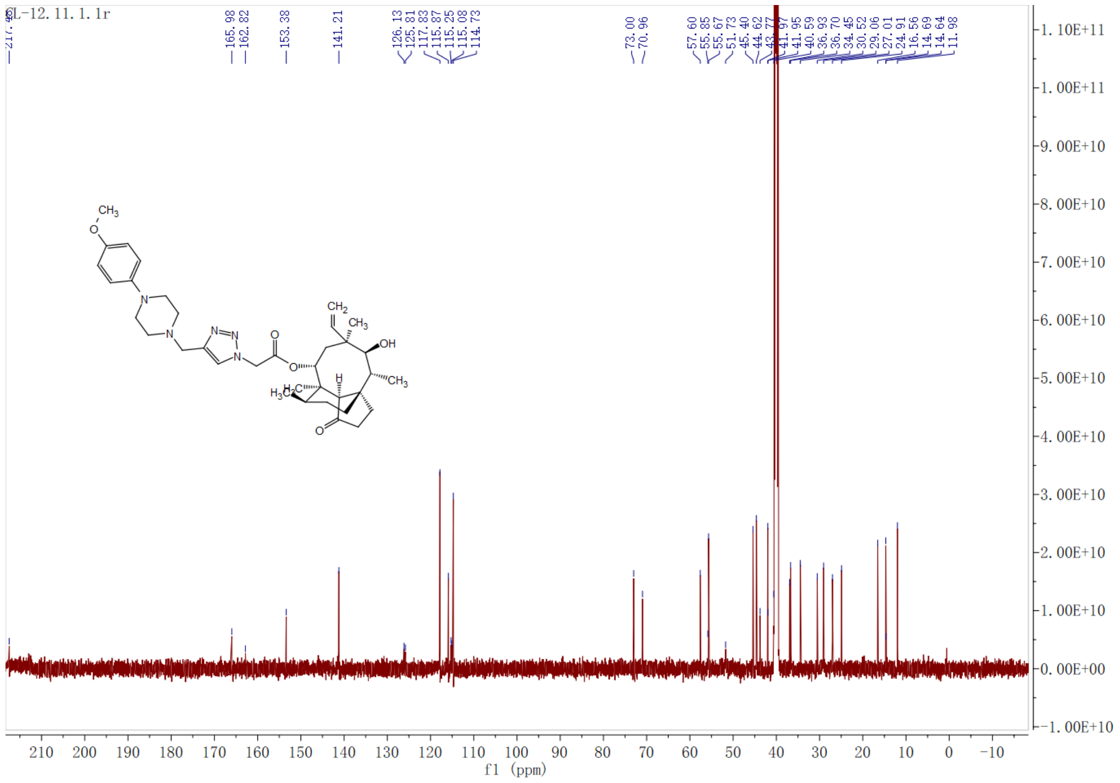

Figure SI 14-2. ^{13}C-NMR spectrum (DMSO-*d6*, 151 MHz) of compound **61.**

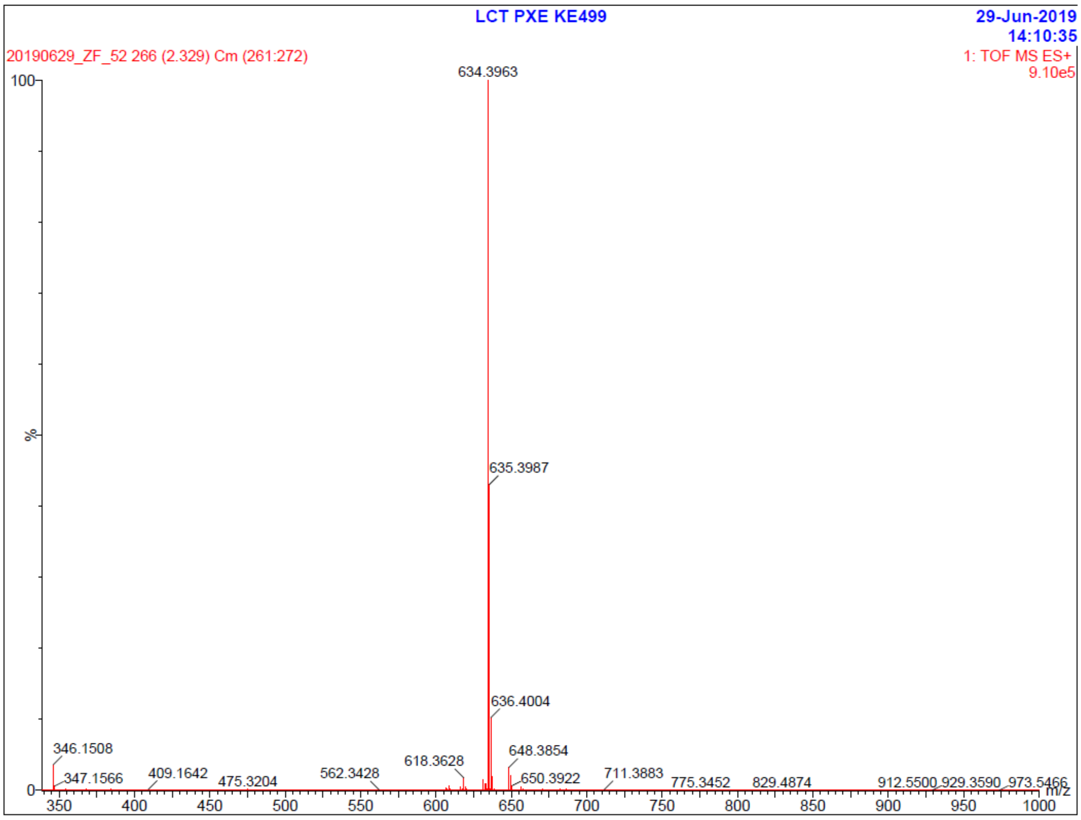

Figure SI 14-3. HR Mass spectrum (ESI) of compound **61.**

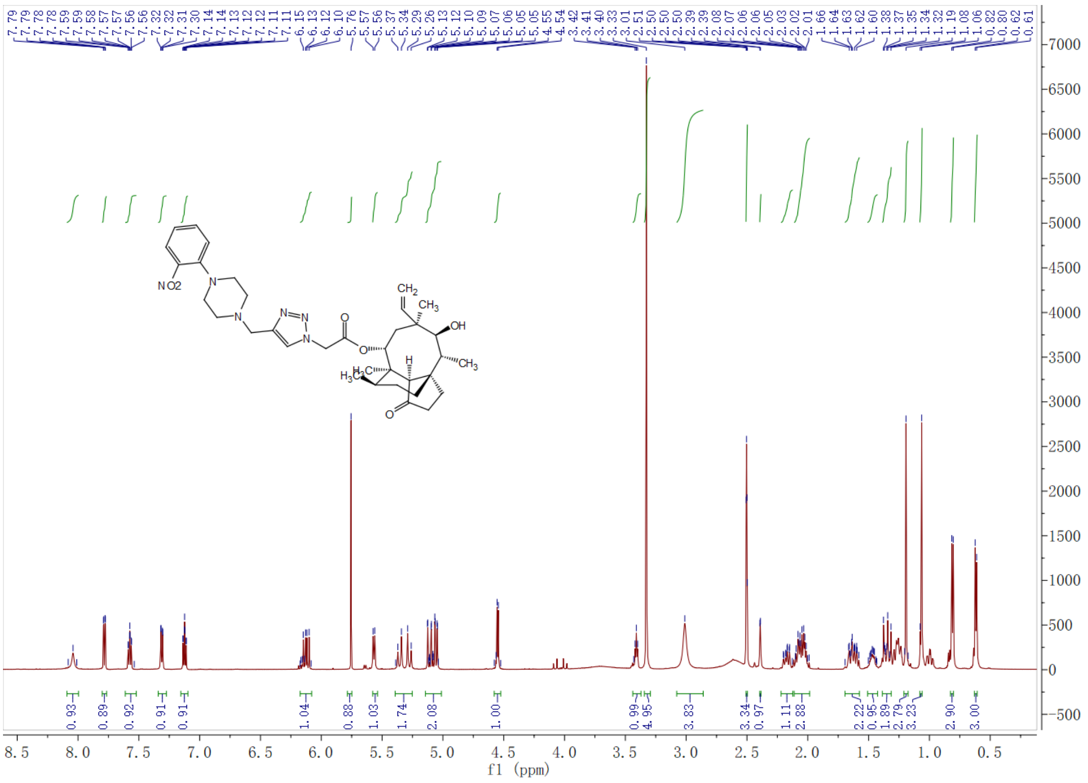

Figure SI 15-1. ^{1}H-NMR spectrum (DMSO-*d6*, 600 MHz) of compound **62.**

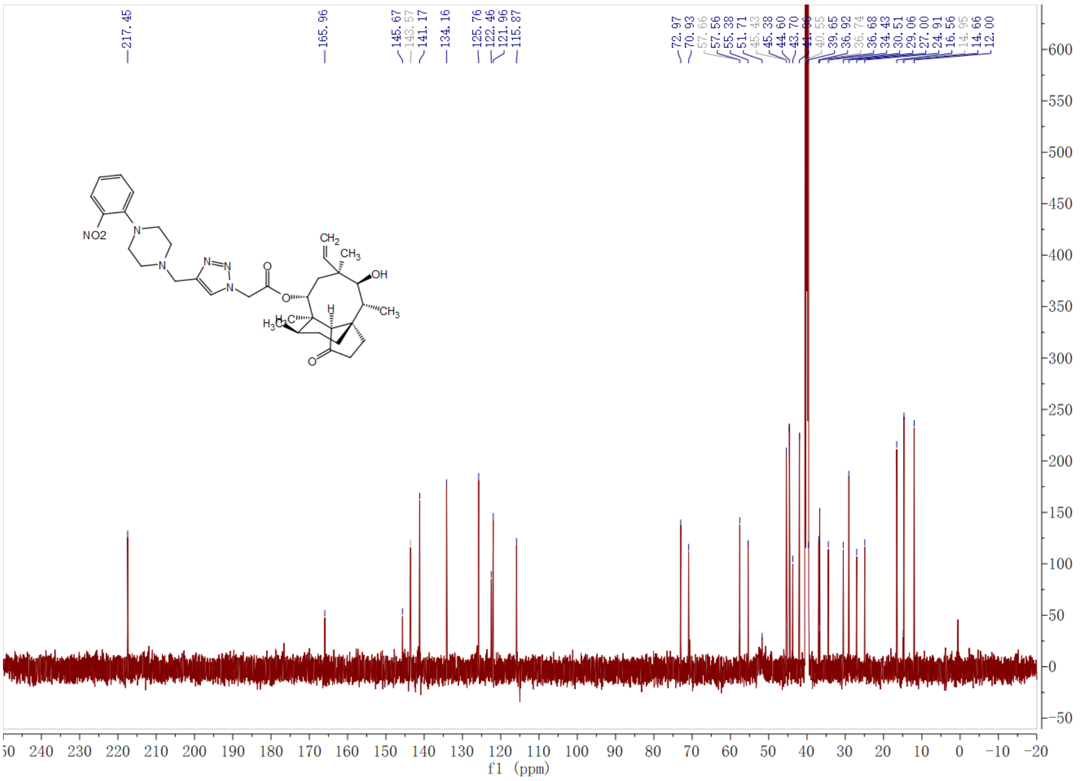

Figure SI 15-2. ^{13}C-NMR spectrum (DMSO-*d6*, 151 MHz) of compound **62.**

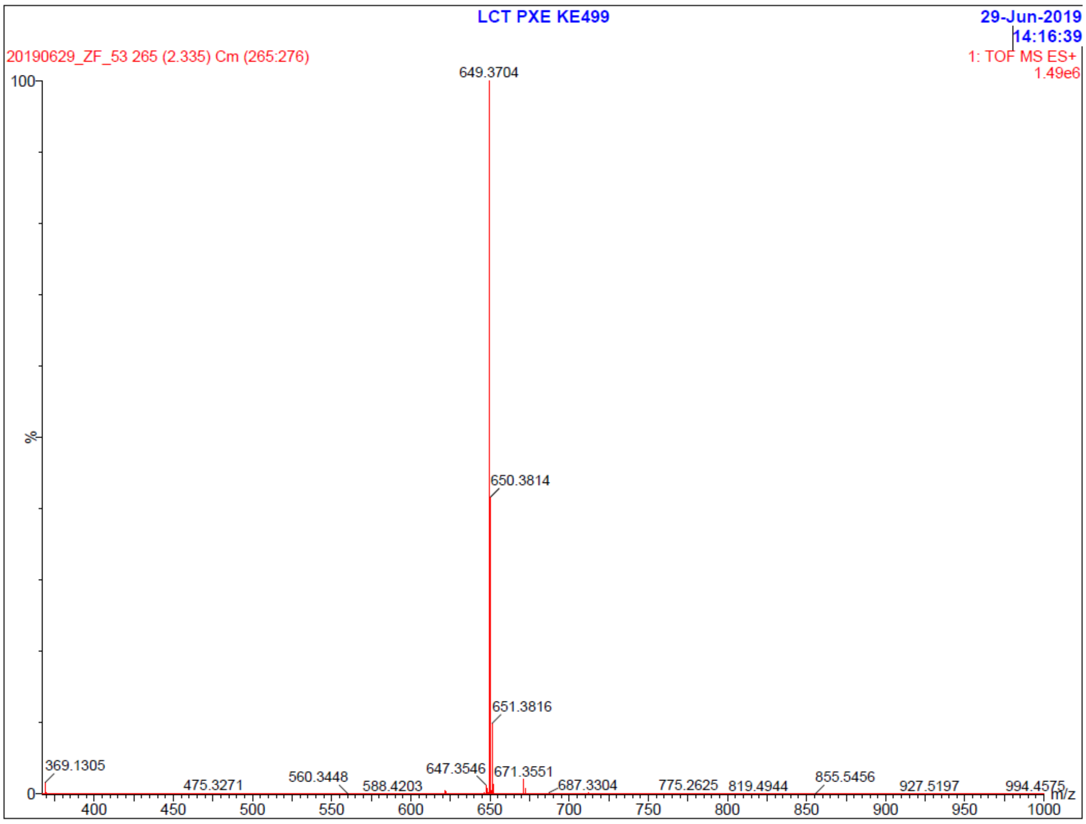

Figure SI 15-3. HR Mass spectrum (ESI) of compound **62.**

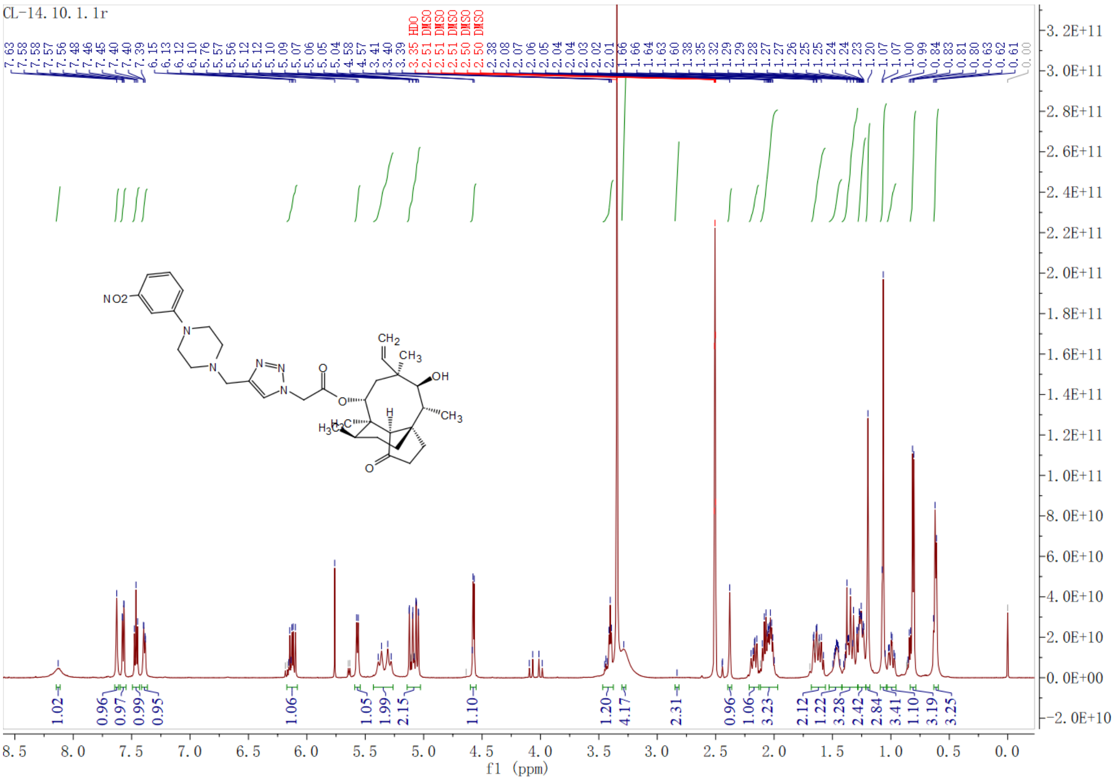

Figure SI 16-1. ^{1}H-NMR spectrum (DMSO-*d6*, 600 MHz) of compound **63.**

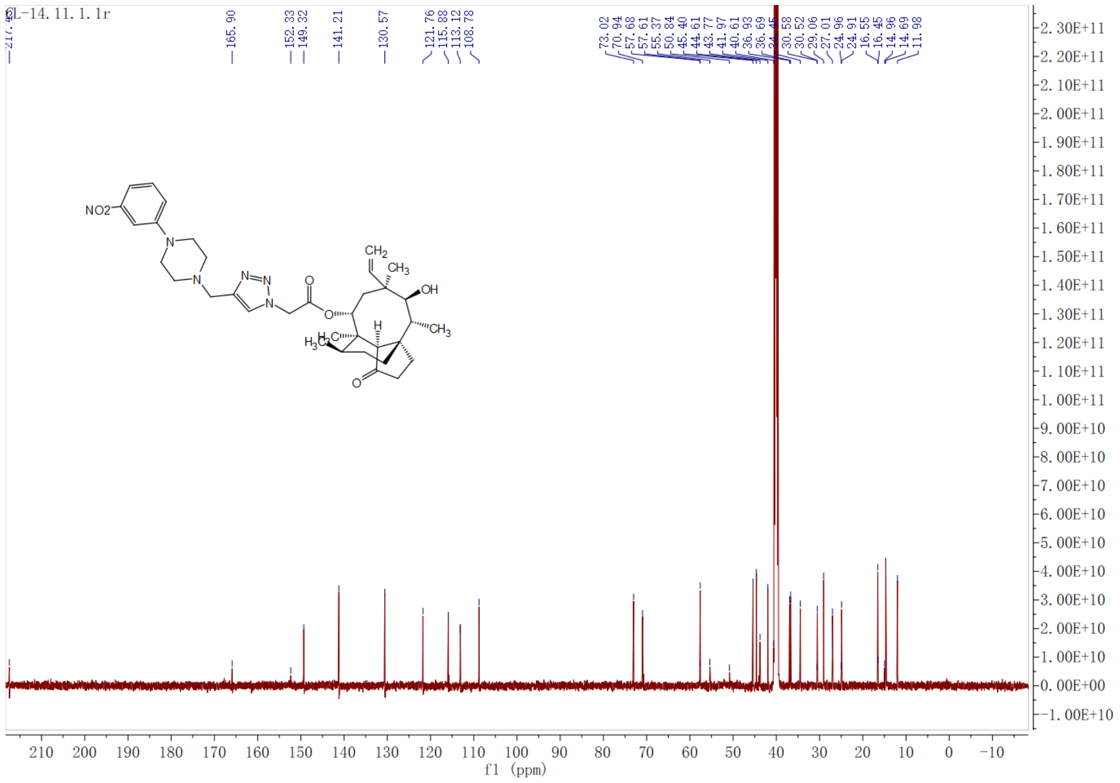

Figure SI 16-2. ^{13}C-NMR spectrum (DMSO-*d6*, 151 MHz) of compound **63.**

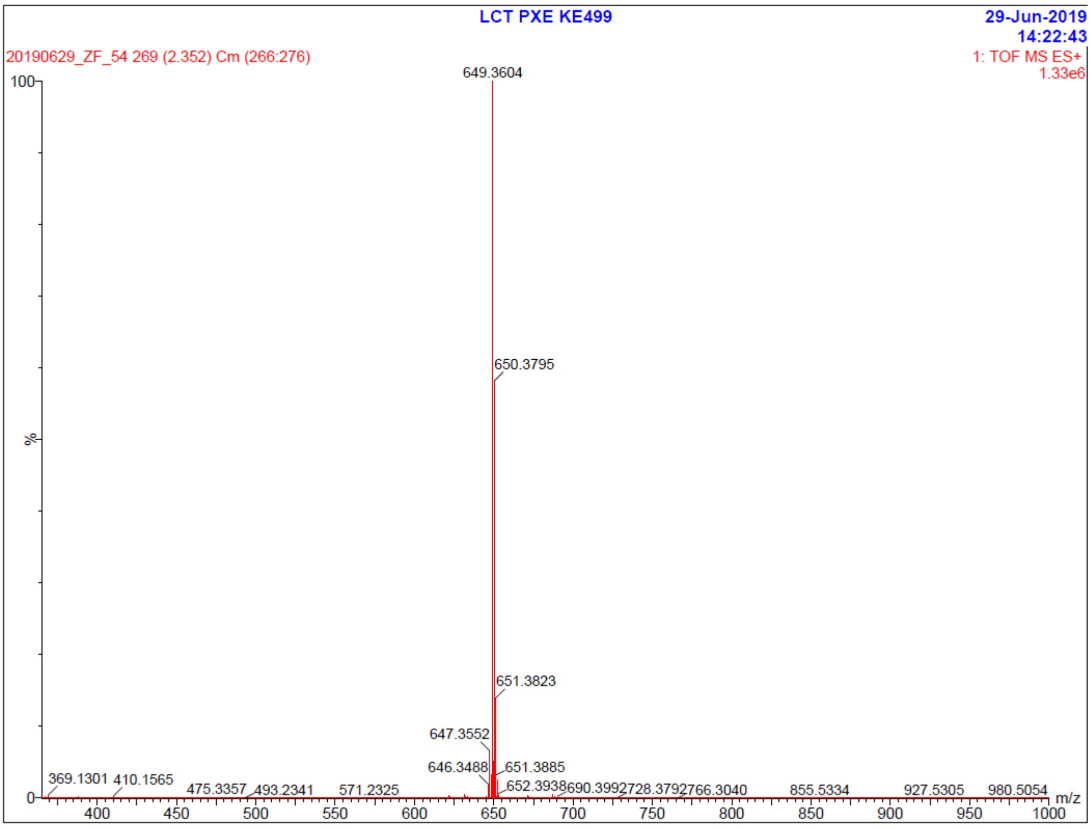

Figure SI 16-3. HR Mass spectrum (ESI) of compound **63.**

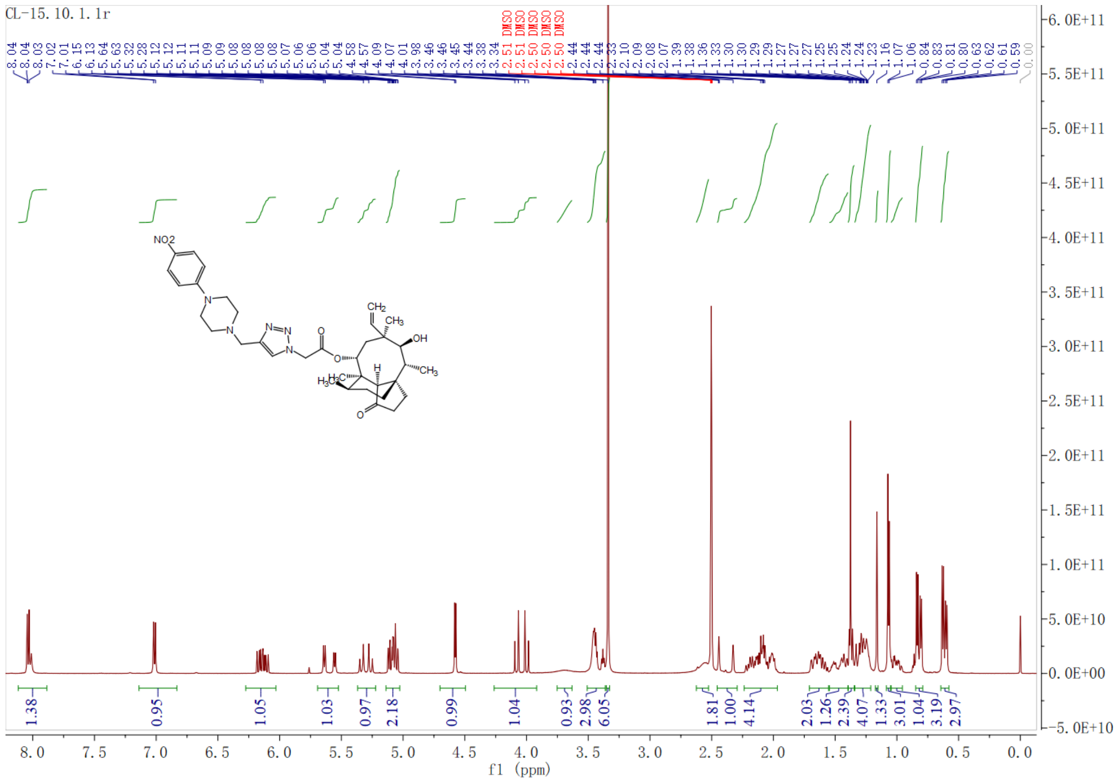

Figure SI 17-1. ^{1}H-NMR spectrum (DMSO-*d6*, 600 MHz) of compound **64.**

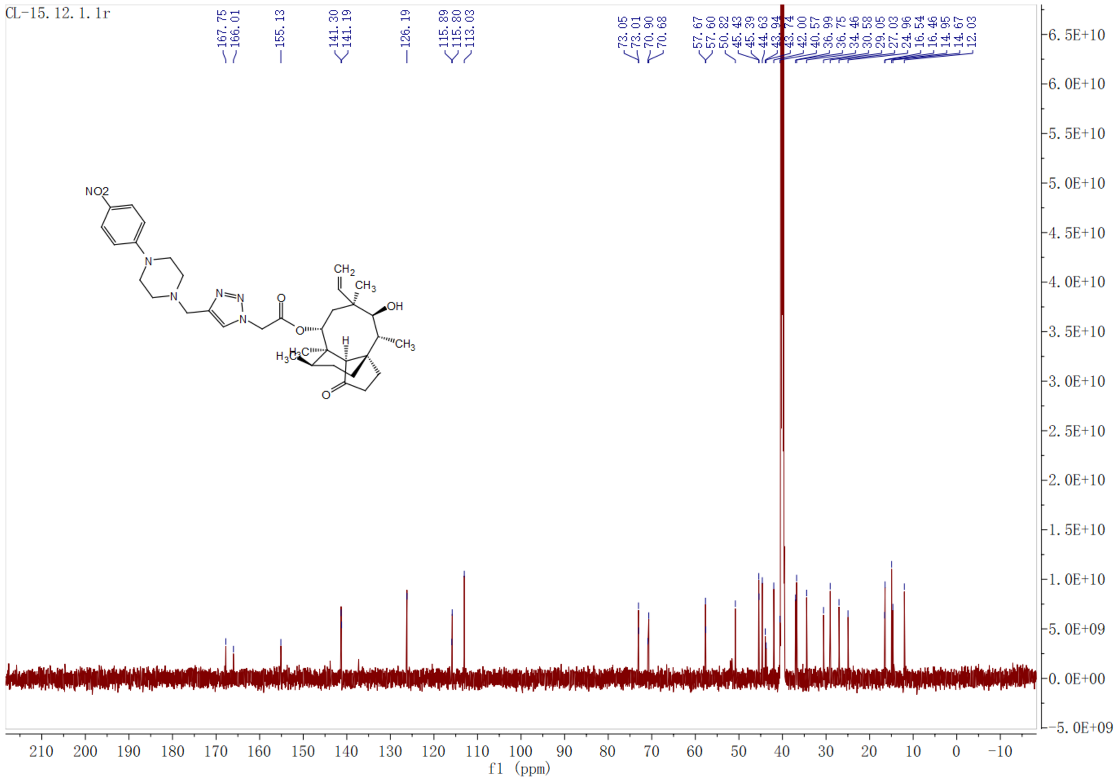

Figure SI 17-2. ^{13}C-NMR spectrum (DMSO-*d6*, 151 MHz) of compound **64.**

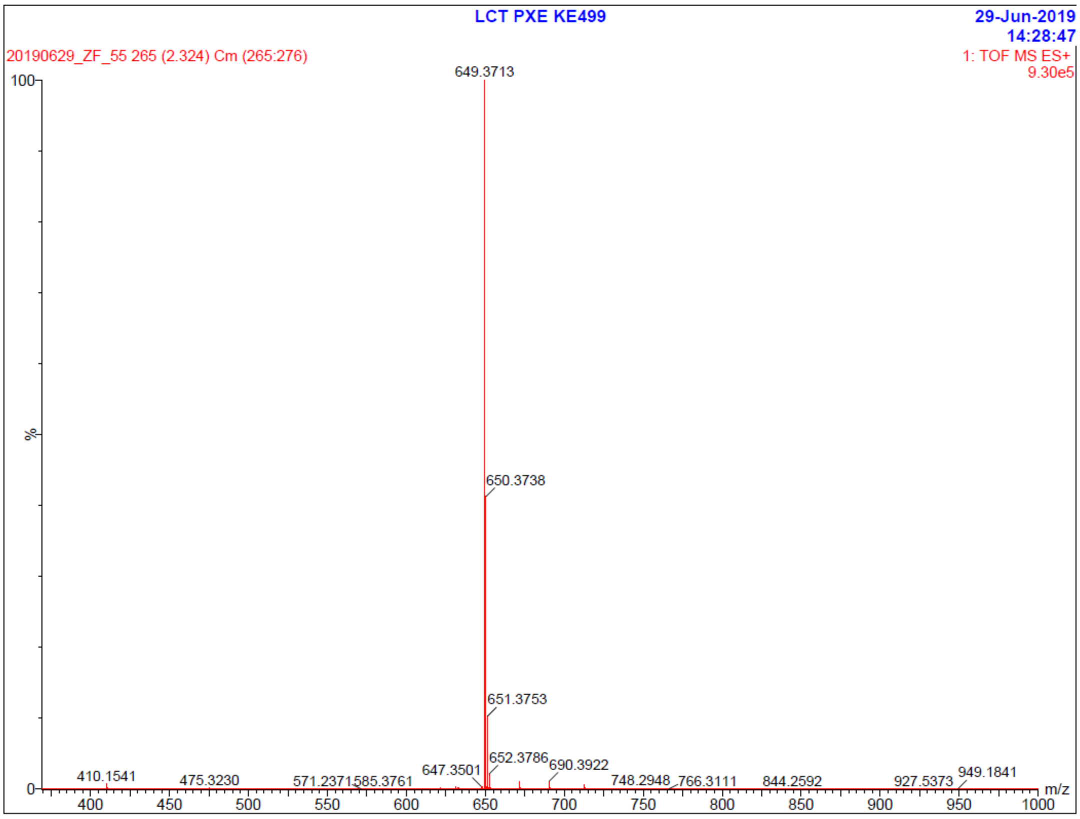

Figure SI 17-3. HR Mass spectrum (ESI) of compound **64.**

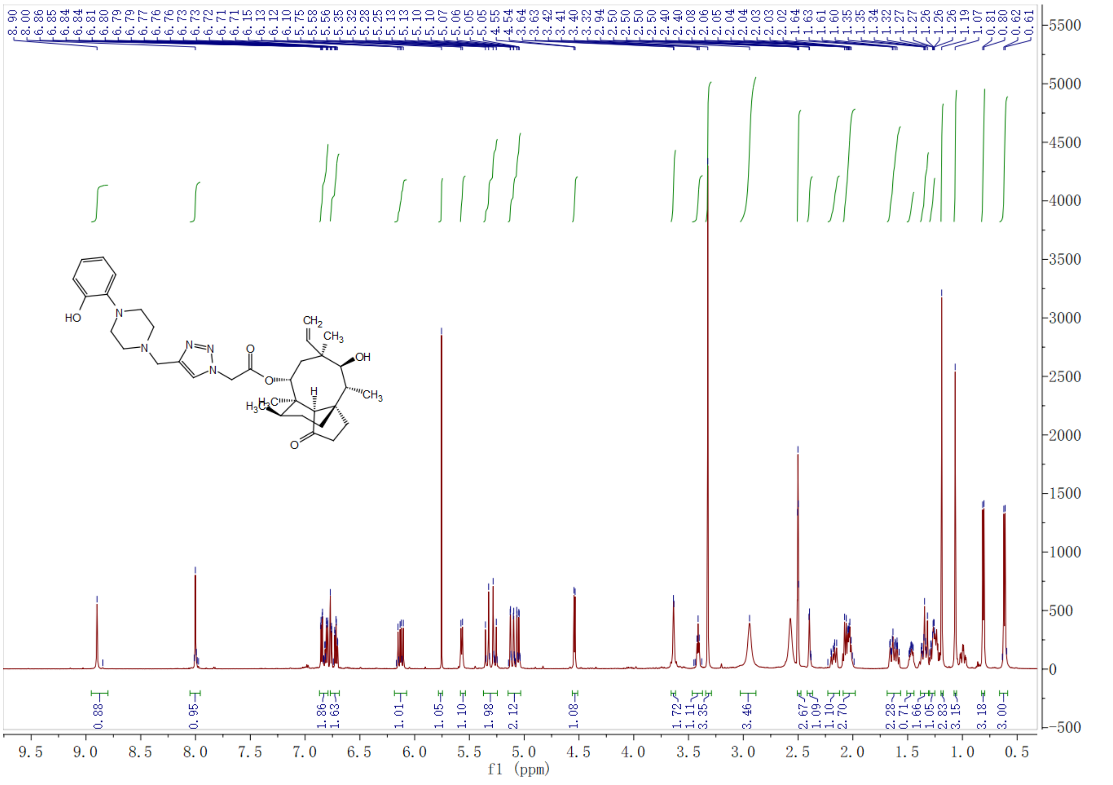

Figure SI 18-1. ^{1}H-NMR spectrum (DMSO-*d6*, 600 MHz) of compound **65.**

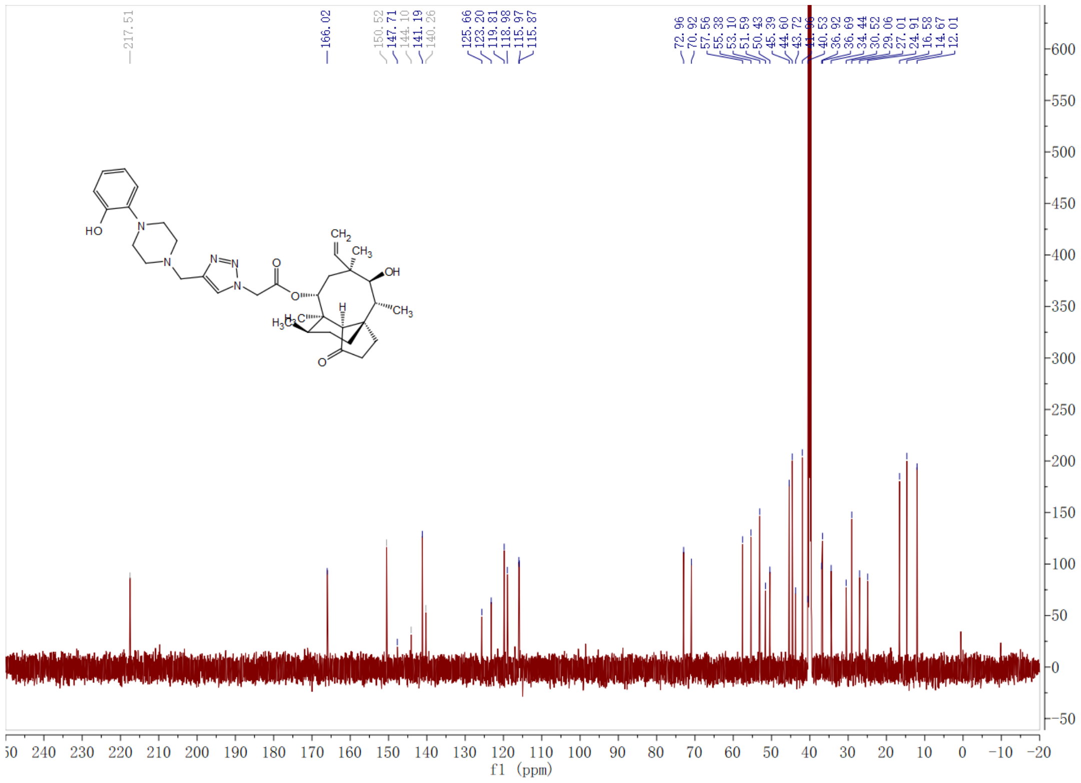

Figure SI 18-2. ^{13}C-NMR spectrum (DMSO-*d6*, 151 MHz) of compound **65.**

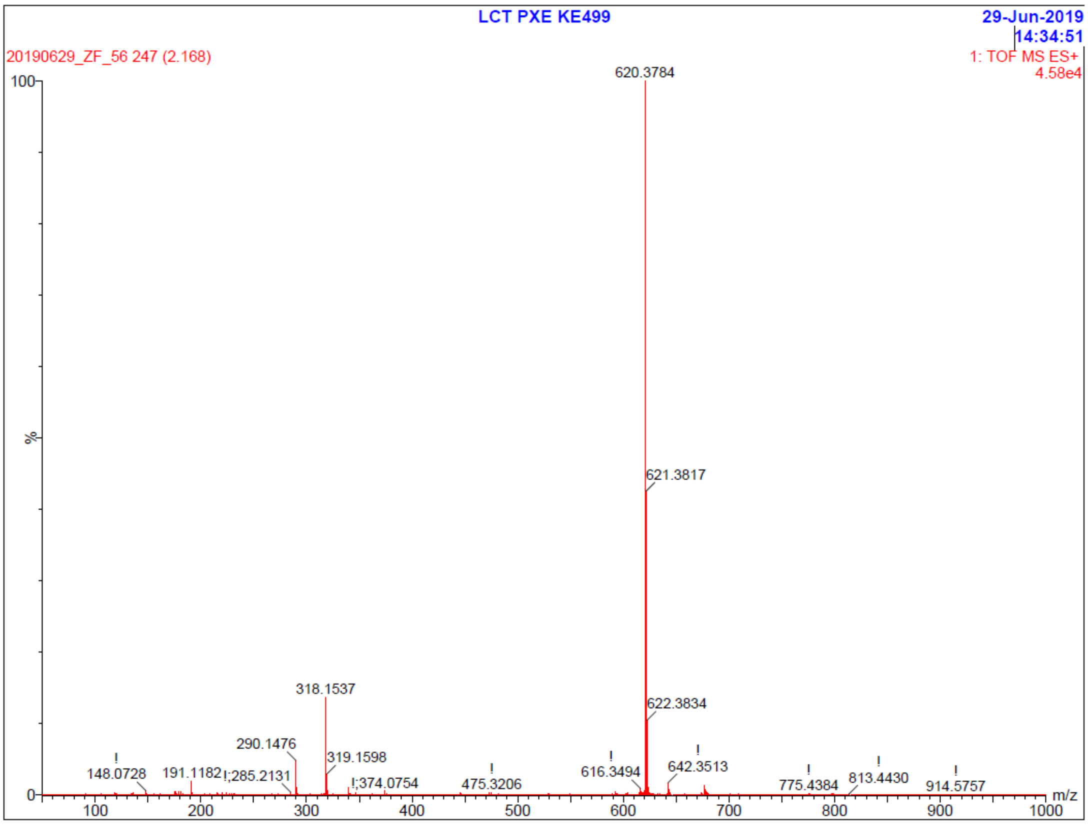

Figure SI 18-3. HR Mass spectrum (ESI) of compound **65.**

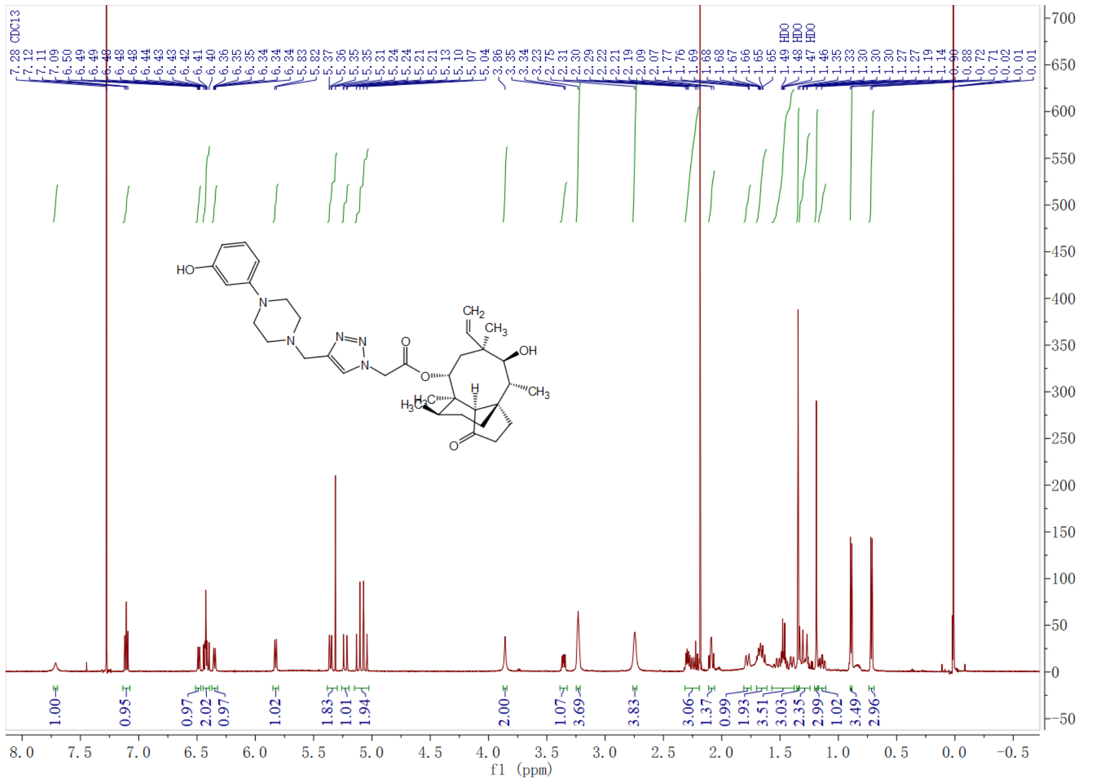

Figure SI 19-1. ^{1}H-NMR spectrum (Chloroform-*d*, 600 MHz) of compound **66.**

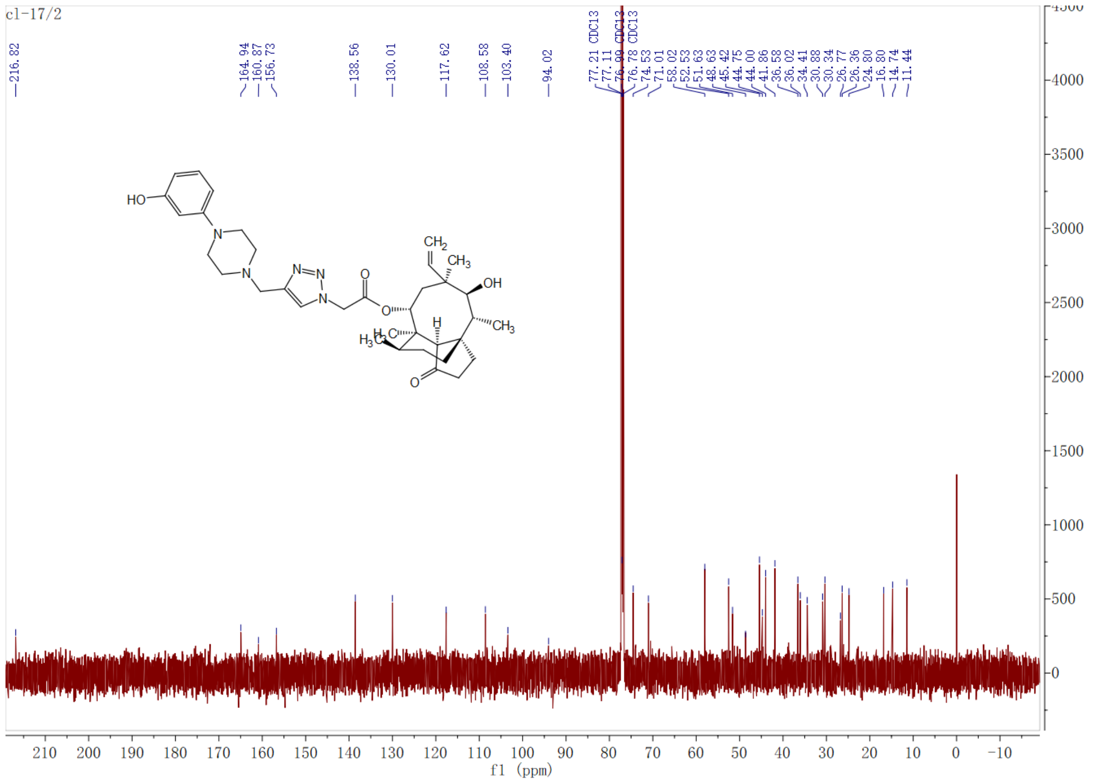

Figure SI 19-2. ^{13}C-NMR spectrum (Chloroform-*d*, 151 MHz) of compound **66.**

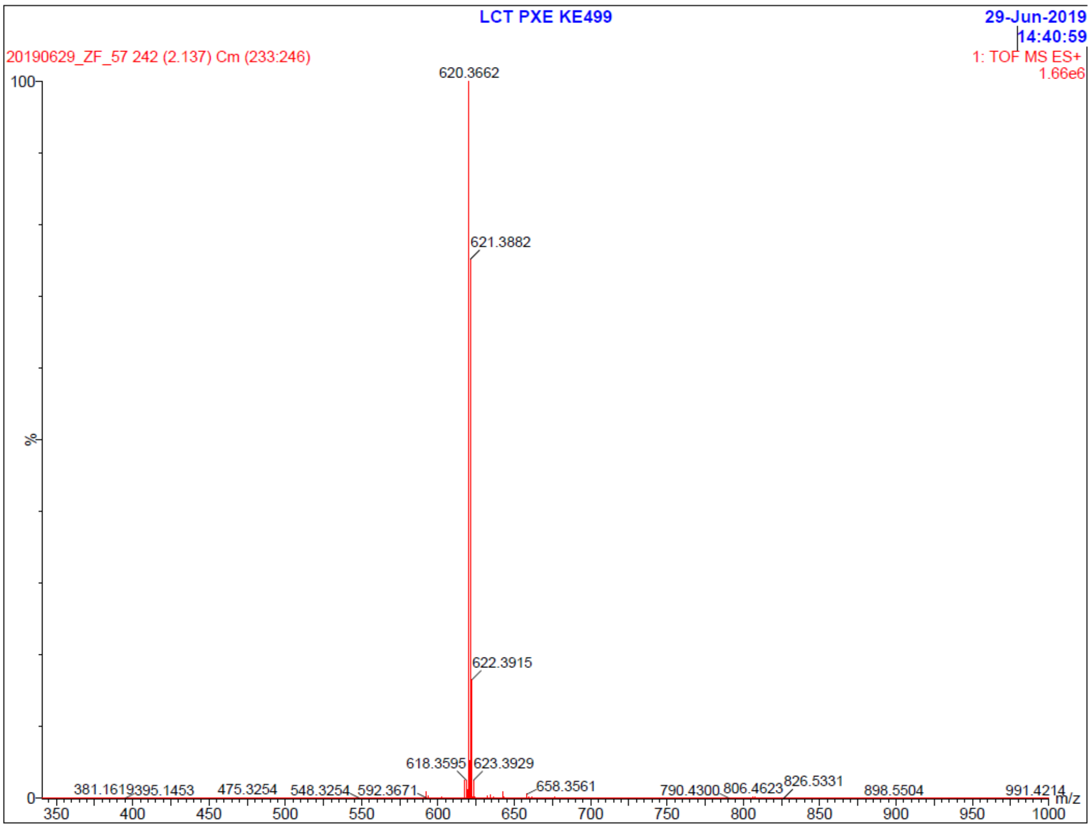

Figure SI 19-3. HR Mass spectrum (ESI) of compound **66.**

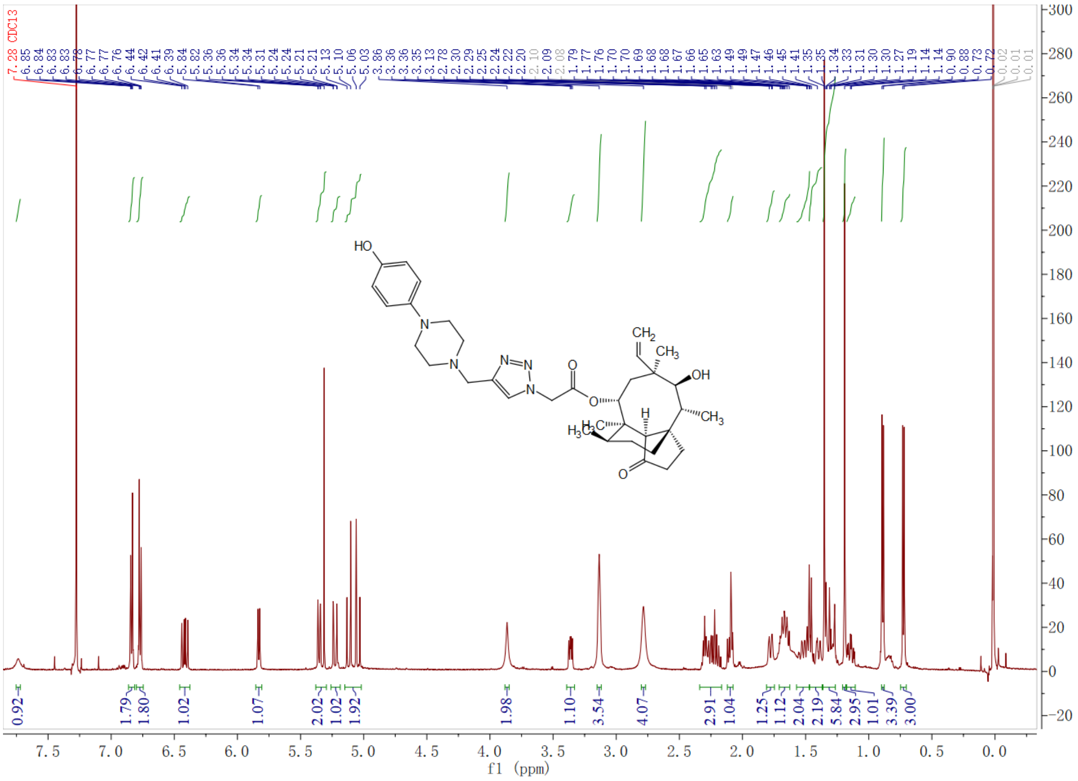

Figure SI 20-1. ^{1}H-NMR spectrum (Chloroform-*d*, 600 MHz) of compound **67.**

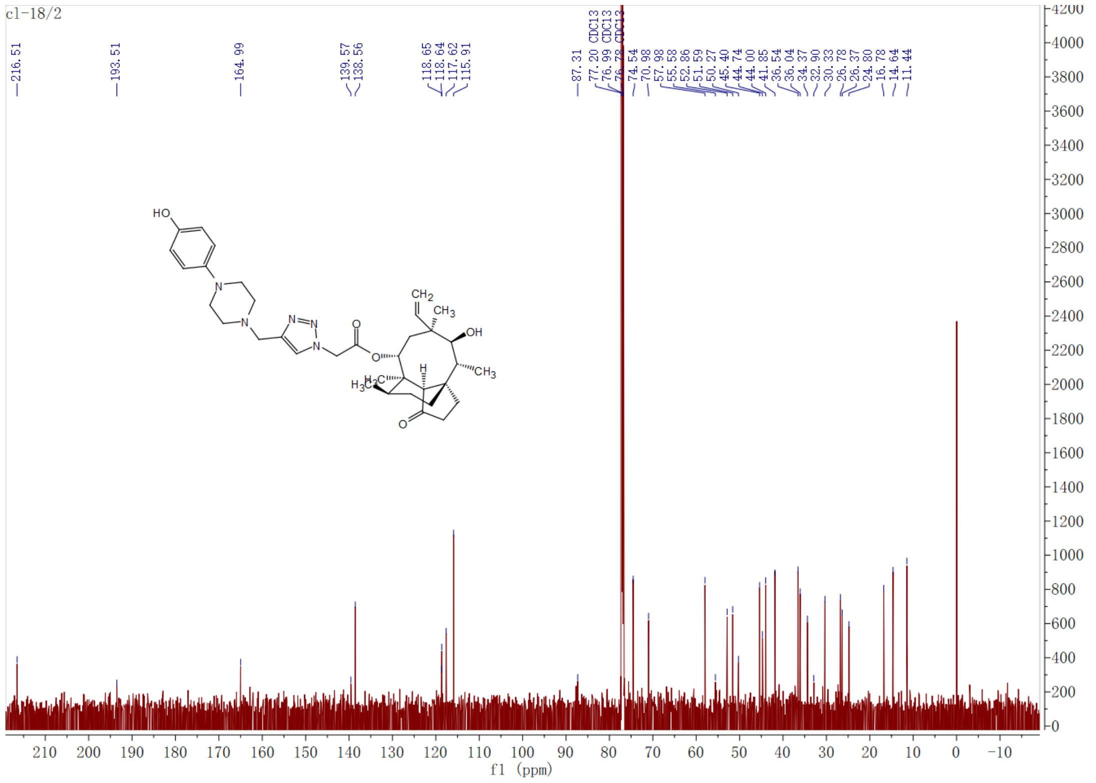

Figure SI 20-2. ^{13}C-NMR spectrum (Chloroform-*d*, 151 MHz) of compound **67.**

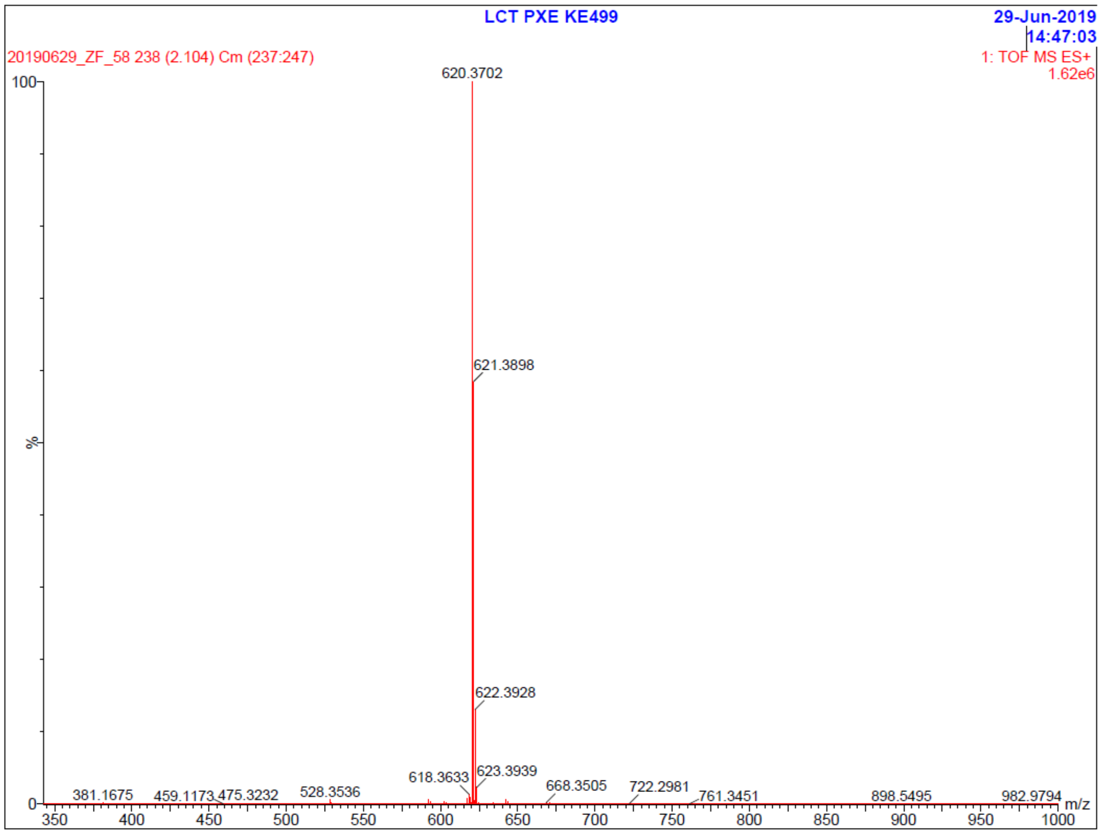

Figure SI 20-3. HR Mass spectrum (ESI) of compound **67.**

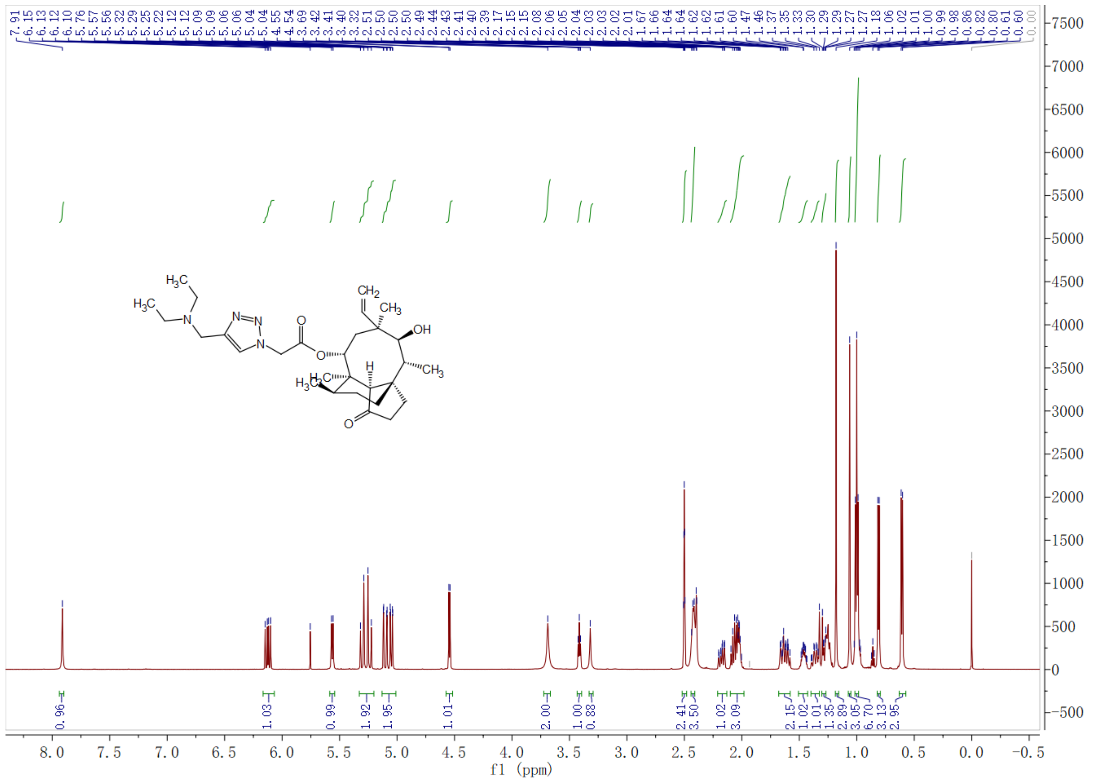

Figure SI 21-1. ^{1}H-NMR spectrum (DMSO-*d6*, 600 MHz) of compound **79.**

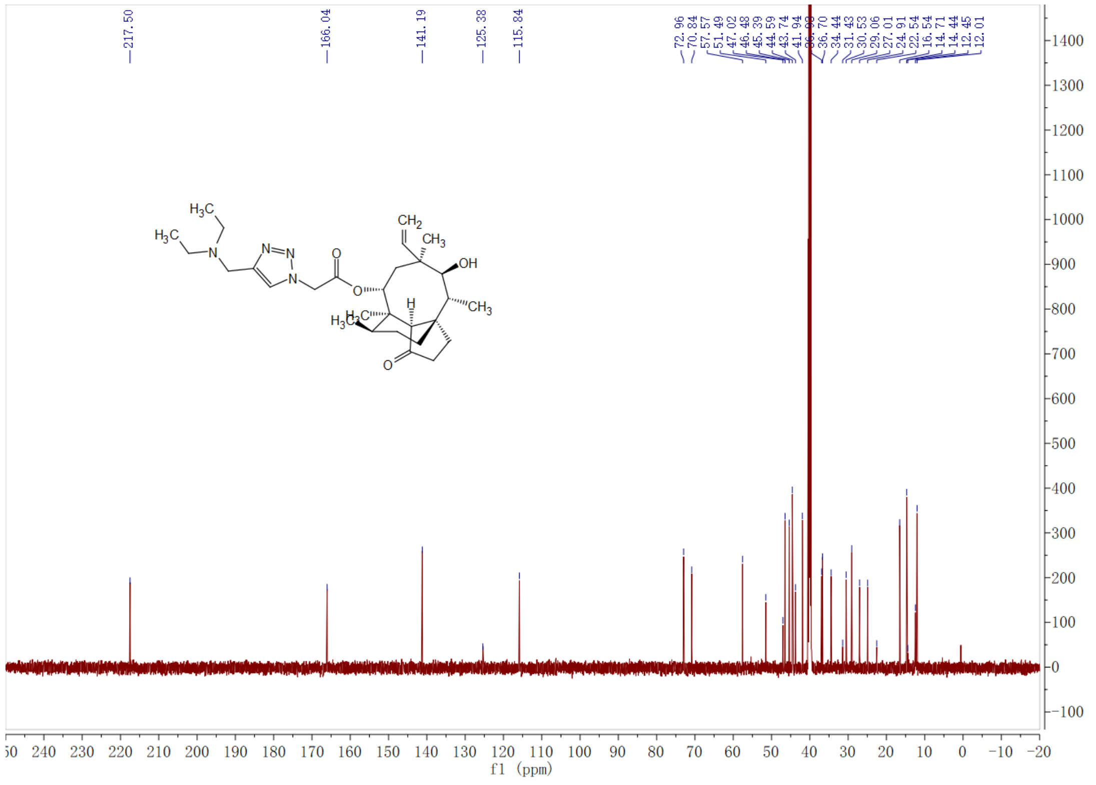

Figure SI 21-2. ^{13}C-NMR spectrum (DMSO-*d6*, 151 MHz) of compound **79.**

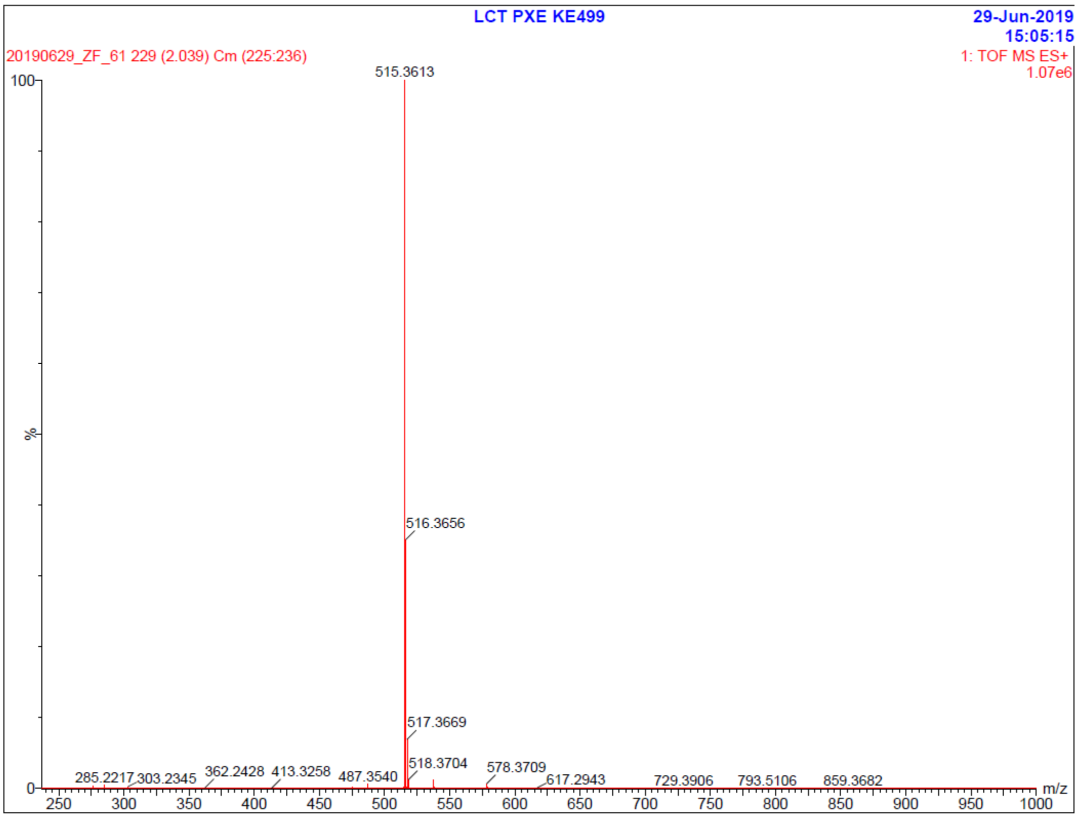

Figure SI 21-3. HR Mass spectrum (ESI) of compound **79.**

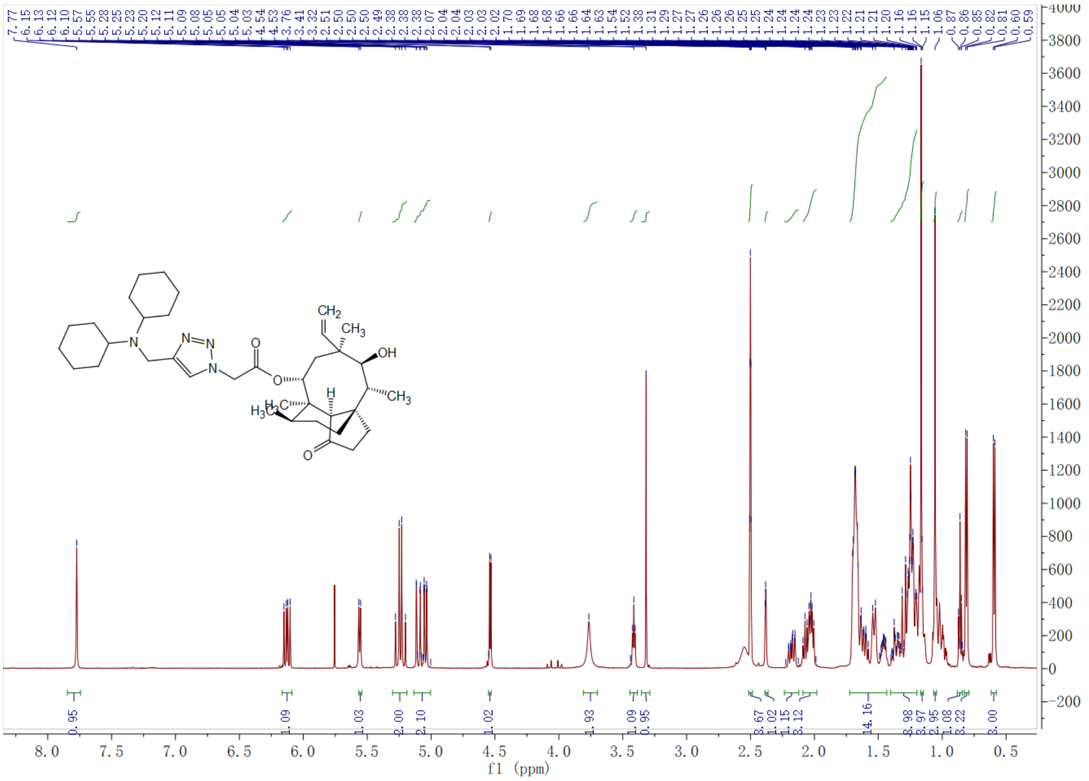

Figure SI 22-1. ^{1}H-NMR spectrum (DMSO-*d6*, 600 MHz) of compound **80.**

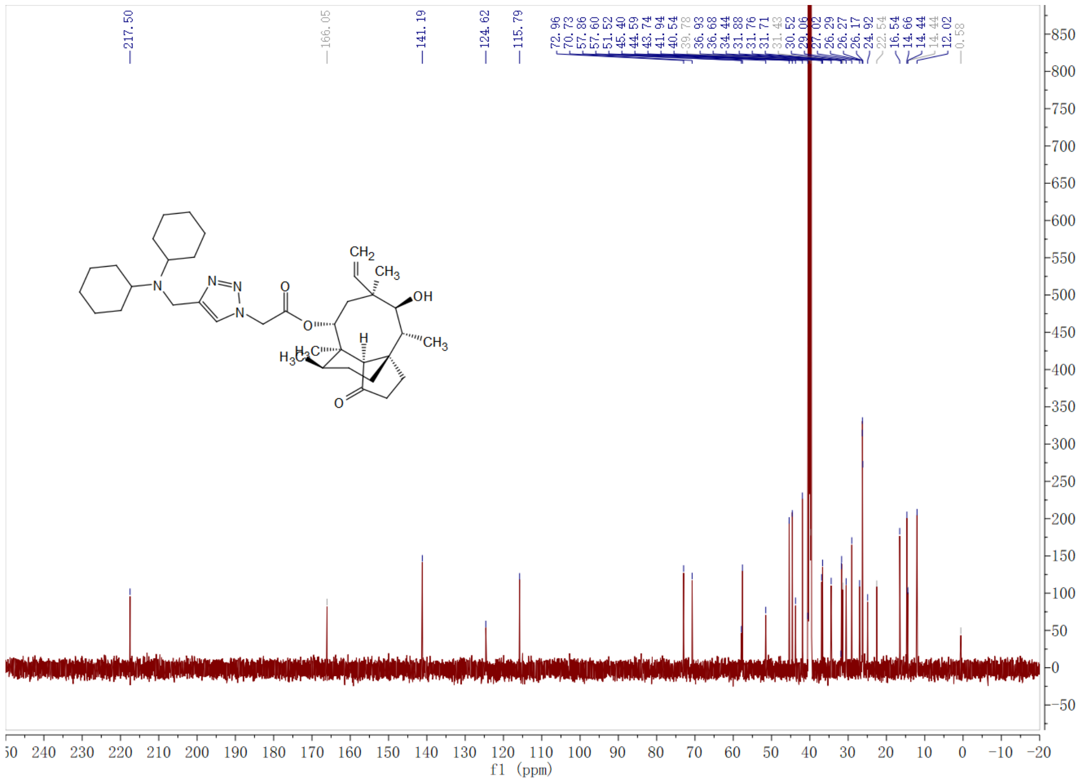

Figure SI 22-2. ^{13}C-NMR spectrum (DMSO-*d6*, 151 MHz) of compound **80.**

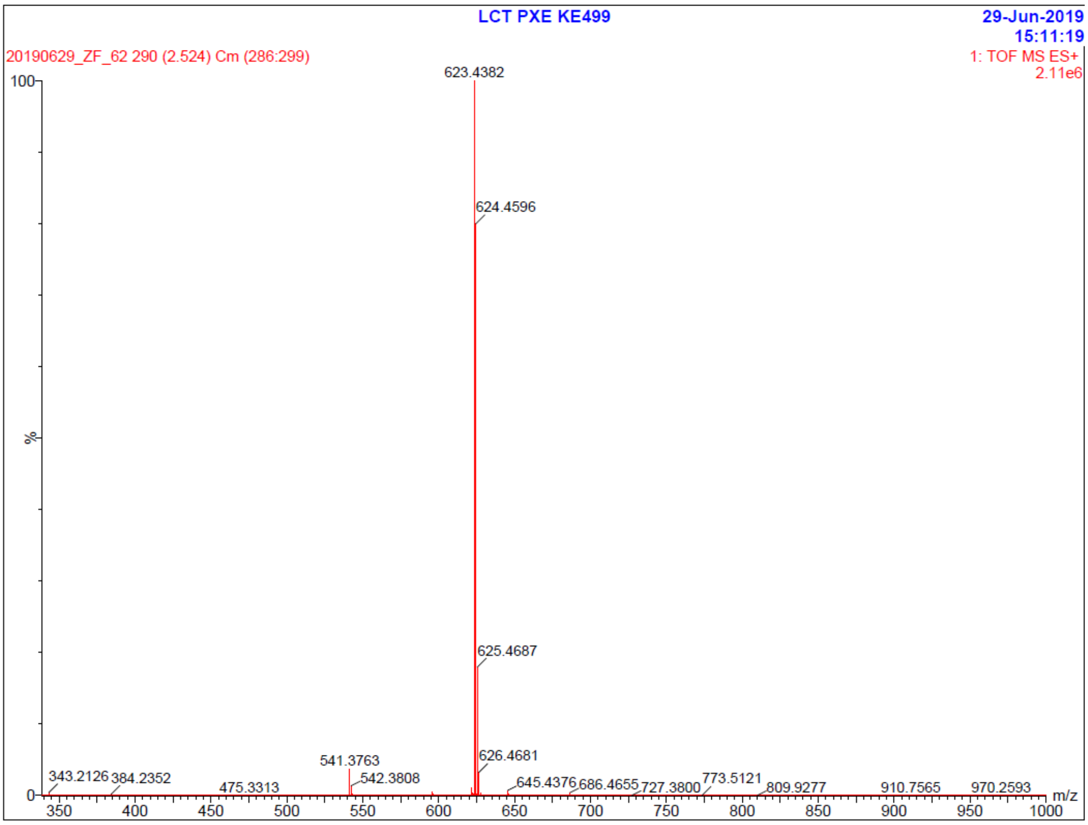

Figure SI 22-3. HR Mass spectrum (ESI) of compound **80.**

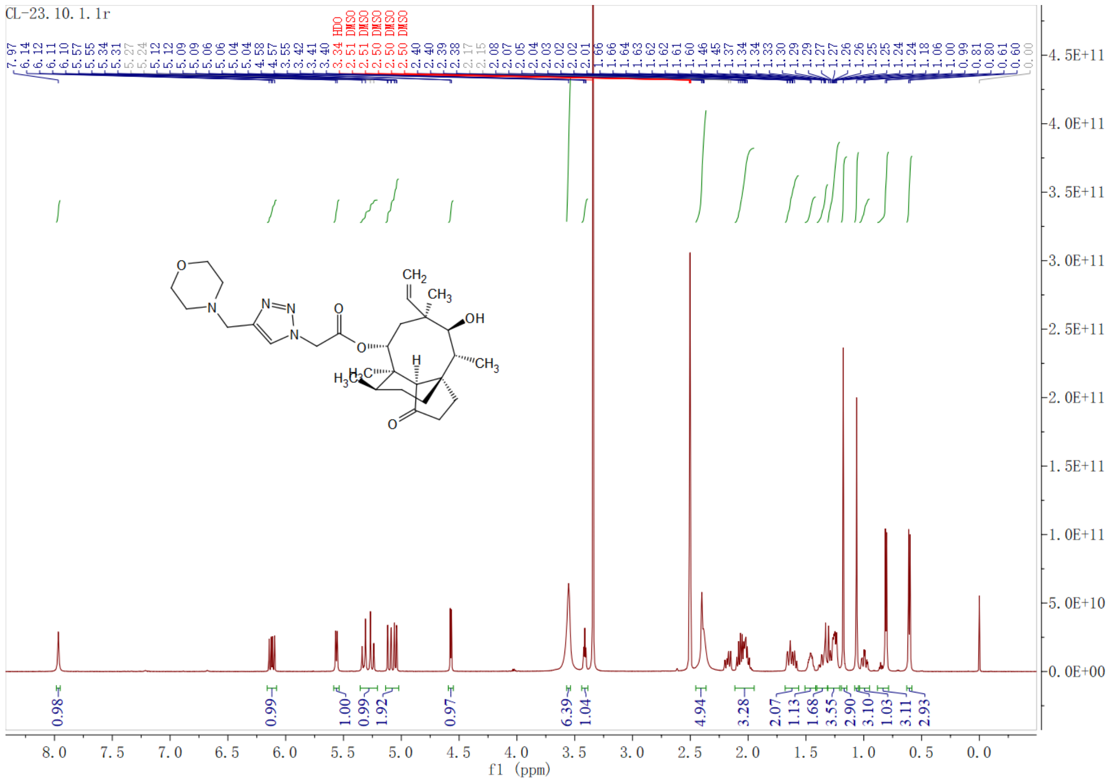

Figure SI 23-1. ^{1}H-NMR spectrum (DMSO-*d6*, 600 MHz) of compound **81.**

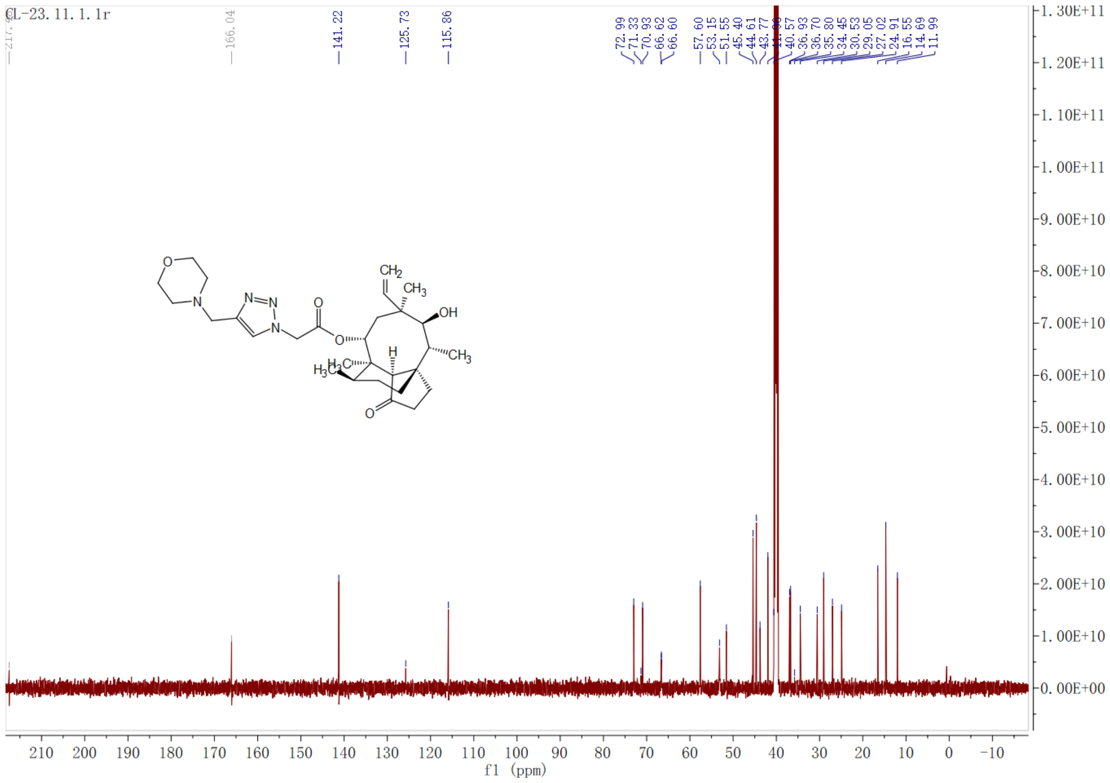

Figure SI 23-2. ^{13}C-NMR spectrum (DMSO-*d6*, 151 MHz) of compound **81.**

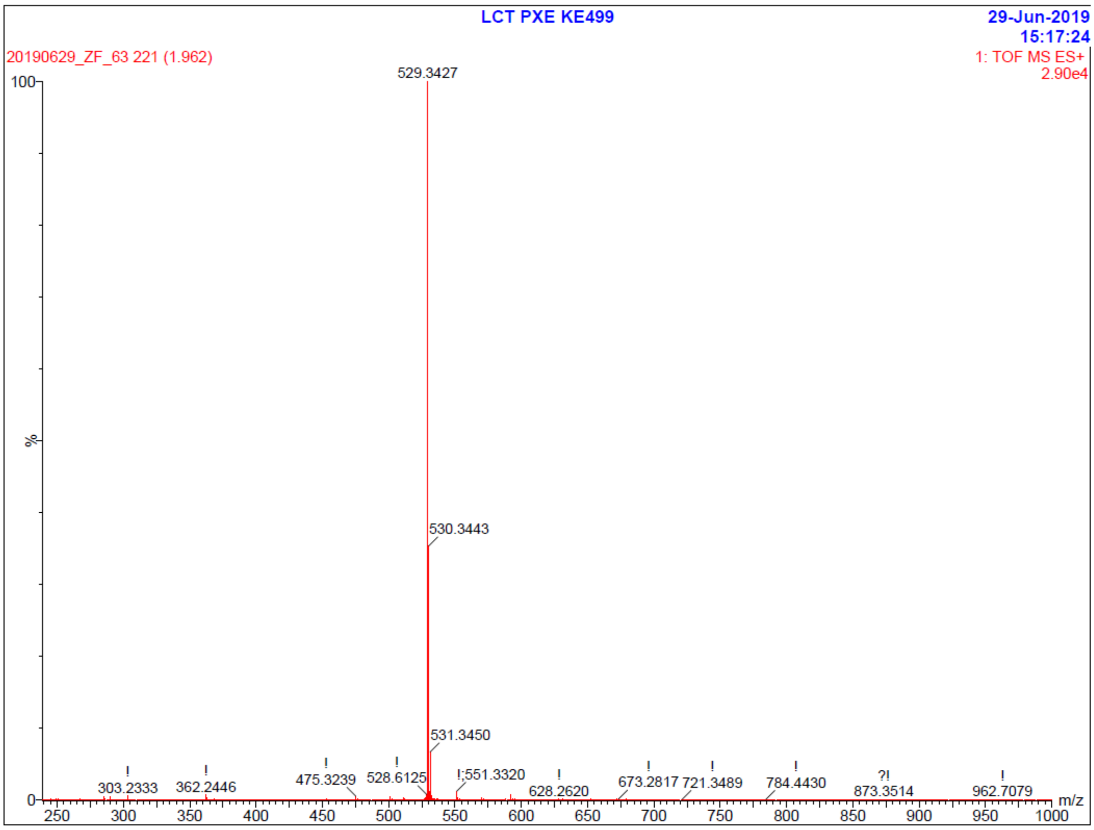

Figure SI 23-3. HR Mass spectrum (ESI) of compound **81.**

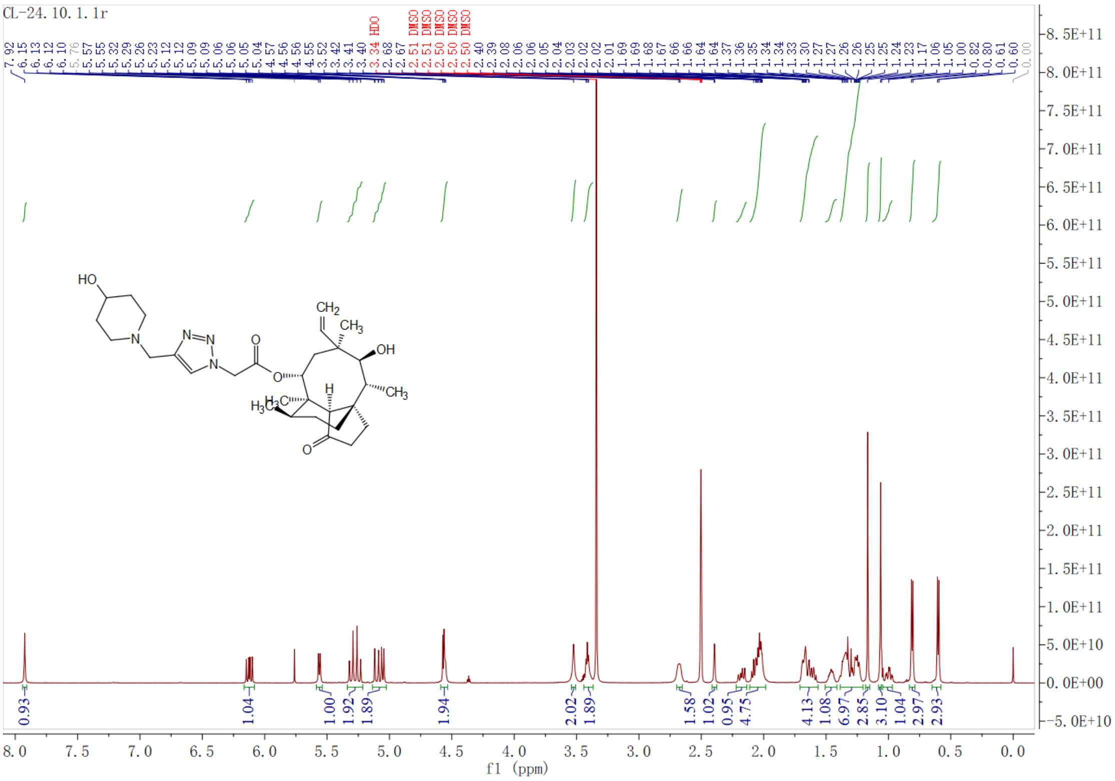

Figure SI 24-1. ¹H-NMR spectrum (DMSO-*d6*, 600 MHz) of compound **82.**

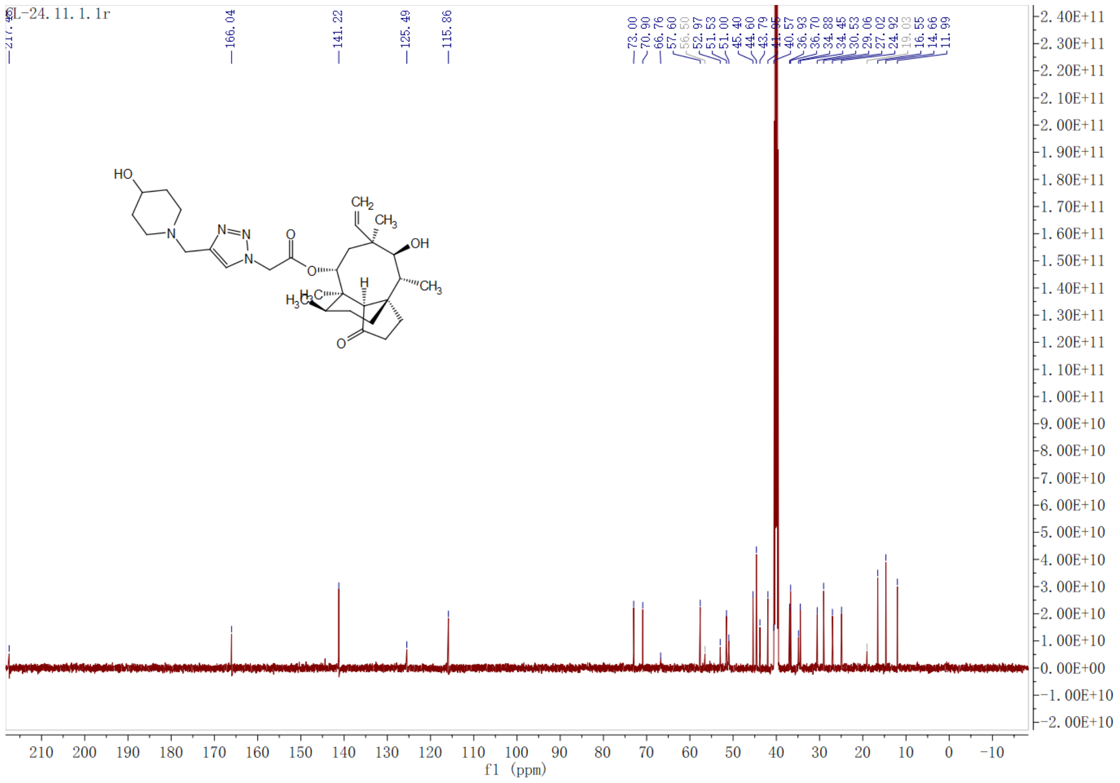

Figure SI 24-2. ^{13}C-NMR spectrum (DMSO-*d6*, 151 MHz) of compound **82.**

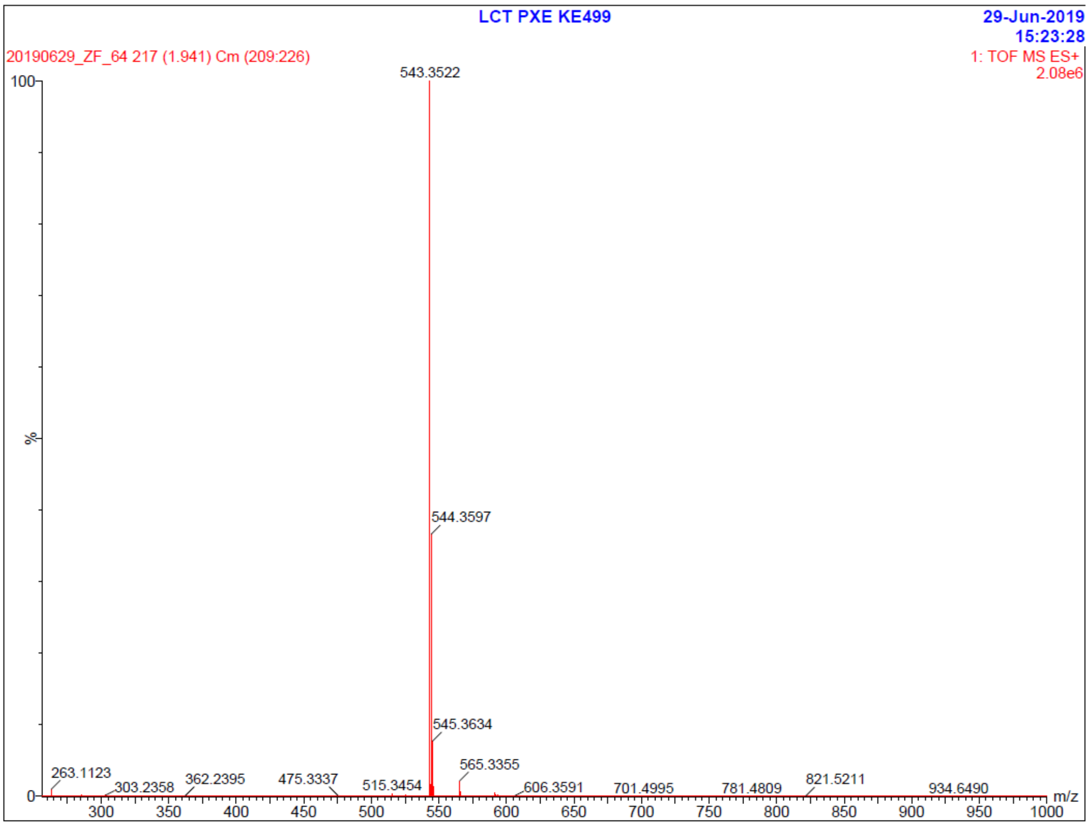

Figure SI 24-3. HR Mass spectrum (ESI) of compound **82.**

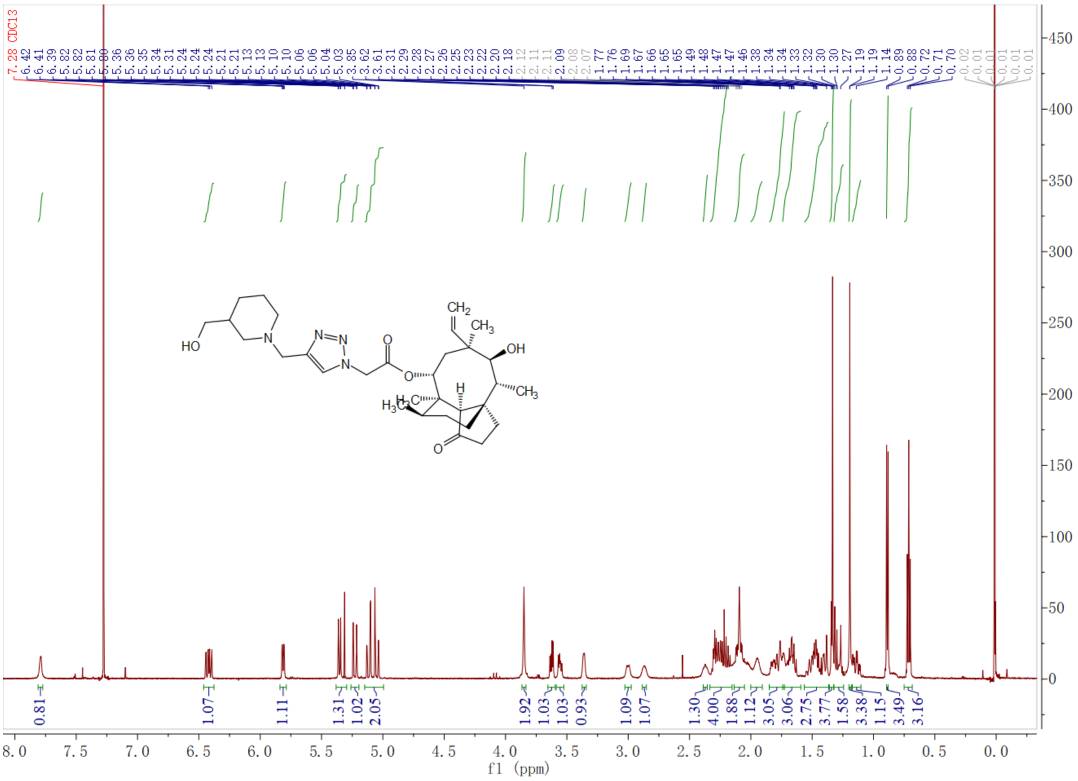

Figure SI 25-1. ^{1}H-NMR spectrum (Chloroform-*d*, 600 MHz) of compound **83.**

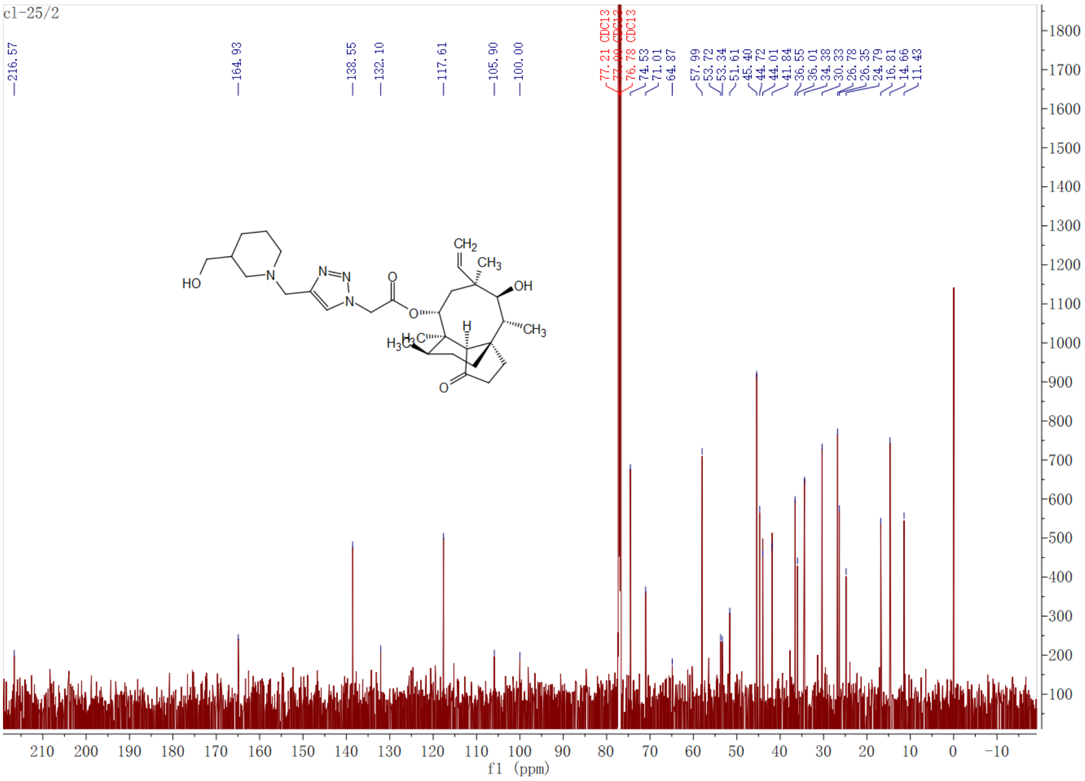

Figure SI 25-2. ^{13}C-NMR spectrum (Chloroform-*d*, 151 MHz) of compound **83.**

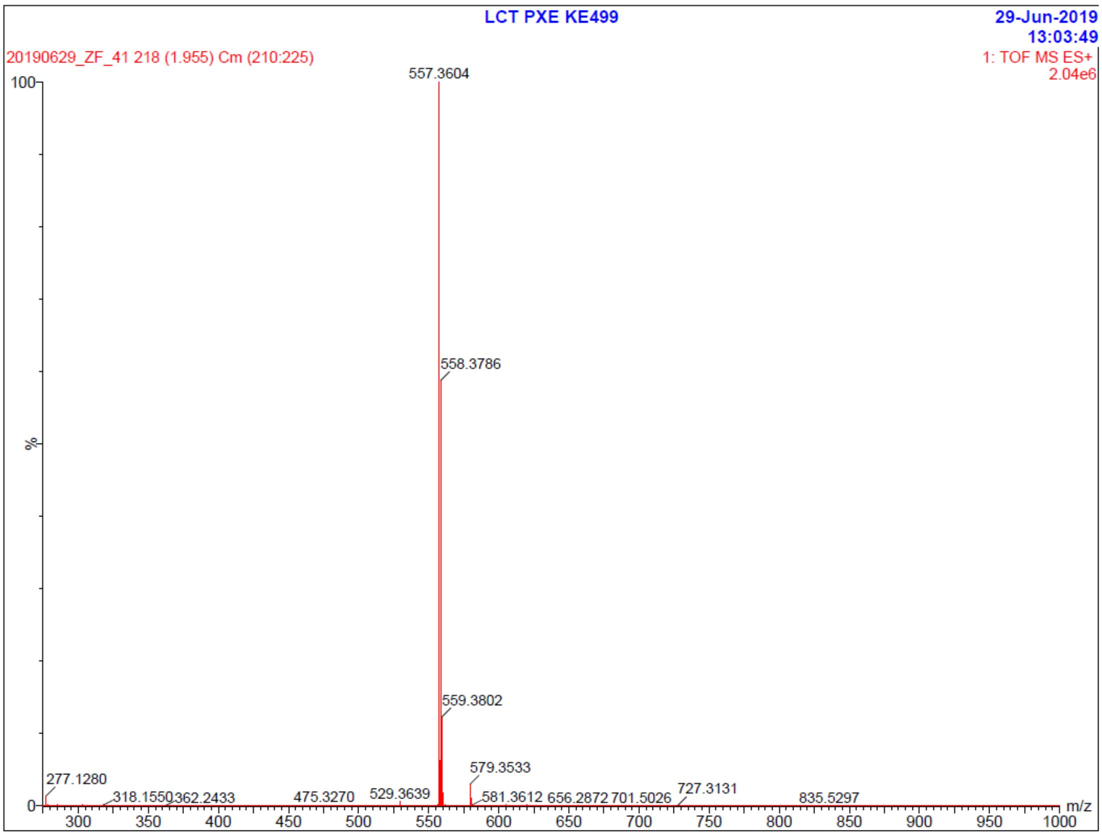

Figure SI 25-3. HR Mass spectrum (ESI) of compound **83.**

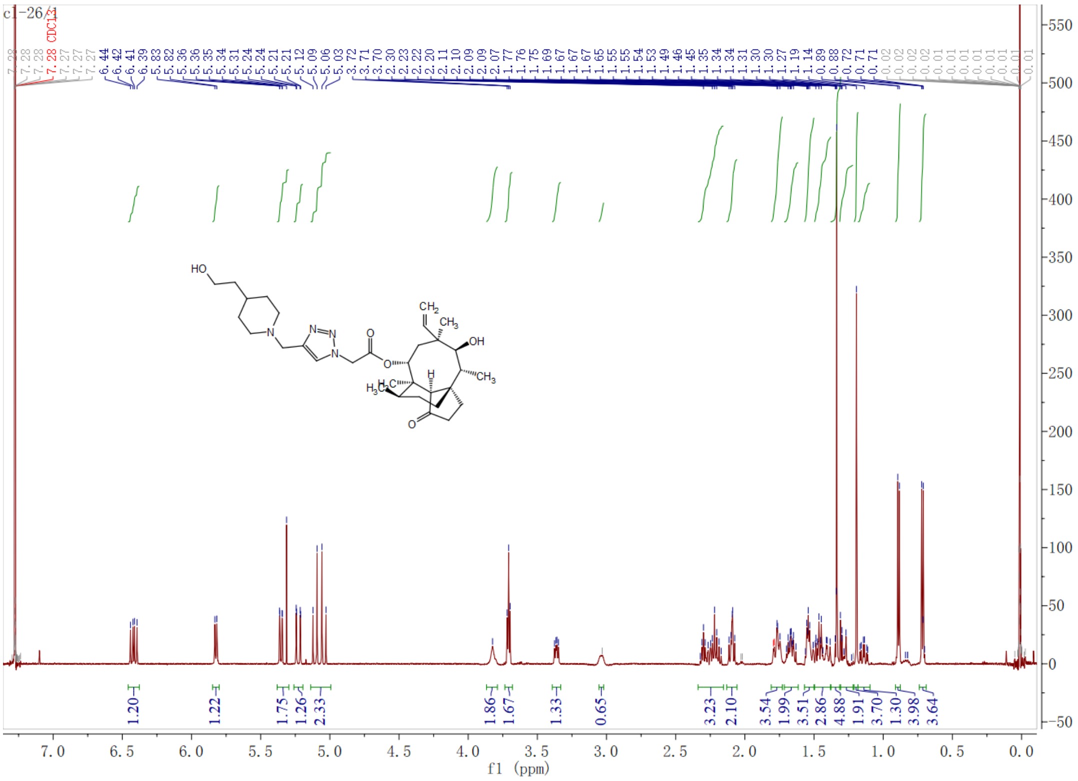

Figure SI 26-1. ^{1}H-NMR spectrum (Chloroform-d, 600 MHz) of compound **84.**

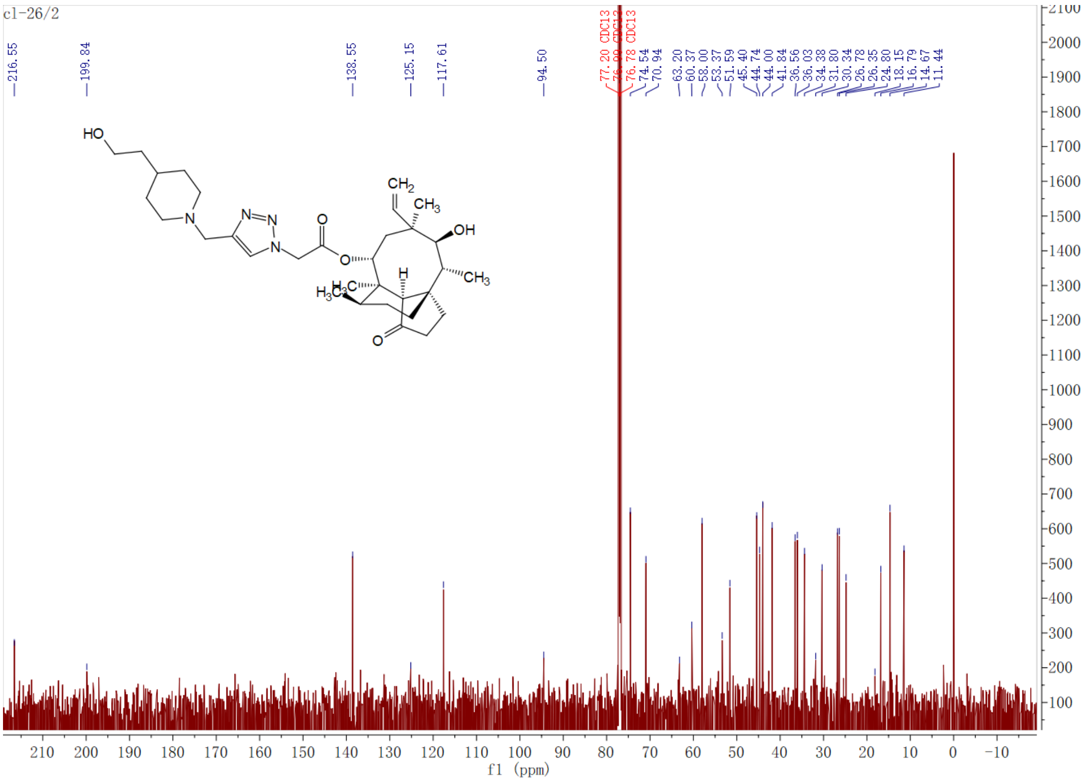

Figure SI 26-2. ^{13}C-NMR spectrum (Chloroform-*d*, 151 MHz) of compound **84.**

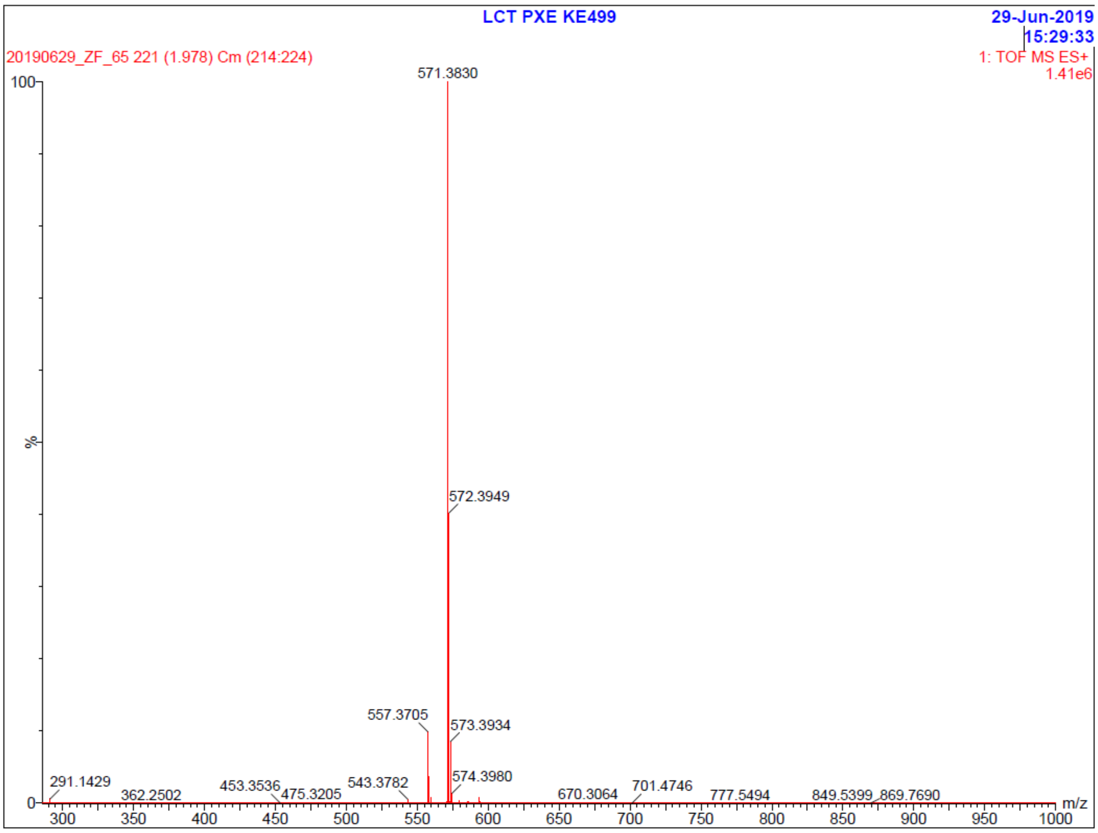

Figure SI 26-3. HR Mass spectrum (ESI) of compound **84.**

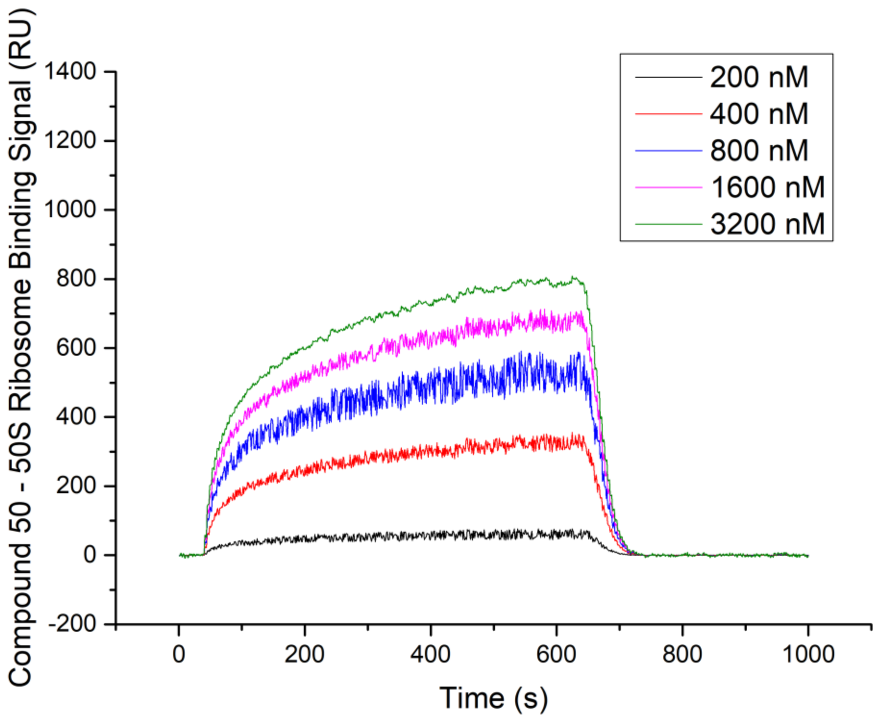

Figure SI 27. Concentration gradient curve of SPR binding signal between compound **50** and *50S* ribosome. A set of *50S* ribosome with concentration gradients (200 nM, 400 nM, 800 nM, 1600 nM, 3200 nM) were circulated in the SPR experiment; the picture shows the interaction and binding curves of different concentrations of *50S* ribosome and selected compounds. The figures after this (**Figure SI 28~34**) have the same meanings as this figure.

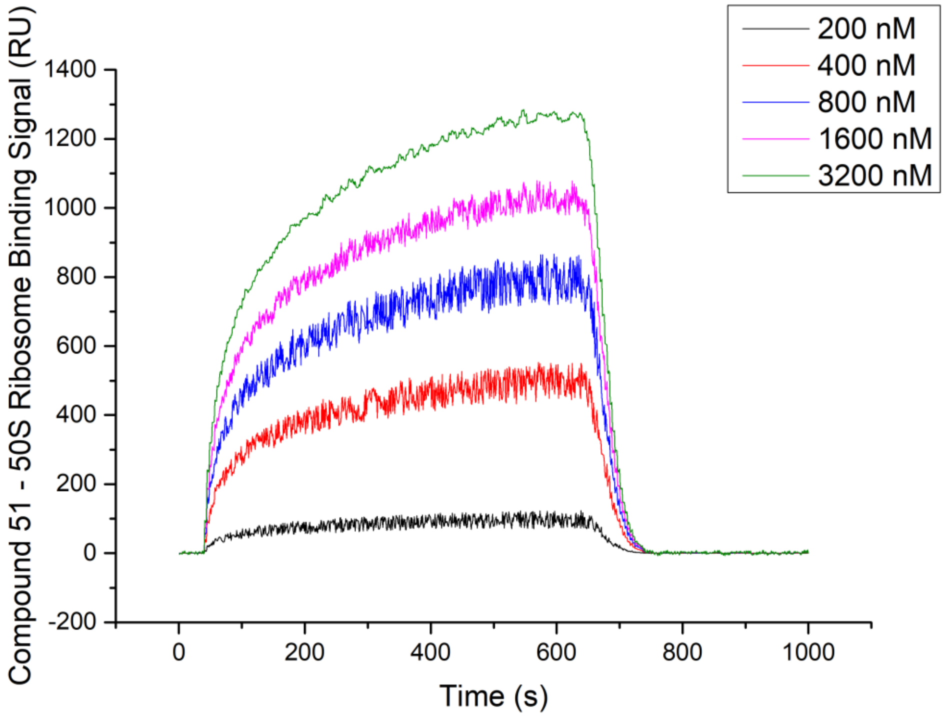

Figure SI 28. Concentration gradient curve of SPR binding signal between compound **51** and *50S* ribosome.

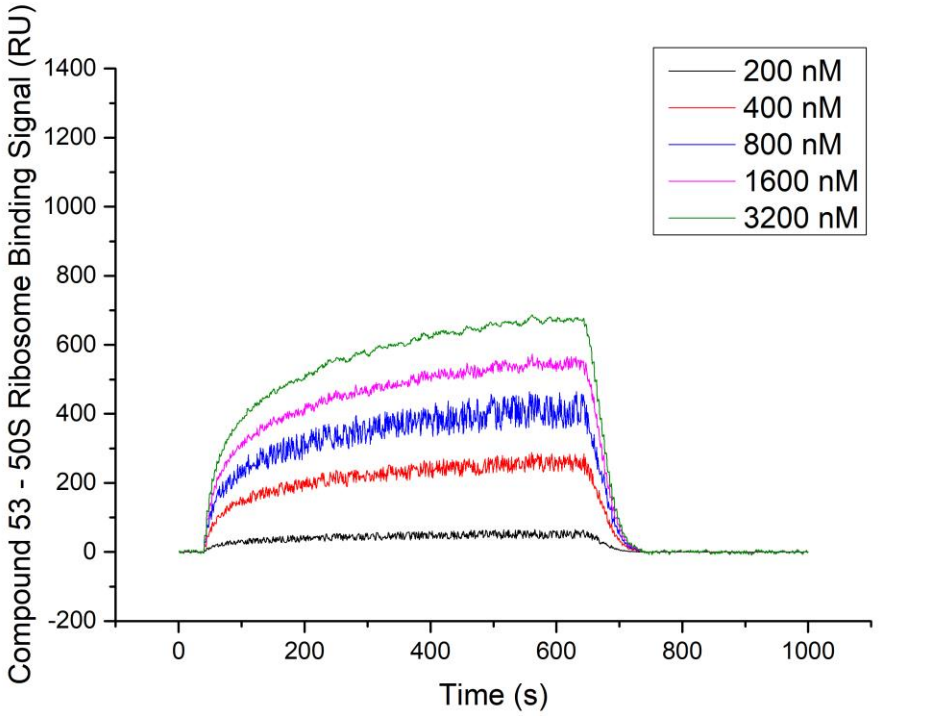

Figure SI 29. Concentration gradient curve of SPR binding signal between compound **53** and *50S* ribosome.

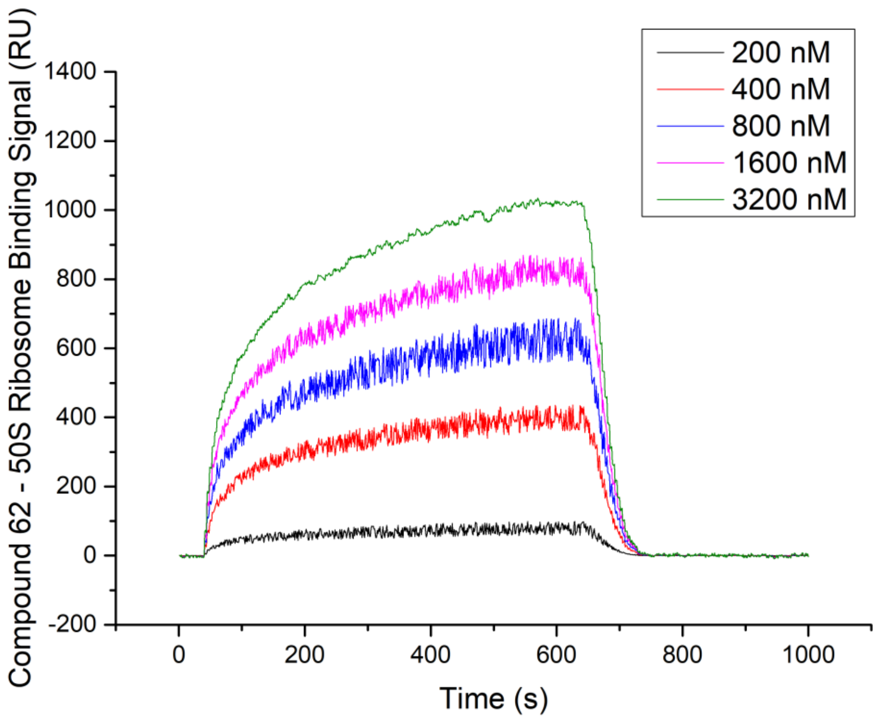

Figure SI 30. Concentration gradient curve of SPR binding signal between compound **62** and *50S* ribosome.

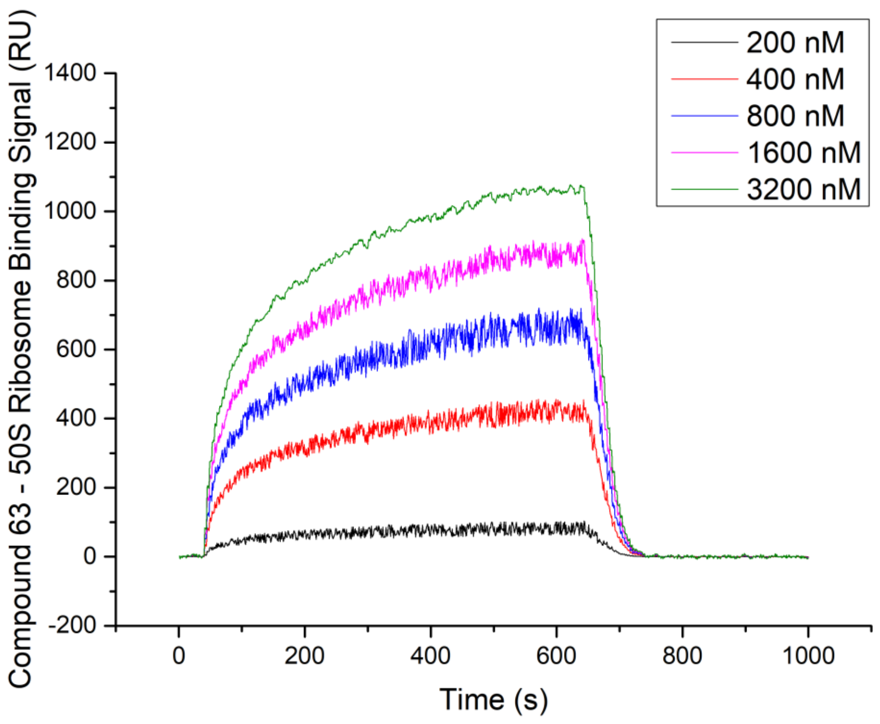

Figure SI 31. Concentration gradient curve of SPR binding signal between compound **63** and *50S* ribosome.

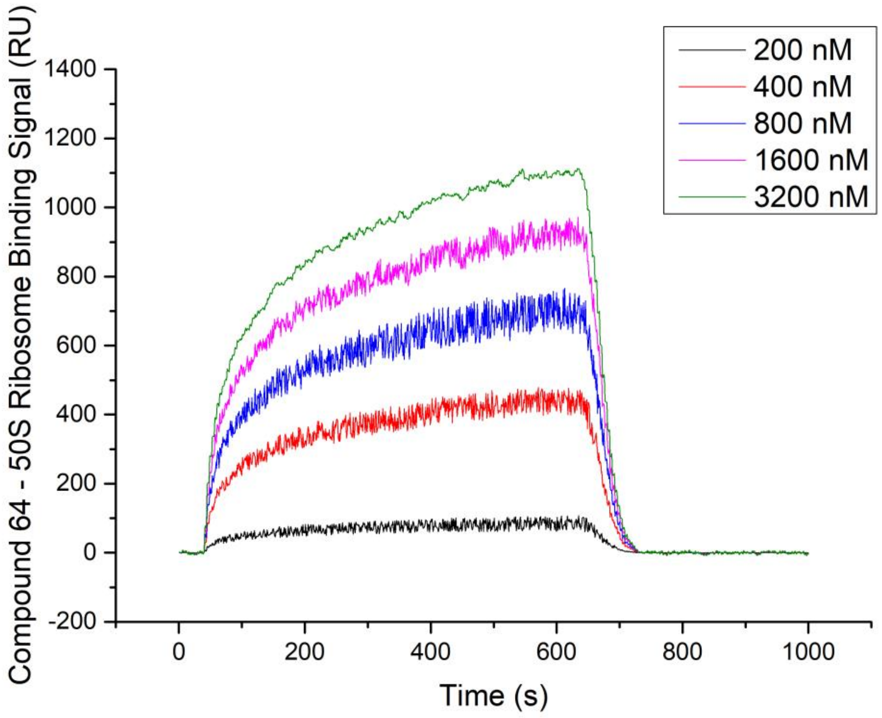

Figure SI 32. Concentration gradient curve of SPR binding signal between compound **64** and *50S* ribosome.

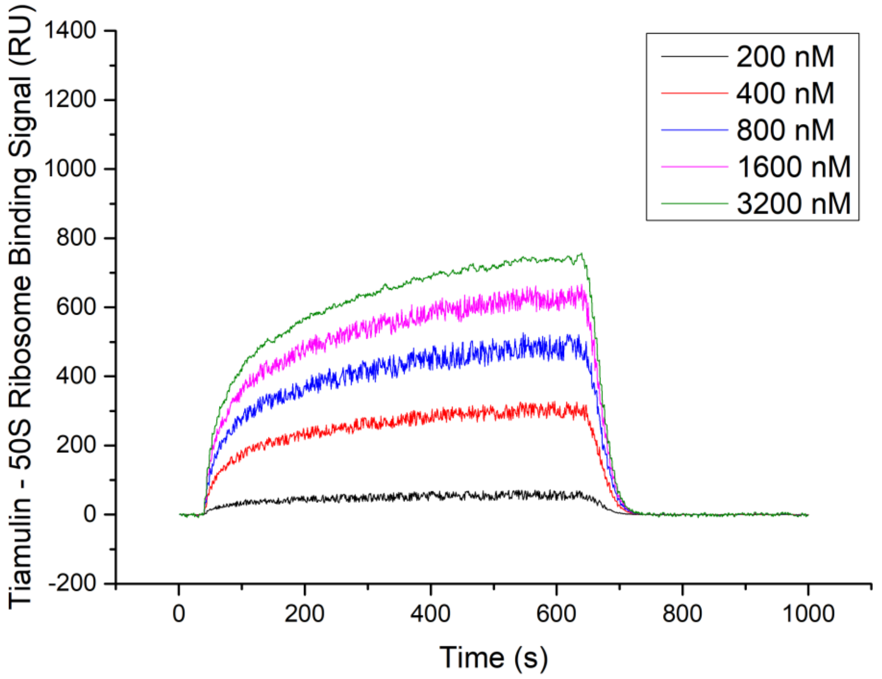

Figure SI 33. Concentration gradient curve of SPR binding signal between tiamulin and *50S* ribosome.

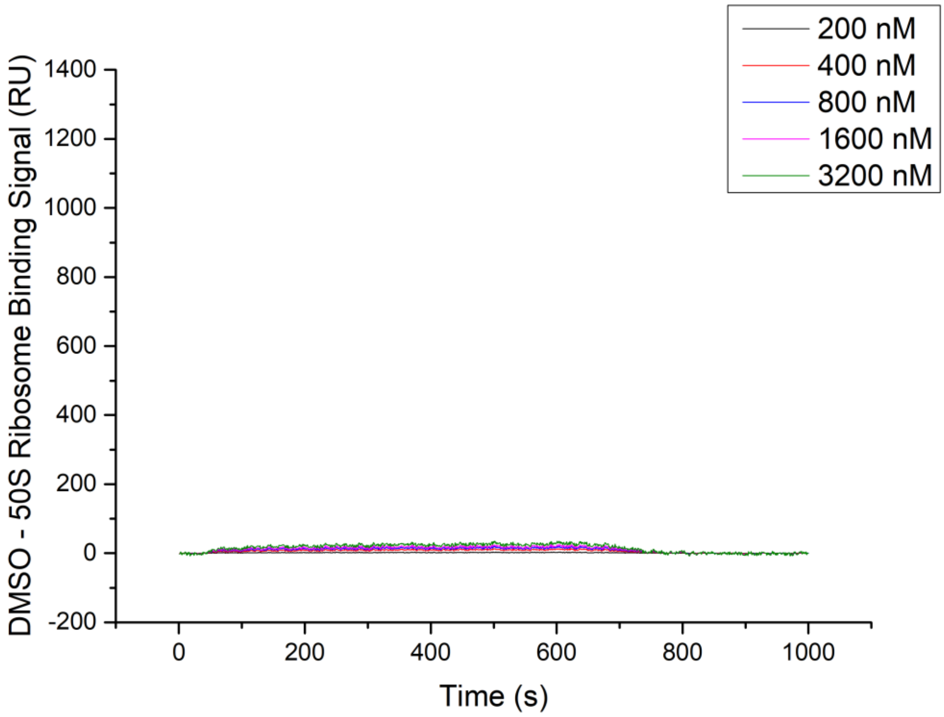

Figure SI 34. Concentration gradient curve of SPR binding signal between DMSO and *50S* ribosome.

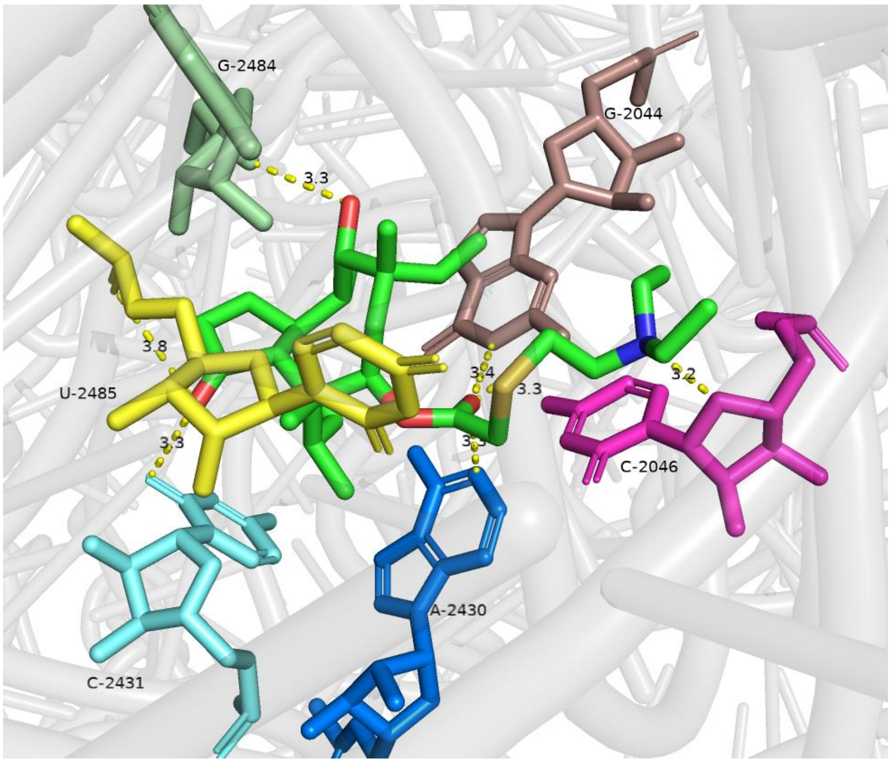

Figure SI 35. Docking mode of tiamulin (green) to *50S* ribosome (1XBP).
